# Alkamides Activate Jasmonic Acid Biosynthesis and Signaling Pathways and Confer Resistance to *Botrytis cinerea* in *Arabidopsis thaliana*

**DOI:** 10.1371/journal.pone.0027251

**Published:** 2011-11-04

**Authors:** Alfonso Méndez-Bravo, Carlos Calderón-Vázquez, Enrique Ibarra-Laclette, Javier Raya-González, Enrique Ramírez-Chávez, Jorge Molina-Torres, Angel A. Guevara-García, José López-Bucio, Luis Herrera-Estrella

**Affiliations:** 1 Instituto de Investigaciones Químico-Biológicas, Universidad Michoacana de San Nicolás de Hidalgo, Morelia, Michoacán, México; 2 Laboratorio Nacional de Genómica para la Biodiversidad, Cinvestav Irapuato, Irapuato, Guanajuato, México; 3 Departamento de Biotecnología y Bioquímica, Unidad Irapuato, Cinvestav, Irapuato, Guanajuato, México; 4 Instituto de Biotecnología-UNAM, Cuernavaca, Morelos, México; 5 Centro Interdisciplinario de Investigación para el Desarrollo Integral Regional-IPN, Guasave, Sinaloa, México; Oregon State University, United States of America

## Abstract

Alkamides are fatty acid amides of wide distribution in plants, structurally related to *N*-acyl-L-homoserine lactones (AHLs) from Gram-negative bacteria and to *N*- acylethanolamines (NAEs) from plants and mammals. Global analysis of gene expression changes in *Arabidopsis thaliana* in response to *N*-isobutyl decanamide, the most highly active alkamide identified to date, revealed an overrepresentation of defense-responsive transcriptional networks. In particular, genes encoding enzymes for jasmonic acid (JA) biosynthesis increased their expression, which occurred in parallel with JA, nitric oxide (NO) and H_2_O_2_ accumulation. The activity of the alkamide to confer resistance against the necrotizing fungus *Botrytis cinerea* was tested by inoculating *Arabidopsis* detached leaves with conidiospores and evaluating disease symptoms and fungal proliferation. *N*-isobutyl decanamide application significantly reduced necrosis caused by the pathogen and inhibited fungal proliferation. *Arabidopsis* mutants *jar1* and *coi1* altered in JA signaling and a MAP kinase mutant (*mpk6*), unlike salicylic acid- (SA) related mutant *eds16/sid2-1*, were unable to defend from fungal attack even when *N*-isobutyl decanamide was supplied, indicating that alkamides could modulate some necrotrophic-associated defense responses through JA-dependent and MPK6-regulated signaling pathways. Our results suggest a role of alkamides in plant immunity induction.

## Introduction

Plants continuously respond to abiotic and biotic stress by adjusting their metabolism and activating diverse intracellular and systemic responses. Biotic stress induced by pathogens triggers complex signaling cascades regulated by hormones once an invader has been detected. Three main phytohormones have been classically recognized as essential components of responses triggered by pathogens, namely salicylic acid (SA), jasmonic acid (JA) and ethylene (ET). Hormonal-dependent pathways result in the expression of defense-related genes such as those encoding pathogenesis-related (PR) proteins, and the production of antimicrobial secondary metabolites [1]. These responses are assisted by reactive molecules, such as nitric oxide (NO) and reactive oxygen species (ROS) that function both, as signaling components of transcriptional and metabolic readjustment and as antimicrobial substances [2]. Lifestyle of pathogens largely determines the effectiveness of a plant-induced response to combat the pathogen challenge. The SA-dependent signaling pathway is often considered to be effective against pathogens that derive nutrients from living hosts cells (biotrophs), and JA/ET pathways against pathogens that derive nutrients from dead cells (necrotrophs), although, the persistence of defense responses and the disease outcome are determined by complex networks of interactions between multiple hormone signaling pathways [3].

Lipids have a key role in maintaining the fluidity and structural integrity of all cell membranes. Additionally, lipids and various fatty acid derivatives have been described to act as signaling molecules in response to diverse environmental cues [4]. Structural features of fatty acids, such as the chain length and their unsaturation degree, determine their function and biological activity by altering membrane lipid composition [5]. Exogenous and endogenous mono- and poly-unsatured fatty acids (PUFAs) alter plant gene expression and metabolism, thus impacting the plant-microbe and plant-herbivore interactions [5], [6]. For instance, alterations in enzymatic machinery that regulates production of cellular unsaturated fatty acids alter the SA- and JA-mediated defense signaling. A reduction in the endogenous levels of oleic acid (18∶1) caused by mutation of a gene encoding STEAROYL-ACYL CARRIER PROTEIN-DESATURASE, increases the expression level of *PR* genes in a SA-dependent way, but at the same time, reduces expression of a subset of JA-dependent response genes and decreases resistance to *Botryris cinerea* in *Arabidopsis* [7]. A kind of PUFAs that impact plant defense responses are the eicosapolyenoic acids, which are produced and released by several species of oomycete plant pathogens. Specifically, exogenous application of arachidonic acid (20∶4) to *Arabidopsis* and tomato (*Solanum lycopersicum*) plants induces expression of general-stress responsive genes, increases endogenous JA levels and confers resistance against the necrotrophic fungi *Botrytis cinerea* [8]. Based on this evidence, it has been proposed that lipids and their derivates have transorganismal signaling activity, and that their dependent pathways are conserved throughout the evolutive history of organisms [8].

Commonly, the full range of biological effects triggered by PUFA signals, are carried out by their metabolism into more potent substances, the oxylipins. Oxylipins are a diverse class of lipid metabolites that include fatty acid hydroxiperoxides, hydroxy-, oxo-, or keto-fatty acids, volatile aldehydes, or mostly, jasmonates (JAs) [9]. JAs act as regulatory molecules in metabolic and developmental processes, as well as in defense responses [10], [11], [12]. They rapidly accumulate by wounding, insect attack and necrotrophic pathogen infection [13]. JA is synthesized through a series of reactions involving lipoxygenases (LOXs), allene oxide synthases (AOSs), allene oxide cyclases (AOCs) and 12-oxophytodienoate reductases (OPRs). Then, JA is further modified to produce JAs, for example, as conjugates with various lipophilic amino acids such as isoleucine (Ile) produced by a jasmonate amino acid synthetase, encoded by *JASMONATE RESISTANT1* (*JAR1*). The JA signal (JA-Ile) is perceived by an intracellular receptor, the F-box protein COI1, which plays a key role in JA signaling [14], and is required for the majority of the JA-mediated responses described to date, such as fertility, secondary metabolite biosynthesis, pest and pathogen resistance, and wound responses [15]. COI1 is an E3 ubiquitin ligase that catalyzes the ubiquitination of proteins destined to degradation via the proteosome-mediated pathway. COI1 activates a signal transduction pathway that culminates in the transcriptional activation or repression of JA-responsive genes. The *coi1* mutant is resistant to JAs and to the *Pseudomonas syringae* toxin coronatine. The essential role of JAs in plant immunity is also evidenced by JA-related mutant phenotypes, for example both *jar1* and *coi1* show an enhanced susceptibility to necrotrophic pathogens [16], [17]. In addition, protein phosphorylation and dephosphorylation have important roles in JA signaling. The mitogen-activated protein kinase (MAPK) cascade, which is one of the major signal transduction pathways in plants, as well as other eukaryotes, has been found to be regulated by JA to modulate JA-dependent gene expression [17]. In Arabidopsis, three MAPKs (MPK3, MPK4 and MPK6) have been implicated in defense against pathogens [18], [19], [20]. MPK6 functions as substrate of at least four MAPK kinases (MKK2, MKK3, MKK4 and MKK5) in response to different stimuli, including developmental, microbial or environmental cues. Once phosphorylated, MPK6 activates several transcriptional regulators, such as members of the WRKY, MYC and ERF gene families. Particularly, but not exclusively, the MKK3-MPK6 cascade is activated in response to JA and both, positively and negatively regulates the expression of JA-related genes [17], [21]. Concordantly, the *MKK3*-knockout mutant *mkk3* and *coi1* had an altered activation of MPK6 in response to JA. Moreover, mutations in *MPK6* compromise the accumulation of antifungal phytotoxin camalexin in response to infection with *Botrytis cinerea* [17], [22].

An additional class of lipids conserved among different kingdoms with signaling functions in plants is the fatty acid amides group, including the plant-, fungal- or animal-produced *N*-acylamides (alkamides), *N*-acylethanolamines (NAEs), and the *N*-acyl-L-homoserine lactones synthesized by Gram-negative bacteria. Compounds representative of these three classes of lipids have been shown to modulate seedling development in *Arabidopsis* and to affect plant biomass production in a dose-dependent way, indicating a strong biological activity [23], [24], [25]. NAEs are compounds with aminoalcohol linked as an amide to the fatty acid, which accumulate in seeds of higher plants, including cotton, corn, soybean, tomato, pea and *Arabidopsis*, and decrease during germination [26]. Pioneering research on fatty acid amides showed that NAE production in plants is associated to defense responses. NAE 14∶0 accumulates in tobacco leaves treated with fungal elicitors, and exogenous application of this NAE is able to induce expression of defense-related genes [27]. Moreover, the ectopic overexpression of a plant fatty acid amide hydrolase (FAAH), an enzyme that degrades NAEs, renders *Arabidopsis* plants more susceptible to both host and non-host bacterial pathogens [28]. *N*-acyl-L-homoserine lactones (AHLs) are structural analogs of NAEs and alkamides, that are produced by Gram negative bacteria and participate in the cell-to-cell communication process commonly referred to as quorum-sensing (QS). Interestingly, plants have the genetic machinery to perceive and respond to AHLs. The presence of AHL-producing bacteria in the rhizosphere of tomato induces SA- and JA-dependent defense responses, conferring resistance to the fungal pathogen *Alternaria alternata* [29]. Moreover, the application of purified AHLs to *Medicago truncatula* and *Arabidopsis* plants results in differential transcriptional changes in roots and shoots, affecting expression of genes potentially involved in immune responses and development [30], [31]. Interestingly, FAAH knockouts and overexpressors *Arabidopsis* lines are more sensitive and tolerant, respectively, to the root inhibitory effects of AHLs, in a similar fashion to their response to exogenous NAEs and alkamides, while an alkamide resistant mutant termed *decanamide resistant root 1* (*drr1*) showed decreased root responses to alkamides and AHLs [25], [32].

Alkamides comprise over 200 related compounds and they have been found in several plant families: Aristolochiaceae, Asteraceae, Brassicaceae, Convolvulaceae, Euphorbiaceae, Menispermaceae, Piperaceae, Poaceae, Rutaceae, and Solanaceae, reviewed in [33], [34]. Some traditional medicinal plants produce these secondary metabolites during their life cycle in response to several stress conditions to mediate, among other processes, plant chemical defense against plant competitors or microbial and herbivorous predators [35]. Several species from the genus *Echinacea* accumulate unsaturated alkamides ranging from 12 to 18 carbon atoms in response to JAs [36], [37]. These unsatured alkamides are also active in mammals; they activate immune responses in alveolar macrophages from rats, in concert with a sustained production of NO, a canonical messenger in plant and animal defense responses [38], [39]. Alkamides have also been identified in insects, such as *N*-linolenoyl-L-glutamine, present in oral secretions of the tobacco hornworm (*Manduca sexta*), which is able to elicit defensive responses in plants by inducing volatile chemicals that attract predators and parasites of the attacker [40]. The wound-induced JA production is amplified by application of these oral secretions in *Nicotiana attenuata* leaves, indicating a reciprocal crosstalk between JAs- and alkamides-related signal pathways [41]. To date, however, there is no direct evidence as to whether alkamides can switch JA production and its transcriptional targets.

The short-chain alkamide affinin from the “gold-root” *Heliopsis longipes* has been reported to have antimicrobial activity inhibiting in vitro growth of some plant microbial pathogens, including bacteria and fungi [35]. To explore the structure-activity relationships of alkamides, we previously evaluated the root developmental responses of *Arabidopsis* seedlings to application of a group of affinin-derived natural and/or synthetic fatty acid amides with similar chain length [42]. We found that *N*-isobutyl decanamide, a C:10 saturated alkamide that is naturally produced in *Acmella radicans* [43] and *Cissampelos glaberrima* [44], was the most active compound in inhibiting primary root growth and stimulating lateral root formation. Interestingly, root developmental alterations induced by *N*-isobutyl decanamide were related to a sustained increase in nitric oxide (NO) production and required the activity of the *DRR1* protein [45].

To further understand the molecular responses to fatty acid amides, in this work we performed whole-genome transcriptional profiling of *Arabidopsis thaliana* seedlings in response to *N*-isobutyl decanamide. Our results show the activation of defense-related gene expression, concomitant to an increase in JA accumulation and in the expression of JA-responsive and senescence-associated genes. Moreover, *N*-isobutyl decanamide application to mature *Arabidopsis* leaves conferred resistance against fungal necrotizing pathogen *Botrytis cinerea* in a process involving JA-dependent signaling.

## Results

### Transcriptomic profiling of *Arabidopsis* in response to *N*-isobutyl decanamide

To characterize at the transcriptional level the molecular responses of *Arabidopsis* to *N*-isobutyl decanamide, Col-0 WT seedlings were germinated and grown for 6 d on 0.2× MS medium and then transferred to fresh medium supplied with or without 60 µM of *N*-isobutyl decanamide to directly compare their effect on whole-genome transcriptional profile after 1, 3, 7 and 14 d of treatment (Figure S1) employing a two-channel long-oligonucleotide microarray platform (see Methods).

**Figure 1:**
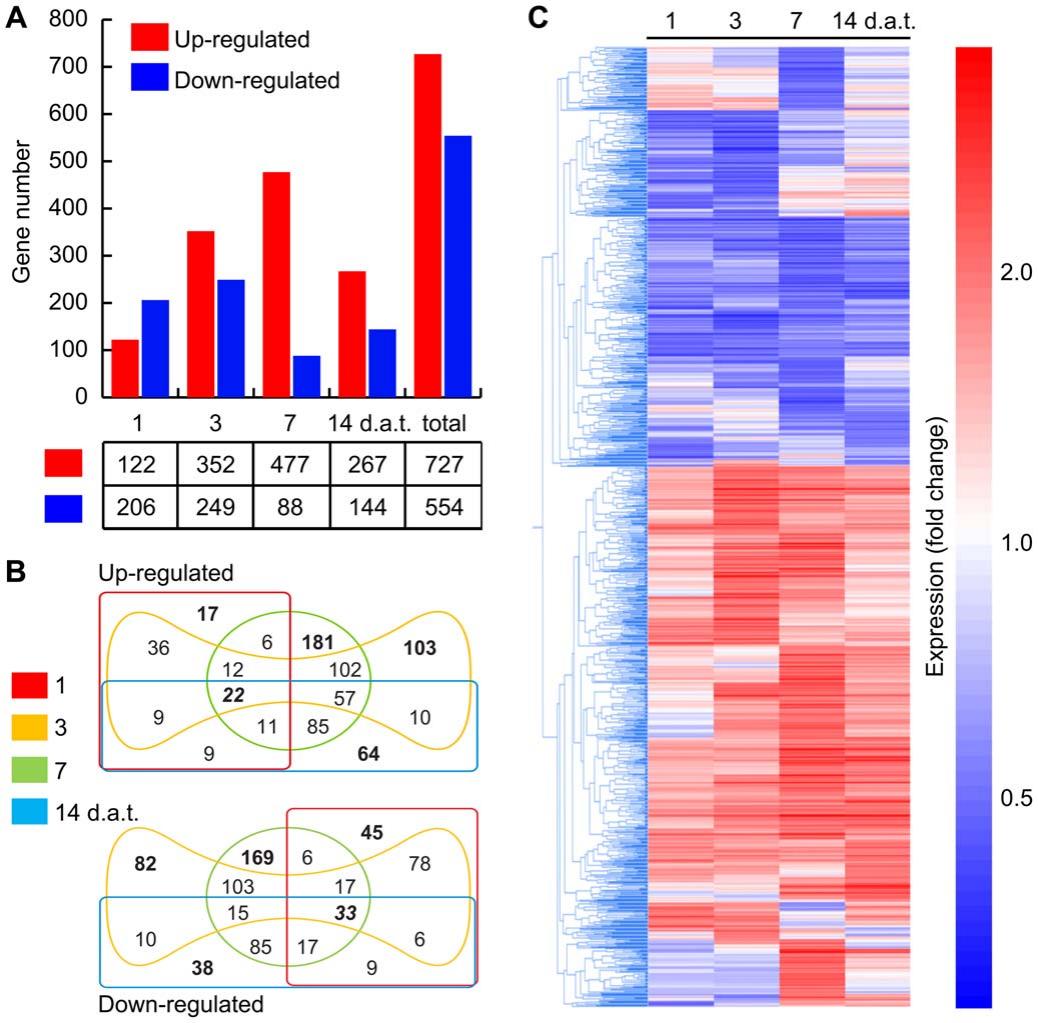
Overview of *N*-isobutyl decanamide responsive genes in *Arabidopsis* seedlings. Number of genes (vertical axis) Up-regulated (red) and Down-regulated (blue) by *N*-isobutyl-decanamide treatment at 1, 3, 7, and 14 d.a.t. (A). Edwards-Venn diagrams showing common or distinct responsive genes identified at every time evaluated (B). The number of genes up- or down-regulated in a single condition is shown in bold letters. The number of genes regulated at all sampled-times are shown in bold italic font. Agglomerative hierarchical clustering of differentially expressed genes at every sampled times (C). Clustering was performed using the Smooth correlation and average linkage clustering in GeneSpring GX 7.3.1 software (Agilent Technologies®). Blue color indicates Down-regulated, red Up-regulated and white unchanged values, as shown on the color scale at the right side of the figure.

According to a stringency level of FDR 0.05 (fold change ≥2), a total of 1,281 genes showed differential expression in at least one of the four sampled time points. The complete list of differentially expressed genes is provided in Table S1. Among differentially expressed genes, 727 were found to be up-regulated and 554 down-regulated by *N*-isobutyl decanamide (Figure 1A). Only 22 from the 727 induced genes and 33 down-regulated genes were common to all time points evaluated (Figure 1B). Of these overlapping genes, highest expression values were reached on the seventh day of *N*-isobutyl decanamide treatment (Figure 1C). Analysis of expression patterns by agglomerative hierarchical clustering showed that the number of differentially regulated genes increased from day 1 to day 7 after treatment and then decreased at day 14 (Figure 1C).

In addition to the statistical methods described (see Materials and Methods), validation of microarray data was achieved by real-time quantitative PCR (qRT-PCR) of 15 randomly chosen genes, including up- and down-regulated genes. These experiments were carried out using RNA extracted from an independent batch of control and treated plants than those used for microarray analysis experiments. qRT-PCR gene expression profiles obtained for the analyzed loci were quite consistent with those generated by the microarray analysis (Figure S2).

### Functional categories of genes up-regulated by *N*-isobutyl decanamide

Differentially expressed genes were classified into functional categories according to the Munich Information Center for Protein Sequences classification (MIPS) using the FunCat database [46]. The categories “Metabolism” (290 genes), “Storage protein” (16 genes), “Cellular transport, transport facilities and transport routes” (161 genes), “Cell rescue, defense and virulence” (189 genes), “Interaction with the environment” (165 genes), “Systemic interaction with environment” (92 genes), “Cell fate” (31 genes) and “Biogenesis of cellular components” (90 genes) were identified as significantly over-represented MIPS categories among *N*-isobutyl decanamide responsive genes (Table S2). Most of these genes belong to defense- and stress-related categories, including subcategories belonging to the “Metabolism” set such as, ‘Metabolism of glutamate, polyamines, nitrogen and related groups, chitin and others polysaccharides, and secondary metabolism’ (Table S2).

**Figure 2:**
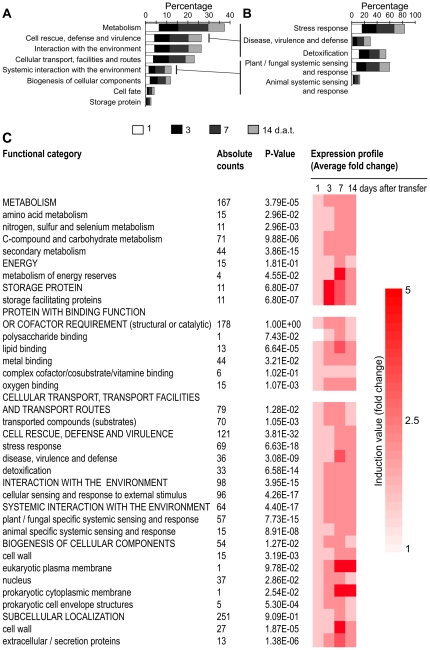
Functional classification of *Arabidopsis* genes differentially expressed in response to *N*-isobutyl decanamide treatment. Categorization of 1,281 genes regulated by treatment was obtained according to the Munich Information Center for Protein Sequences classification (MIPS) classification using FunCat database (http://mips.helmholtz-muenchen.de/proj/funcatDB/) and *Arabidopsis* annotation. Statistically significant categories were identified by using a hypergeometric method and Bonferroni correction with a cutoff of p-value >0.05 (A). MIPS defense-related subcategories significantly represented (B). Percentages relate to total differentially regulated genes per sampled times. The average transcriptional change of all induced genes in overrepresented functional categories was also calculated along time of treatment (C).

**Figure 3:**
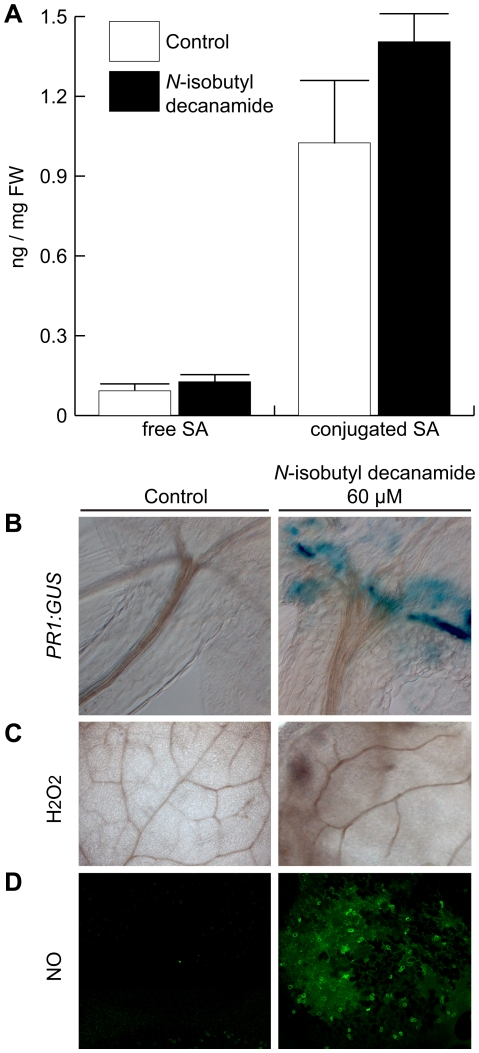
Effects of *N*-isobutyl decanamide on defense-related metabolite production and *PR1* expression. *Arabidopsis* seedlings were grown for 6 days on *N*-isobutyl decanamide-free medium and transferred to control plates with or without *N*-isobutyl decanamide for 7 additional days. Salicylic acid (SA) accumulation was determined by measuring free and conjugated SA by GC-MS (A). Benzoic acid was used as internal standard, data are means of three independent experiments ± SD. Transgenic *Arabidopsis* line carrying *PR1∶GUS* was stained for *GUS* expression (B). Detection of hydrogen peroxide (H_2_O_2_) was made by staining leaves from control and treated seedlings with DAB (C). Images were captured with a Nomarski microscope. Nitric oxide (NO) from leaves of control and treated seedlings was detected by analyzing fluorescent signal of DAF-2DA with a confocal microscope (D). All photographs are representative individuals from at least 9 seedlings analyzed.

When we performed functional categorization per day of treatment, we found that the highest percentage of genes in every category was represented at the seventh day (Figure 2A). Two remarkable over-represented categories identified were “Cell rescue, defense and virulence” and “Systemic interaction with environment” (Figure 2A). Detailed analyses of these two categories showed significant overrepresentation of the ‘stress response’, ‘disease, virulence and defense’, ‘detoxification’, ‘plant/fungal specific systemic sensing and response’ and ‘animal systemic sensing and response’ subcategories (Figure 2B). Within these subcategories, we found 70 genes involved in oxygen and radical detoxification, 75 genes involved in hormone-related responses (auxin, ethylene, cytokinin and abscisic acid), and particularly, genes encoding enzymes involved in JA synthesis and associated responses (Table S2; Figure 3). Additional differentially regulated genes encoded proteins related to biotic stress, including different secreted pathogenesis-related proteins (PR) such as chitinases and glucanases (At4g07820, At2g19990, At2g14610, At3g57260, At3g04720, At1g75040, At2g19970, At2g14580, At4g33720 and At2g14580) (Table S1). Overrepresentation of biotic stress-related categories can be appreciated more clearly in the functional categorization of *N*-isobutyl decanamide-induced genes (Figure 2C). These results suggest that alkamides are likely involved in triggering defense-associated responses in *Arabidopsis*.

### General defense responses but not salicylic acid biosynthesis are activated by *N*-isobutyl decanamide

Because *N*-isobutyl decanamide increased the transcript level of a wide class of PR genes, we examined its effect on the production of salicylic acid (SA) and signaling molecules related to local and systemic responses in defense processes. SA is a phenolic hormone whose activity is required to successfully respond against several different invading pathogens [47] and their biosynthesis succeeds in association with changes in redox homeostasis producing reactive oxygen species (ROS) such as superoxide and hydrogen peroxide (H_2_O_2_) [48]. In turn, SA and H_2_O_2_ release is accompanied by another reactive signalling molecule, nitric oxide (NO). Whole-transcriptional profiling regulated by *N*-isobutyl decanamide showed that *PATHOGENESIS-RELATED1* (*PR1*, At2g14610), a marker for SA signaling, and overall defense responses [49], [50], [51] increased its transcript level by 7.5-fold at day 7 (Table S1). However, none of the genes encoding enzymes related to SA biosynthesis were significantly up-regulated. Moreover, *N*-isobutyl decanamide did not appear to significantly affect the overall SA content despite an observed induction of the *PR1∶GUS* reporter-gene expression (Figure 3A &B), suggesting that *N*-isobutyl decanamide-mediated gene expression of *PR1* occurred independently of SA accumulation.

It is well documented that some stress-associated molecules, such as ROS, play signaling roles as second messengers in developmental and defense process. Among the *N*-isobutyl decanamide differentially expressed genes, at least 70 belonging to the functional group “oxygen and radical detoxification” were regulated by alkamide treatment (Table S2), having their highest expression level at days 3 and 7 after transfer. Up-regulated genes included ten peroxidases (At4g08780, At5g06730, At4g08770, At5g06720, At5g58390, At5g05340, At2g18150, At3g49960, At3g49110 and At2g18140), two thioredoxin H-type *TH8* and *TH7* (At1g69880, At1g59730) and a glutaredoxin (At5g40370), two glutathione peroxidases *ATGPX4* and *ATGPX6* (At2g48150, At4g11600), five FAD-binding oxidoreductases (At1g26410, At1g26380, At1g26390, At1g26400 and At1g26420), the catalase *CAT3* (At1g20620) and *HYDROPEROXIDE LYASE1* (At4g15440) (Table S1 & S2). Given this overrepresentation, we decided to explore whether ROS accumulation coincided with the increase in transcript level of the group of oxygen and radical detoxification genes. We detected hydrogen peroxide (H_2_O_2_) production in situ in *Arabidopsis* seedlings that were transferred for 7 d from MS 0.2× medium to a medium containing *N*-isobutyl decanamide. At this stage the seedlings were treated with 3,3-diaminobenzidine (DAB), which in the presence of peroxidases polymerizes as soon as it comes into contact with H_2_O_2_, forming a brown precipitate. Leaves from *N*-isobutyl decanamide-treated seedlings clearly showed an increase in H_2_O_2_ (Figure 3C) and NO production (Figure 3D) when compared to solvent-treated seedlings. Overall, these results suggest that general defense-associated responses elicited by *N*-isobutyl decanamide appear to be related to both hormonal and oxidative stress response.

### Endogenous levels of JA and their corresponding transcripts are induced by *N*-isobutyl decanamide

**Figure 4:**
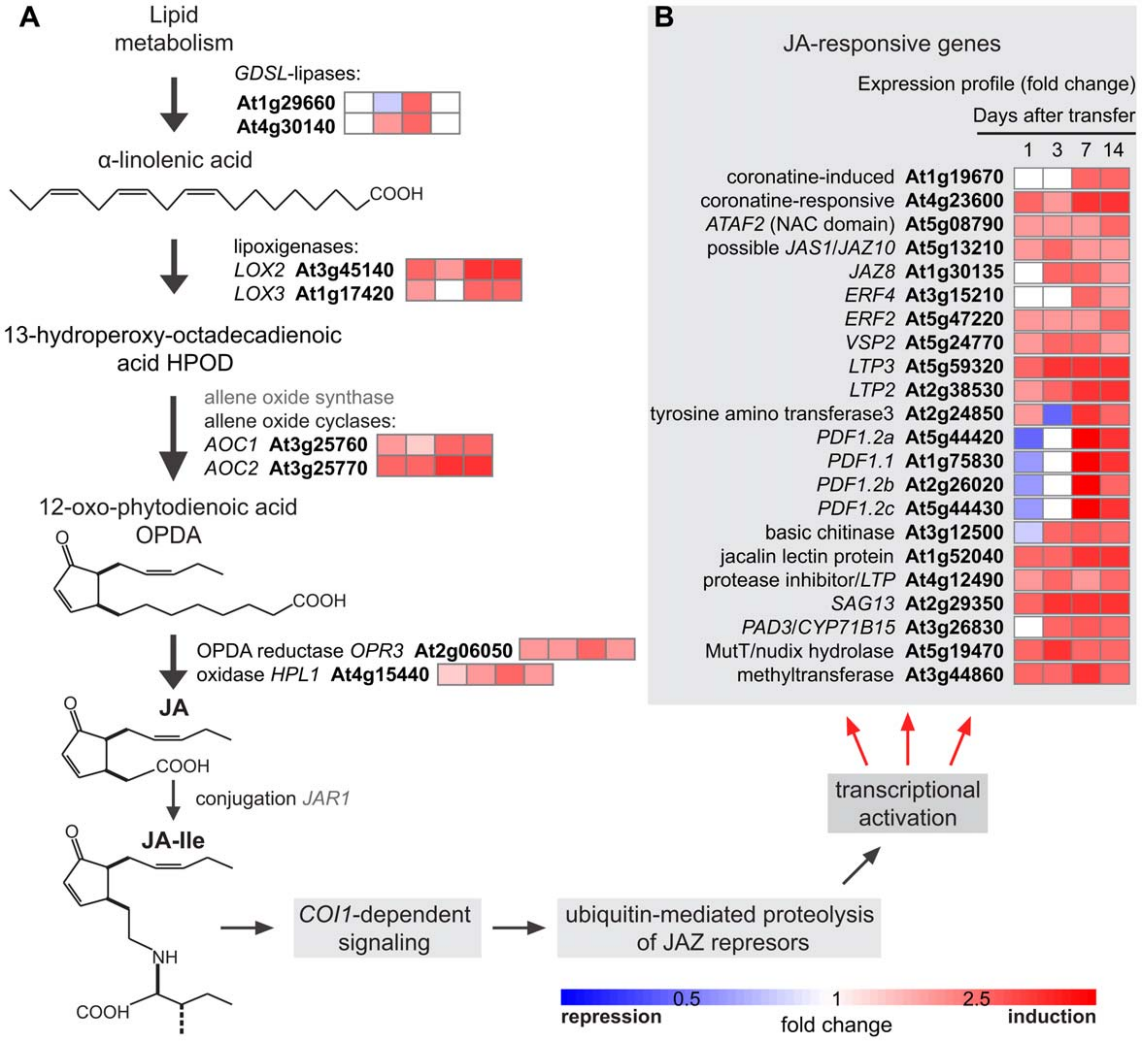
JA-related pathways are transcriptionally induced by *N*-isobutyl decanamide. Simplified representation of JA biosynthetic pathway, genes and metabolites are illustrated (A). Fold-change values of JA-biosynthetic and –responsive pathway genes differentially expressed with *N*-isobutyl decanamide treatment (A, B). Data from microarray expression profiles are shown in the color scale from blue to red.

Virtually all genes encoding for biosynthetic enzymes for JA production were regulated by *N*-isobutyl decanamide (Figure 4A). Canonical JA-dependent inducible genes involved in JA signaling and response pathways showed predominantly induction profiles (Figure 4C). Among them *CORONATINE-INDUCED3* (*CORI3*, At4g23600), *NAC DOMAIN-CONTAINING PROTEIN81* (*ATAF2*/*ANAC081*, At5g08790), *JASMONATE-ZIM-DOMAIN* (*JAS1*/*JAZ10*, At5g13210), *ETHYLENE RESPONSE FACTOR2* (*ERF2*, At5g47220), *VEGETATIVE STORAGE PROTEIN2* (*VSP2*, At5g24770), *LIPID TRANSFER PROTEINS* (*LTP3*, *LTP2* and *LTP*, At5g59320, At2g38530, and At4g12490, respectively) and the *SENESCENCE-ASSOCIATED GENE13* (*SAG13*, At2g29350) sustained high expression values through our temporal kinetic experiment. The genes *CORI1* (At1g19670), *JAZ8* (At1g30135), *ERF4* (At3g15210) and *PHYTOALEXINE DEFICIENT3* (*PAD3*, At3g26830) showed increased expression from basal levels to induced expression levels. Four *PLANT DEFENSIN* genes *PDF1.2a*, *PDF1.1*, *PDF1.2b* and *PDF1.2c* (At5g44420, At1g75830, At2g26020, At5g44430) and a chitinase (*BASIC CHITINASE*, At3g12500) ranged from repression to induction values (Figure 4B). However, all of them, in a similar way to the genes of the JA biosynthetic pathway, were overexpressed at day 7 after alkamide treatment (Figure 4A &B).

**Figure 5:**
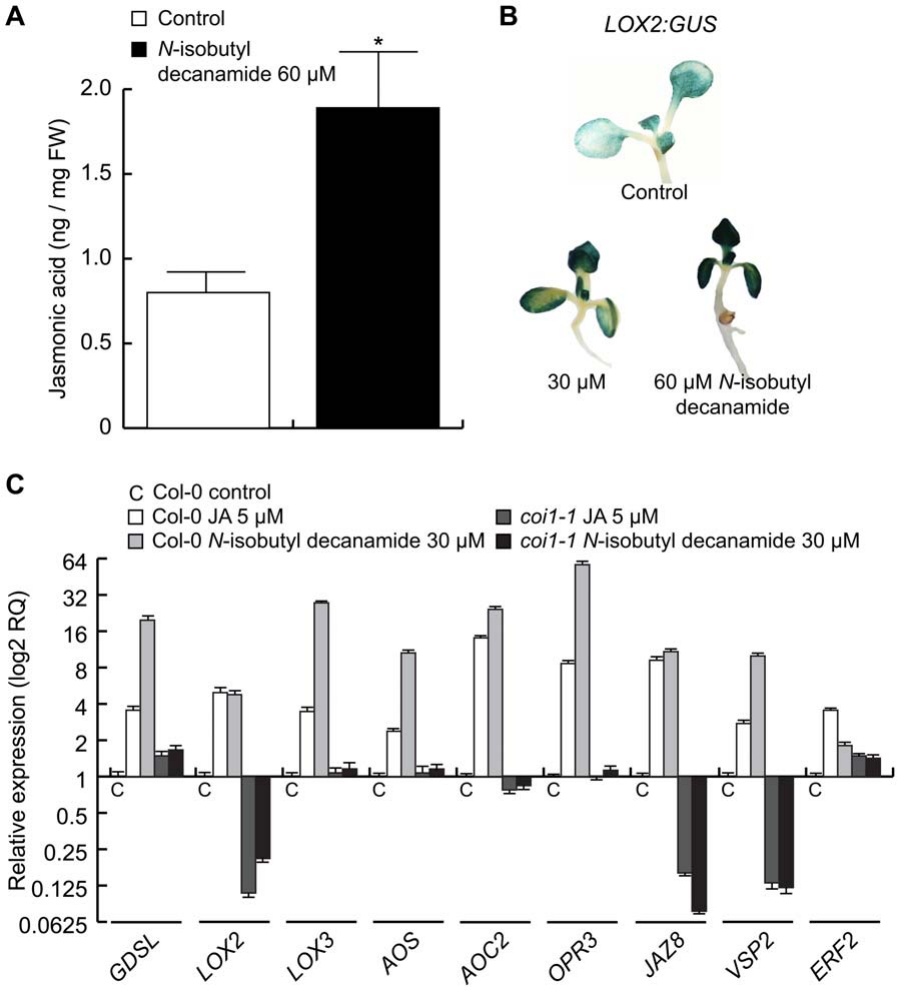
Levels of JA and their corresponding transcripts are enhanced by *N*-isobutyl decanamide. *N*-isobutyl decanamide-dependent accumulation of JA was determined by GC-MS from three biological replicate samples (A), data are means of three independent experiments ± SD, asterisks denote a significant difference from control seedlings (*P*≤0.05). Transgenic *Arabidopsis* seedlings expressing *GUS* under the regulation of the JA-induced *LOX2* promoter (*LOX2∶GUS*) were grown for 7 days on solidified medium supplied with the solvent (control) or with 30 and 60 µM *N*-isobutyl decanamide, and then stained for *GUS* expression (B). Quantitative real-time PCR (qRT-PCR) analysis of nine JA-responsive genes using C_T_ value of *ACT2/7* as internal expression reference (C). Relative expression values were normalized with endogenous levels from each transcript in Col-0 control seedlings. Bars represent ± SE from three independent biological replicates, and there were four technical replicates for qRT-PCR assay.

To determine whether increase in the transcript level of JA-related genes correlated with changes in endogenous levels of JA, we quantified, by gas chromatography coupled to mass spectrometry (GC-MS), JA accumulation in seedlings treated with the solvent or 60 µM *N*-isobutyl decanamide . A nearly two-fold increase in JA level was observed in response to alkamide treatment (Figure 5A), indicating that the effects of this alkamide on gene expression might be mediated, at least in part, by increasing JA production in the plant. To gain further insight into the transcriptional activation of JA-dependent responses to *N*-isobutyl decanamide, transgenic seedlings containing a chimeric gene in which the *LOX2* promoter is fused to the *GUS* reporter gene (*LOX2∶GUS*) were treated with 30 and 60 µM *N*-isobutyl decanamide, and GUS histochemical analysis performed in 7 d-old seedlings. Interestingly, increased expression of this marker was observed throughout the shoot in alkamide-treated seedlings in a dose-dependent manner (Figure 5B).

Because a concentration of 30 µM *N*-isobutyl decanamide was able to induce *LOX2* expression, we evaluate the expression levels of JA-responsive transcripts by qRT-PCR in seedlings treated for 7 days with this alkamide concentration. Among the biosynthetic genes, we focused on genes encoding the *LOX2* and *LOX3* lipoxigenases, *ALLENE OXIDE SYNTHASE* (*AOS*) and *ALLENE OXIDE CYCLASE2* (*AOC2*) enzymes, and *OPDA REDUCTASE3* (*OPR3*). We also examined the expression levels of the JA-inducible genes *JAZ8*, *VSP2* and *ERF2*. All tested genes were induced by *N*-isobutyl decanamide application more strongly than JA itself (Figure 5C). It has been reported that JA-responsive gene expression occurs in a short time after JA perception [52]. For this reason, the JA insensitive mutant *coronatine insensitive1* (*coi1-1*) was employed as negative control to determine if an intact JA signaling pathway was required for the long-term (7 days) response. As shown in Figure 5C, most tested genes either did not respond or were repressed in response to JA or to *N*-isobutyl decanamide treatment in the *coi1-1* mutant.

Additionally, we evaluated local activity of *N*-isobutyl decanamide to induce transcriptional activation of defense-related genes in fully developed leaves, which were excised and incubated for 24 h in media supplemented with 30 µM *N*-isobutyl decanamide. Alkamide-treated leaves showed increased expression of the *LOX2∶GUS* reporter gene in a dose-dependent manner as compared to untreated controls (Figure S3A). Moreover, the relative expression level of *OPR3*, *VSP2* and *PAD3* genes was at least two-fold higher in treated wild-type leaves than in the controls (Figure S3B). These results show that at least some transcriptional networks modulated by *N*-isobutyl decanamide are active even in detached tissue.

### *N*-isobutyl-decanamide confers resistance to fungal necrotizing pathogen *Botrytis cinerea*

**Figure 6:**
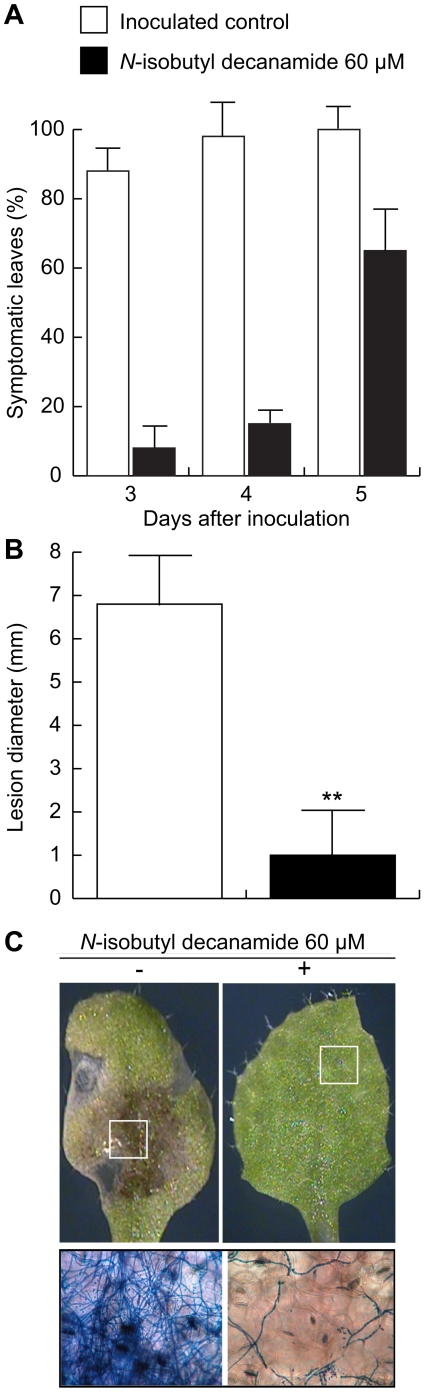
*N*-isobutyl decanamide confers protection against *Botrytis cinerea* attack. Leaves from 20 day-old plants grown in soil were pre-incubated 24 h on solvent (control, white squares), or 30 µM *N*-isobutyl decanamide containing plates (black squares), transferred to decanamide-free plates and then inoculated with a 10 µl droplet of *B. cinerea* spores (5×10^5^ conidiospores/ml). The percentage of leaves with necrotic symptoms at 3, 4 and 5 days after inoculation was determined (A). Bars mean ± SE of 30 inoculated leaves from two independent experiments. Tissue damage caused by *B. cinerea* was measured at 5 days after inoculation (B). Data points represent average lesion size ± SE from 30 independent leaves, asterisks denote a significant difference from control leaves (*P*≤0.05) as determined by *t* test. Representative inoculated leaves at 5 days after inoculation were imaged (C, top panels) and trypan blue-stained, inoculation sites are shown (C, bottom panels).

JA accumulation and JA-responsive gene expression analyses suggest that *N*-isobutyl decanamide may function as a potential defense-inducing factor. To determine whether *N*-isobutyl decanamide could effectively activate defense mechanisms that lead to pathogen resistance, we tested the responses of leaves from 20 d-old *Arabidopsis* plants to the necrotrophic pathogen *Botrytis cinerea*. In these experiments, again, fully developed leaves were transferred 24 h to agar plates supplied with 30 µM *N*-isobutyl decanamide or with the solvent as control. A 10 µl droplet of *B. cinerea* spores was inoculated on the leaf surface and disease symptoms evaluated 3, 4 and 5 days after inoculation (d.a.i.). In leaves transferred to control medium and inoculated for 3 days, the fungus induced necrotic lesions in over 90% of inoculated leaves (Figure 6A), whereas in *N*-isobutyl decanamide leaves treated only 10% presented necrotic lesions (Figure 6A). Four d.a.i., it was found that 100% of the control leaves showed necrotic lesions, whereas in *N*-isobutyl decanamide-treated leaves, around 15% and 60% of infected leaves showed necrotic lesions at fourth and fifth d.a.i., respectively. It is important to note that lesions in control leaves five d.a.i. were of about 6 mm in diameter, whereas in alkamide-treated leaves, the lesions had a diameter between 0.8 and 1.5 mm (Figure 6B). Visual inspection showed that after 5-d of inoculation, solvent-treated leaves inoculated with the pathogens presented generalized necrotic lesions spanning half or more the surface of the leaf, while *N*-isobutyl decanamide-treated leaves manifested significantly reduced symptoms (Figure 6C). We monitored hyphal growth of the pathogens by direct microscopic observation of stained mycelium in infected leaves. We found that disease symptoms in solvent-treated leaves at day 3 after inoculation were accompanied by prolific mycelium growth. In contrast, *N*-isobutyl decanamide treatment inhibited fungal growth over leaf surfaces, as compared to the control (Figure 6C). On the basis of these findings, it can be concluded that *N*-isobutyl decanamide treatment renders enhanced resistance to *B. cinerea* in *Arabidopsis* leaves.

To determinate if the reduced leaf damage and fungal growth inhibition observed in alkamide-treated leaves could be the result of a direct toxic effect of *N*-isobutyl decanamide on the fungal pathogen tested, we evaluated the antifungal activity of *N*-isobutyl decanamide on *B. cinerea* mycelial growth by inoculating mycelia disks on Petri plates containing PDA media supplemented with the solvent or with increasing concentrations of alkamide. Although 120 µM *N*-isobutyl decanamide inhibited mycelium growth by approximately 15%, any lower concentration had no significant effect (Figure S4). Indicating that plant defense responses elicited by *N*-isobutyl decanamide, and no an antifungal activity were responsible of pathogen proliferation over inoculated leaves.

### JA signaling is required for the *N*-isobutyl decanamide-induced resistance to *B. cinerea*

**Figure 7:**
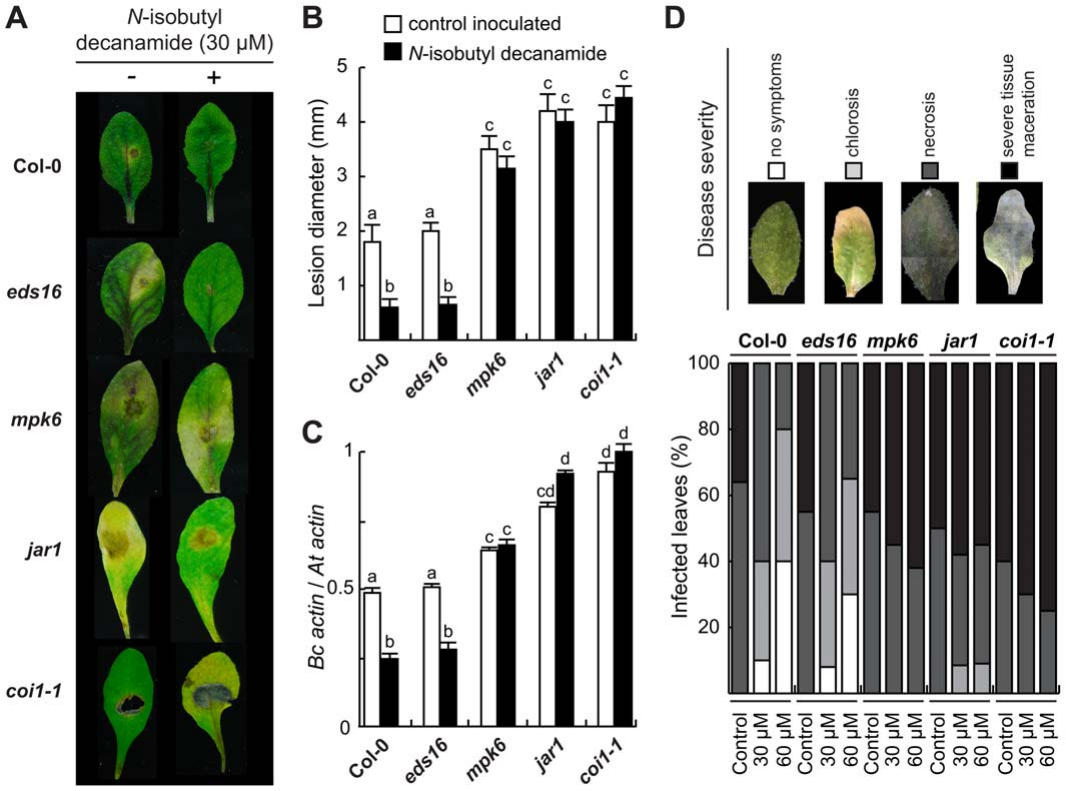
Effect of JA-related mutations on disease resistance response induced by *N*-isobutyl decanamide. (A–C) Disease symptoms on detached 20 day-old leaves at 4 days after drop inoculated with a 5 µl droplet as described in the legend for Figure 6 from wild-type (Col-0) plants, JA-related mutants *jar1*, *coi1-1* and *mpk6*, and the SA-deficient mutant *eds16/sid2-1*. Images show necrotic lesions (A), mean lesion size (B) and, in (C), fungal growth. The data show the qPCR amplification of *B. cinerea ActinA* relative to the *Arabidopsis ACT2/7* gene. (D) Leaves from 20 day-old wild-type and mutant plants were pre-incubated 24 h on solvent (control), 30 or 60 µM *N*-isobutyl decanamide containing plates, dipped into a *B. cinerea* inoculum of 5×10^5^ spores/ml, transferred to decanamide-free plates and then incubated. Disease symptoms were scored 3 days post-inoculation, graphical representation of disease rating (upper panel) caused in leaves was determined as percentage of leaves showing no symptoms (white bars), chlorosis (grey bars), necrosis (dark grey bars), or severe tissue maceration (black bars). Data values represent one of two independent experiments that gave similar results, 15 leaves were employed per treatment in each assay.

To test whether JA signaling is involved in the *N*-isobutyl decanamide-induced increased resistance to necrotrophic fungal infection of *Arabidopsis* leaves, we evaluated the responses of *Arabidopsis* JA-related mutants *jasmonic acid resistant1* (*jar1*), *coronatine insensitive1* (*coi1*-*1*), a mutant defective at the *MITOGEN-ACTIVATED PROTEIN KINASE6* (*MPK6*) locus, which has been found to be critical in defense responses to *B. cinerea* [22], and the SA-related mutant *enhanced disease symptoms16* (*eds16*/*sid2-1*). Fully developed leaves from Col-0 wild-type (WT) and mutant plants were pre-incubated for 24 hours with 30 µM *N*-isobutyl decanamide or with the solvent as control and then, inoculated with a droplet of 5×10^5^ spores/ml *B. cinerea* spores on the surface. WT and *eds16* leaves showed decreased disease symptoms when treated with *N*-isobutyl decanamide, unlikely *jar1*, *mpk6* and *coi1-1* leaves, which presented symptoms similar to solvent-treated controls, and, therefore, were not responsive to the alkamide-activated resistance (Figure 7A & B). Similarly, fungal growth, assessed by quantitative PCR amplification of *Actin A* DNA of *B. cinerea* [53], was significantly enhanced in the *jar1*, *mpk6* and *coi1-1* mutant leaves, but restricted in the WT and *eds16* with the *N*-isobutyl decanamide treatment (Figure 7C).

In order to qualitatively analyze the damage caused by the pathogen, infection was estimated by recording a range of severity of disease symptoms (from no symptoms, to severe tissue maceration). For this purpose, we placed leaves from WT and mutant plants on 0.7% agar plates supplied with solvent or *N*-isobutyl decanamide for 24 hours. Then, leaves were immersed into *B. cinerea* spores solution and transferred to *N*-isobutyl decanamide-free agar plates. As shown in Figure 7D, three d.a.i., lesion severity was higher in the *jar1*, *mpk6* and *coi1-1* mutants than in WT leaves, even when they received *N*-isobutyl decanamide pre-treatment. As expected in the WT, disease symptoms decreased when the alkamide was supplied. Unlike JA-related mutants, *eds16/sid2-1* leaves displayed reduced injuries with *N*-isobutyl decanamide pretreatment, as compared to those observed for the corresponding untreated controls (Figure 7D).

All together, these results show that *N*-isobutyl decanamide-induced resistance to *B. cinerea* in *Arabidopsis* WT leaves is due to the induction of defense programs that require an intact JA signaling pathway.

## Discussion

Lipids, besides being important structural molecules in living systems, function as modulators of a multitude of signal transduction pathways evoked by environmental and developmental stimuli. Alkamides belong to a novel class of lipid signals that regulate morphological processes in plants [23]. Recent findings provided evidence of a widespread distribution of structurally related lipid amide signals in evolutionary distant organisms, including the animal, fungal and plant-produced NAEs and the bacterial quorum-sensing AHL regulators [54], [33].

The central idea of this work was to explore the transcriptional responses in *Arabidopsis* to alkamides and ascertain its relevance in activating long-term defense responses. Toward this goal, we performed a global transcription profile of *Arabidopsis* response to *N*-isobutyl decanamide (Figure S1).

Our results establish that exogenous application of *N*-isobutyl decanamide triggers profound physiological changes in *Arabidopsis*, with activation of developmental and defense- and stress-related genes (Table S1; Figures 2 & 3). Interestingly, treatment with *N*-isobutyl decanamide resulted in an increase in the endogenous levels of JA (Figure 5), a lipid phytohormone known to be central in activation of plant defense responses to a range of biotic challengers, including herbivores, insects, necrotrophic fungi and oomycetes [55], [56], [57]. These results are consistent with previous reports showing that NAEs induce LOXs activity and JA accumulation [58], [59]. NAEs comprise a group of bioactive signaling lipids naturally present from fungi to plants to mammals that share structural and functional relationship with alkamides. In mammals, anandamide (NAE 20∶4) acts as an endogenous ligand for cannabinoid receptors and plays different physiological roles including the modulation of neurotransmission in the central nervous system [60], synchronization of embryo development [61] and vasodilation [62]. Interestingly, arachidonic acid (AA, 20∶4) a precursor of anandamide in mammals has been shown to posses important signaling roles in plant stress and defense networks trough production of JA and the activation of JA-dependent transcripts [8]. These results indicate that lipid signals biochemically related to alkamides and NAEs could regulate the same or similar signaling pathways.

Previously, Teaster and coworkers [63] conducted microarray analyses to identify transcriptional targets of plant NAE 12∶0 in 4 d-old *Arabidopsis* seedlings. We found a set of 171 differentially expressed genes by *N*-isobutyl decanamide, whose expression was also reported as regulated by NAEs, including ABA-responsive genes (At3g02480, At5g53820) and germin-like genes (At5g38910, At5g39550, At5g39180, At5g39110, At5g39190) (Table S1). Our results indicate that although important differences in plant age, concentrations of compounds and time of exposure already exists when comparing our expression analysis results with those reported for NAE 12∶0, common genes were found to be up-regulated by the two compounds, indicating commonalities in the transcriptional responses elicited by NAE 12∶0 and *N*-isobutyl decanamide. One of the first indications that plant fatty acid amides indeed participate in plant-pathogen interactions was the observation that NAEs accumulated in the growth media of tobacco suspension cells and leaves after application of the fungal elicitor xylanase. Indeed, exogenous NAE application triggered the expression of the *PHENYLALANINE AMMONIA LYASE* gene (*PAL*), which has been implicated in plant defense against pathogens [64], [27]. Moreover, the ectopic overexpression of FAAH, a NAE-metabolizing enzyme, renders *Arabidopsis* seedlings more susceptible to both host and non-host bacterial pathogens [28], [65]. However, it remains to be determined whether NAE application can actually confer improved resistance of plants to pathogens.

Upon *N*-isobutyl decanamide treatment, several JA-related genes such as *PDFs* (At2g43510, At5g44420, At1g75830, At2g26010), *VSP2* (At5g24770), *JAZ10* and *JAZ8* (At5g13210, At1g30135), were induced, with a maximum at day 7 after alkamide treatment (Figure 4), which correlates with the up-regulation of several genes encoding enzymes involved in JA biosynthesis and with a two-fold increased JA level (Figure 4). Similar long-term gene induction patterns and JA increase have been described in *Medicago truncatula* plants inoculated with the pathogenic soilborne fungus *Phymatotrichopsis omnivora*, which has a very broad host range and infects almost 2,000 dicotyledonous species. Transcriptomic analysis of this interaction provided evidence that JA production is sustained and prolonged, inducing expression of genes encoding for LOXs, AOC2, OPR3, OPR5, OPR12, and wound-inducible serine proteinase inhibitors (PII) at 3 and 5 days after inoculation [66]. We show that *N*-isobutyl decanamide treatment conferred protection against *B. cinerea* attack to *Arabidopsis* leaves (Figure 6). In contrast to wild-type and the SA-related mutant *eds16*/*sid2-1*, all three *jar1*, *coi1* and *mpk6 Arabidopsis* mutants, whose gene products are involved in JA sensitivity and signaling, failed to resist *B. cinerea* attack when *N*-isobutyl decanamide was supplied (Figure 7), suggesting that *N*-isobutyl decanamide-conferred resistance to necrotrophic fungi requires an intact JA signaling pathway.

Responses of plants to necrotrophic pathogens involve multiple intermediates in signal transduction and anti-microbial responses, which includes nitric oxide (NO) and reactive oxygen species (ROS) [67]. The production and accumulation of reactive oxygen species ROS, primarily superoxide (O_2_-) and hydrogen peroxide (H_2_O_2_), during the course of a plant-pathogen interaction has long been recognized. Evidence suggests that the oxidative burst and the cognate redox signaling engaged subsequently, may play a central role in the integration of a diverse array of plant defense responses [68], [69]. One well studied effect of oxidative burst is the induction of hypersensitive response (HR) mechanism, where the tissue at the infection site dies and in turn confines the pathogen growth preventing its spreading [70]. In our microarray analysis, we identified several potential components of the ROS signaling pathway, including scavenging enzymes catalases and ascorbate peroxidases, as well as at least 20 cytochrome P450 genes, including the antifungal gene *CYP71B15/PAD3*, which plays a key role in camalexin production and resistance against necrotrophic pathogens [71] (Figure 4B & Table S1). Activation of these genes correlated with accumulation of hydrogen peroxide (H_2_O_2_) and NO in *N*-isobutyl decanamide-treated leaves (Figure 3). We propose that alkamides might influence plant-pathogen interactions by affecting the level of other lipids or by modulating the levels of second messengers involved in signal transduction to these lipids such as Ca^2+^, NO, and/or ROS. In a developmental context, the relationship between NO and alkamides pathways in *Arabidopsis* was recently investigated, mitotic activation of pericycle cells from seedlings roots induced by *N*-isobutyl decanamide occurred in parallel and in a dependent way to NO synthesis [45]. However, whether NO mediates the defense responses to alkamides remains to be clarified.

Our previous research revealed a genetic interaction of alkamides and senescence responses mediated by the *DRR1* locus in *Arabidopsis* [32]. Leaf senescence is a metabolic active process controlled by a genetic program [72], [73]. Interestingly, ultra-structural changes in senescing cells are accompanied by production of several metabolites that may influence interactions with other organisms. For example, antimicrobial compounds often accumulate in senescing tissues, preventing diseases [74]. In agreement with this, many senescence genes are transcriptionally up-regulated by *N*-isobutyl decanamide, i.e. *PR* genes, *SAG* genes (At2g29350, At4g17670, At5g47060), a member of TCP family (At5g40070), and JA-related genes. It is tempting to speculate that *N*-isobutyl decanamide can be recognized as a senescence-induced signal, thus influencing developmental and defense programs involving JA signaling and possibly other additional signaling/metabolic pathways regulated by NO, ROS and/or MAPK messengers.

*N*-isobutyl decanamide share structural similarity to *N*-decanoyl homoserine lactone (C:10 AHL) [25], a member of the bacterial quorum-sensing signals, which have been found to alter root development and activate defense responses in different plant species [30], [29], [25]. An interesting hypothesis is that small lipid signaling based on plant fatty acid amides and/or AHLs might be part of an ancestral inter-kingdom communication system between plants and their associated bacteria. Our data thus expand the repertoire of signaling lipid molecules known to trigger plant defenses and provide evidence that alkamides interact with the JA pathway. The use of alkamides and bacterially produced fatty amides in pathogen resistance by acting as defense elicitors in plants shows great potential towards application of these compounds to combat pathogen pests.

## Materials and Methods

### Plant material and growth conditions

*Arabidopsis thaliana* ecotype Col-0 was used for all experiments unless indicated otherwise. Col-0, transgenic *LOX2∶GUS* [75] and *PR1∶GUS*, and mutants *jar1* [16], *mpk6* [76], *coi1-1* [77] and *eds16/sid2-1* [78] seeds were surface sterilized with 95% (v/v) ethanol for 5 min and 20% (v/v) bleach for 7 min. After five washes in distilled water, seeds were germinated and grown on agar plates containing 0.2× MS medium. Plates were placed vertically at an angle of 65° to allow root growth along the agar surface and to allow unimpeded growth of the hypocotyl into the air. For plant growth, we used a plant growth cabinet (Percival Scientific AR95L, Perry, IA), with a photoperiod of 16 h of light, 8 h of darkness, light intensity of 300 µmol/m-^2^/s-^1^ and temperature of 22°C. After grown for 6 days, plants were transferred to control or *N*-isobutyl decanamide containing solid MS medium for different times.

Homozygous *coi1-1* seedlings were selected by screening a heterozygous population in agar solidified MS medium supplied with 5 µM JA (Sigma Chemical Co., St. Louis), seedlings resistant to root inhibition were transferred to soil baskets and leaves from 20 d-old were detached for *in vitro* pathogenicity assays.

### Synthesis of *N*-isobutyl decanamide

*N*-isobutyl-decanamide was obtained by catalytic reduction of affinin, the most abundant alkamide present in *Heliopsis longipes* (Gray) Blake (Asteraceae) roots as described before [23].

### Experimental design and microarray platform

For microarray analyses a dye balanced modified loop design was implemented. Four biological replicates representing each sampling point were obtained by pooling in a 1∶1 proportion shoot and root purified RNA from 120 randomly chosen seedlings. This experiment involved a total of sixteen sets of microarray hybridizations, including direct and dye swap comparisons between treatments as well as across time points for the same treatment. This design allowed us to determine differences in gene expression between *N*-isobutyl decanamide-treated and control seedlings, and whether the differences were time dependent. The *Arabidopsis* Oligonucleotide Array version 3.0 from The Arizona University was used to carry out this study. Array annotation and composition are available at http://ag.arizona.edu/microarray. RNA isolation, fluorescent labeling of probes, slide hybridization and washing were performed as described previously in [79]. Slides were scanned with an Axon GenePix 4100 scanner at a resolution of 10 µm adjusting the laser and gain parameters to obtain similar levels of fluorescence intensity in both channels. Spot intensities were quantified using Axon GenePix Pro 5.1 image analysis software.

All microarray data is MIAME compliant and the raw data has been deposited in the Gene Expression Omnibus database (GEO), accession number GSE12107, as detailed on the MGED Society website http://www.mged.org/Workgroups/MIAME/miame.html.

### Normalization and data analysis

Raw data were imported into the R 2.2.1 software (http://www.R-project.org). Background correction was done using the method “substract” whereas normalization of the signal intensities within slides was carried out using the “printtiploess” method [80] using the LIMMA package (www.bioconductor.org). Normalized data were log2 transformed and then fitted into mixed model ANOVAs [81] using the Mixed procedure (SAS 9.0 software, SAS Institute Inc., Cary, NC, USA) with two sequenced linear models considering as fixed effects the dye, time, *N*-isobutyl-decanamide treatment and time × *N*-isobutyl-decanamide treatment. Array and array × dye were considered as random effects. The type 3 F-tests and p-values of the time × *N*-isobutyl-decanamide treatment and *N*-isobutyl-decanamide treatment were also carried out. Model terms were explored and significance levels for those terms were adjusted for by the False Discovery Rate (FDR) method [82]. Estimates of the expression differences were calculated using the mixed model. Based on these statistical analyses, the spots with tests with an FDR less or equal to 5% and with changes in signal intensity between *N*-isobutyl decanamide treatment and control seedlings of 2.0-fold or higher were considered as differentially expressed.

### Expression analysis by qRT-PCR

Total RNAs were isolated from *Arabidopsis* plants using TRIzol reagent (Invitrogen). Primer design (Tm, 60–65°C) was performed using Primer Express Software, Version 3 (Applied Biosystems); full sequences from each primer are shown in Table S3. cDNA templates for PCR amplification were prepared from all samples by using reverse specific primers and SuperScript III reverse transcriptase (Invitrogen) according to the manufacturer's instructions. Each reaction contained cDNA template from ∼30 µg total RNA, 1× SYBR Green PCR Master Mix (Applied Biosystems) and 500 nM forward and reverse primers. Real-time PCR was performed in an ABI PRISM 7500 sequence detection system (Applied Biosystems) under the following thermal cycling conditions: 10 min at 95°C followed by a total of 40 cycles of 30 s at 95°C, 30 s at 60°C and 40 s at 72°C. For qRT-PCR, relative transcript abundance was calculated and normalized with respect to *ACTIN2/7* to minimize variation in cDNA template levels, with the solvent-treated (control) and control Col-0 samples acting as calibrators (for microarrays validation assay for and JA responsive genes assay respectively). Data shown represent mean values obtained from at least three independent amplification reactions; the SE of the C_T_s averaged 0.1, demonstrating the high precision of the assays. All calculations and analyses were performed using 7500 Software v2.0.1 (Applied biosystems) and the 2^−ΔΔCT^ method [83]. Amplification efficiency for the primer sets was determined by amplification of cDNA dilution series (1∶5). The values obtained not change significantly between different cDNA smaples, and were always higher than 0.90. Specificity of the RT-PCR products was followed by a melting curve analysis with continual fluorescence data acquisition during the 65–95°C melt.

### Analysis of JA levels

250 mg of freshly harvested plant tissues were chilled in liquid nitrogen and JA extraction was performed as in [84] using dihydrojasmonate as internal standar, derivatized with chloroform/*N,N*-diisoprpyl-etylamine 1∶1. In order to analyze the samples by GC/MS the extract was added with 10 µl of PFBr and 200 µl of cloroform: *N,N*-diisopropilethilamine (1∶1) then incubated at 65°C for 1 h. When cooled, the solvent was evaporated to dryness and resuspended in 100 µl methanol. Samples were analyzed in a gas chromatograph (Agilent Technologies 7890A) equipped with a capillary column J&W DB-1 (60 m×250 µm×0.25 µm) coupled to a mass selective detector (Agilent 5973 Series MSD). Using an autosampler 7683B Series. 2 µl of the sample was injected in a splitless way. Operating conditions were: injector temperature 250°C; the oven temperature was programmed as: initial temperature 150°C for 3 min then increasing at the rate of 4°C per min to a final temperature of 280°C maintained for 20 min. Helium was used as carrier gas with a constant flow of 1 ml/min. The MS was set to scan from 40 to 600 uam in Synchronous SIM/Scan mode for selectively monitor the following ions for jasmonic acid derivative: 141, 181, 390, and 392. MS temperatures were: Source 230°C, MS Quadrupole 150°C.

### Microscope Analyzes

For histochemical analysis of transgenic lines *LOX2∶GUS* and *PR1∶GUS*, 7 d-old transgenic seedlings expressing these marker constructs were incubated at 37 °C in a GUS reaction buffer (0.5 mg/ml of 5-bromo-4-chloro-3-indolyl-B-D-glucuronide in 100 mM sodium phosphate, pH 7.0). The stained seedlings were cleared by the method of Malamy and Benfey [85]. For each treatment, at least 9 transgenic plants were analyzed. A representative plant was chosen for each treatment and photographed using the Nomarski optics on a Leica DMR microscope.

H_2_O_2_ production was detected by the endogenous peroxidase-dependent staining procedure using 3,3-diaminobenzidine (DAB) uptake [86]. Control, 15 and 30 µM *N*-isobutyl decanamide-treated 7 d seedlings were placed in a solution of 1 mg mL^−1^ DAB, pH 3.8, and incubated in dark for 2 h. Subsequently, were immersed in boiling 96% (v/v) ethanol for 10 min and then stored in 96% (v/v) ethanol. For each treatment, at least 9 treated-seedlings were analyzed. A representative plant was chosen for each treatment. H_2_O_2_ production was visualized as a reddish-brown precipitated coloration and photographed using the Nomarski optics on a Leica DMR microscope.

Nitric Oxide (NO) was monitored by incubating *Arabidopsis* seedlings with 10 µM of the fluorescent probe 4,5-diaminofluorescein diacetate (DAF-2DA) [87] in 0.1 M Tris–HCl (pH 7.4). Treated seedlings were incubated for 2 h in the dark, and washed three times for 20 min with fresh buffer. Fluorescence signals from at least 9 treated and control leaves were detected using a confocal laser scanning microscope (model BX50, Olympus), and monitored with an argon blue laser with an excitation line from 488 to 568 nm and an emission window from 585 to 610 nm.

### Fungal growth and plant inoculation

Pathogenesis assays were modified from [88]. *Botrytis cinerea* was grown on agar PDA medium (PhytoTechnology) for 7–12 days at 22° C in darkness. Spores were collected with distilled water. Col-0 superficially sterilized seeds were germinated and grown in MS-agar medium into 100 ml flasks with transparent lid. At 20 days after germination, rosette leaves were placed in Petri dishes with 60 µM of *N*-isobutyl decanamide containing medium or medium supplied with the solvent. Inoculation was performed by placing a 5 µl drop of a suspension of 5×10^5^ conidiospores/ml on the surface of leaves. The samples were incubated at 22°C and analyzed at a further 3, 4 and 5 d period after inoculation. Susceptibility was evaluated by microscopic observation of necrotic symptoms under a dissecting microscope (Leica MZ6) connected to a digital color camera (Samsung SCC-131A). The percent of necrotic leaves was scored for 30 independent inoculated leaves. The disease symptoms on inoculated leaves and fungal growth over leaves was estimated by trypan blue staining and further cleared with chloral hydrate and the extension of necrotic lesions (lesion diameter) measured at 4 d after inoculation. For mutant inoculation, leaves from soil grown adult plants were incubated in agar solution supplied with solvent (ethanol) or *N*-isobutyl decanamide during 24 h prior to inoculation by leaves immersion into solution of 5×10^5^ conidiospores/ml.

## Supporting Information

Figure S1 **Experimental design for microarray analysis.** 6 day-old *Arabidopsis* Col-0 seedlings were grown on *N*-isobutyl decanamide-free medium and then transferred to control medium (A) supplied with the solvent, or to 60 µM *N*-isobutyl decanamide-containing medium (B). Pictures were taken 14 days after transfer (d.a.t.). Modified loop design including 4 independent replicates evaluated at 1, 3, 7, and 14 d.a.t. (C). A total of 16 slides were employed. Each replicate was conformed by at least 120 transferred seedlings, which were harvested from four independent plates.
(TIF)

Figure S2 **Validation of microarray results via qRT-PCR.** Quantitative real-time PCR analysis was performed for 15 genes in *Arabidopsis* (Col-0) seedlings, under the same conditions used for microarray analysis (1, 3, 7 and 14 days of treatment with 60 µM *N*-isobutyl decanamide). Fold-change (control to *N*-isobutyl-decanamide) expression for the indicated selected genes in a log2 scale is shown. Expression ratios obtained by microarray experiments (A). Estimates of the differences of expression levels were calculated using the mixed model as described in methods. Expression ratios obtained by qRT-PCR (B). RQ (relative quantification number) was obtained from the equation 2^ΔΔC^_T_ where ΔΔC_T_ represents ΔC_T_(control) - ΔC_T_(*N*-isobutyl decanamide 60 µM). Each C_T_ was previously normalized using the expression levels of *ACT2/7* as internal reference. Expression levels were obtained from four independent replicates, every set of oligonucleotides had an efficiency greater than 99%. Standar deviations were less than 0.1 arbitrary units.
(TIF)

Figure S3 **Local induction of defense genes by** ***N*** **-isobutyl decanamide on detached leaves.** Leaves from 20 day-old transgenic *LOX2∶GUS* or WT (Col-0) plants grown in soil were detached and incubated 24 h on solvent (control, white squares), or 30 µM *N*-isobutyl decanamide containing plates (black squares), transferred to decanamide-free plates and then analized. (A) Dose-response assay with leaves from transgenic *Arabidopsis* line carrying *LOX2∶GUS* were stained for *GUS* expression 24 h after transference to agar plates. (B) qRT-PCR analysis of the JA-responsive genes *OPR3* and *VSP2*, and the camalexin biosynthetic marker *PAD3* using C_T_ value of *ACT2/7* as internal expression reference. Relative expression values were normalized with endogenous levels from each transcript in Col-0 control seedlings. Bars represent ± SE from three independent biological replicates from 30 leaves each one, and from four technical replicates for the assay.
(TIF)

Figure S4 **Effect of** ***N*** **-isobutyl decanamide on** ***Botrytis cinerea*** **mycelial growth.** *B. cinerea* mycelium excised from a solid culture in Petri dishes was transferred to potato dextrose agar dishes supplemented with *N*-isobutyl decanamide at the concentrations indicated. Radial growth of the fungus was measured 24, 48 and 72 h after inoculation (A). Data means average radial growth from three independent samples ± SD; no statistical differences were found at any concentration tested. Mycelial growth at 72 h after inoculation on solvent-containing media (Control) and 120 µM *N*-isobutyl decanamide-supplied media (B). Fungicide Techto 60 was employed at 1 mg/ml as fungal growth inhibition control to compare with the highest concentration of *N*-isobutyl decanamide in divided Petri dishes (C).
(TIF)

Table S1 **Full list of genes differentially expressed by** ***N*** **-isobutyl decanamide in** ***Arabidopsis*** **.**
(PDF)

Table S2 **Functional categories over-represented in** ***N*** **-isobutyl decanamide responsive genes.**
(PDF)

Table S3 **Sequences of oligonucleotides used as PCR primers for quantitative expression analysis.**
(PDF)
