# Supplementary figures and images for: Alkamides Activate Jasmonic Acid Biosynthesis and Signaling Pathways and Confer Resistance to Botrytis cinerea in Arabidopsis thaliana
Source: PLoS One. 2011 Nov 4;6(11):e27251. doi: 10.1371/journal.pone.0027251 (PMC3208606; doi:10.1371/journal.pone.0027251)

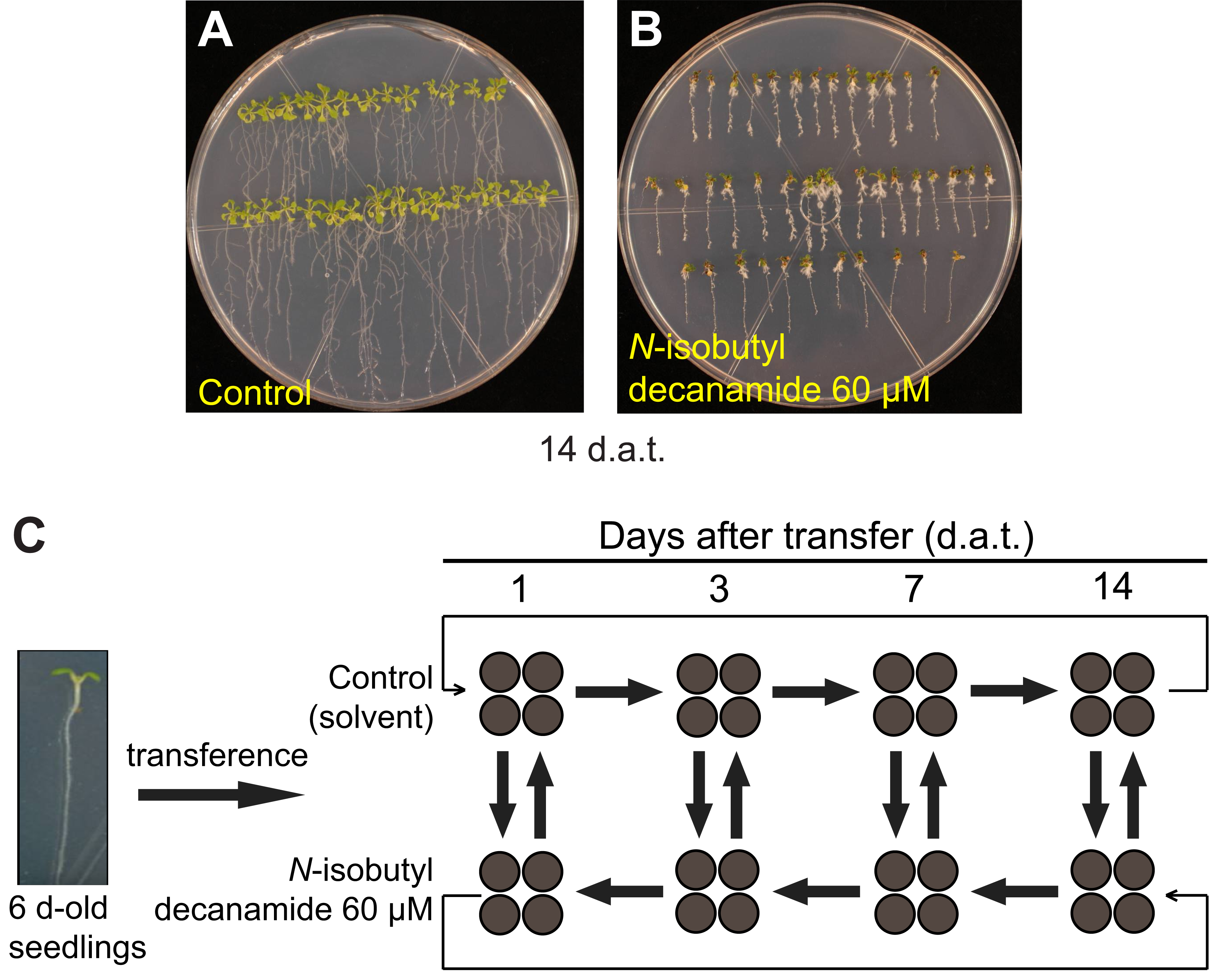

Supplement: Figure S1 — Experimental design for microarray analysis. 6 day-old Arabidopsis Col-0 seedlings were grown on N-isobutyl decanamide-free medium and then transferred to control medium (A) supplied with the solvent, or to 60 µM N-isobutyl decanamide-containing medium (B). Pictures were taken 14 days after transfer (d.a.t.). Modified loop design including 4 independent replicates evaluated at 1, 3, 7, and 14 d.a.t. (C). A total of 16 slides were employed. Each replicate was conformed by at least 120 transferred seedlings, which were harvested from four independent plates. (TIF) [file pone.0027251.s001.tif]

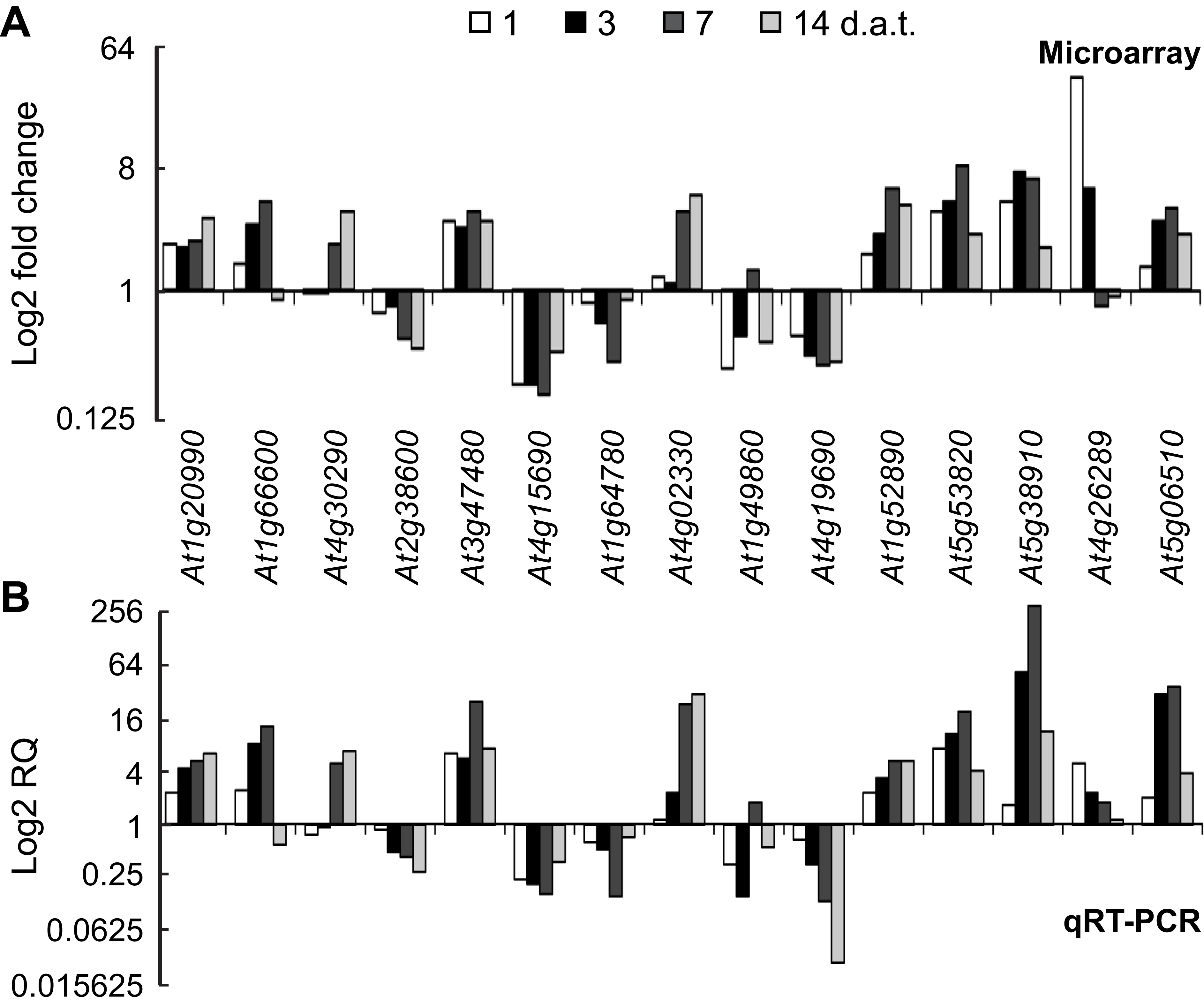

Supplement: Figure S2 — Validation of microarray results via qRT-PCR. Quantitative real-time PCR analysis was performed for 15 genes in Arabidopsis (Col-0) seedlings, under the same conditions used for microarray analysis (1, 3, 7 and 14 days of treatment with 60 µM N-isobutyl decanamide). Fold-change (control to N-isobutyl-decanamide) expression for the indicated selected genes in a log2 scale is shown. Expression ratios obtained by microarray experiments (A). Estimates of the differences of expression levels were calculated using the mixed model as described in methods. Expression ratios obtained by qRT-PCR (B). RQ (relative quantification number) was obtained from the equation 2ΔΔC T where ΔΔCT represents ΔCT(control) - ΔCT(N-isobutyl decanamide 60 µM). Each CT was previously normalized using the expression levels of ACT2/7 as internal reference. Expression levels were obtained from four independent replicates, every set of oligonucleotides had an efficiency greater than 99%. Standar deviations were less than 0.1 arbitrary units. (TIF) [file pone.0027251.s002.tif]

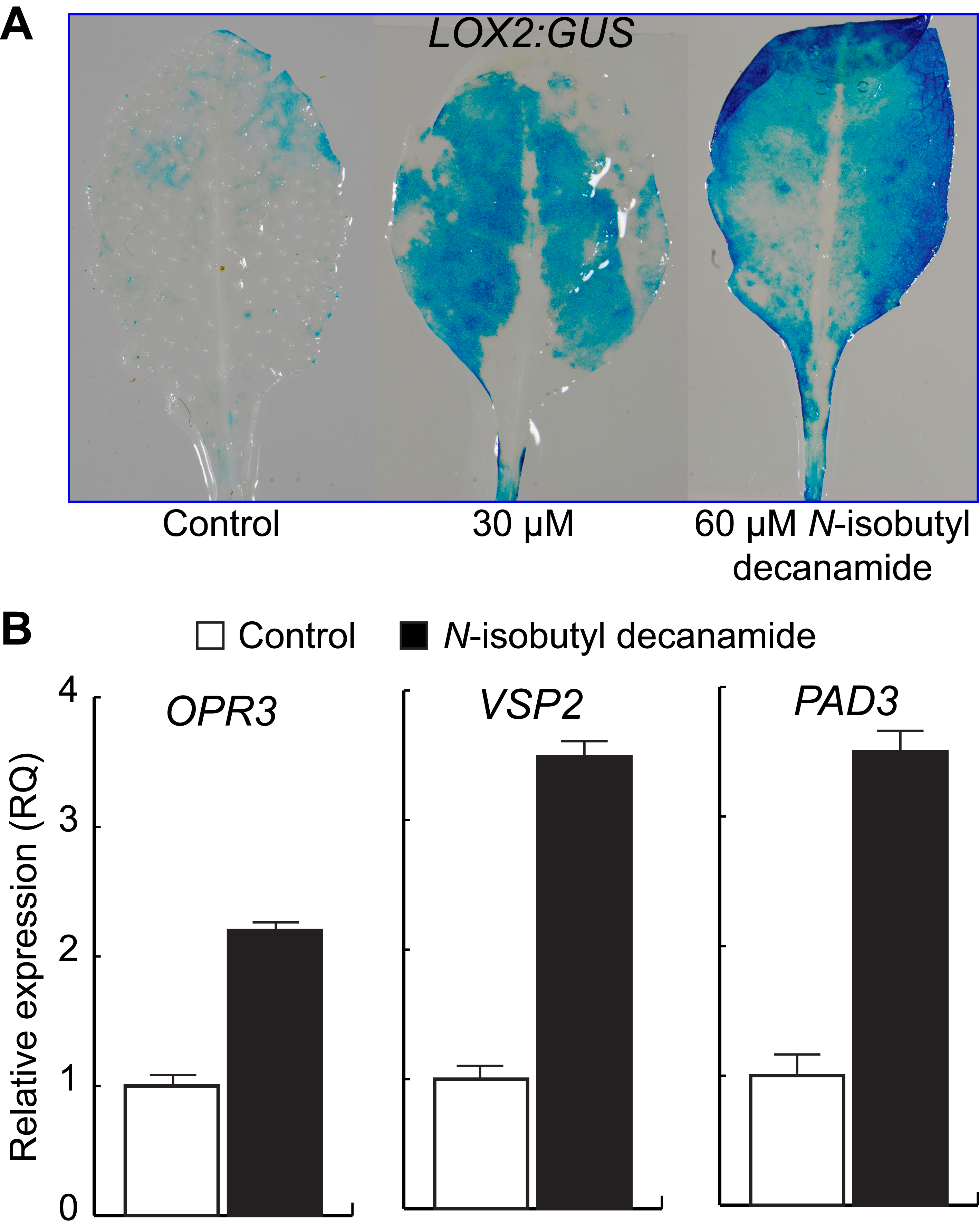

Supplement: Figure S3 — Local induction of defense genes by N -isobutyl decanamide on detached leaves. Leaves from 20 day-old transgenic LOX2∶GUS or WT (Col-0) plants grown in soil were detached and incubated 24 h on solvent (control, white squares), or 30 µM N-isobutyl decanamide containing plates (black squares), transferred to decanamide-free plates and then analized. (A) Dose-response assay with leaves from transgenic Arabidopsis line carrying LOX2∶GUS were stained for GUS expression 24 h after transference to agar plates. (B) qRT-PCR analysis of the JA-responsive genes OPR3 and VSP2, and the camalexin biosynthetic marker PAD3 using CT value of ACT2/7 as internal expression reference. Relative expression values were normalized with endogenous levels from each transcript in Col-0 control seedlings. Bars represent ± SE from three independent biological replicates from 30 leaves each one, and from four technical replicates for the assay. (TIF) [file pone.0027251.s003.tif]

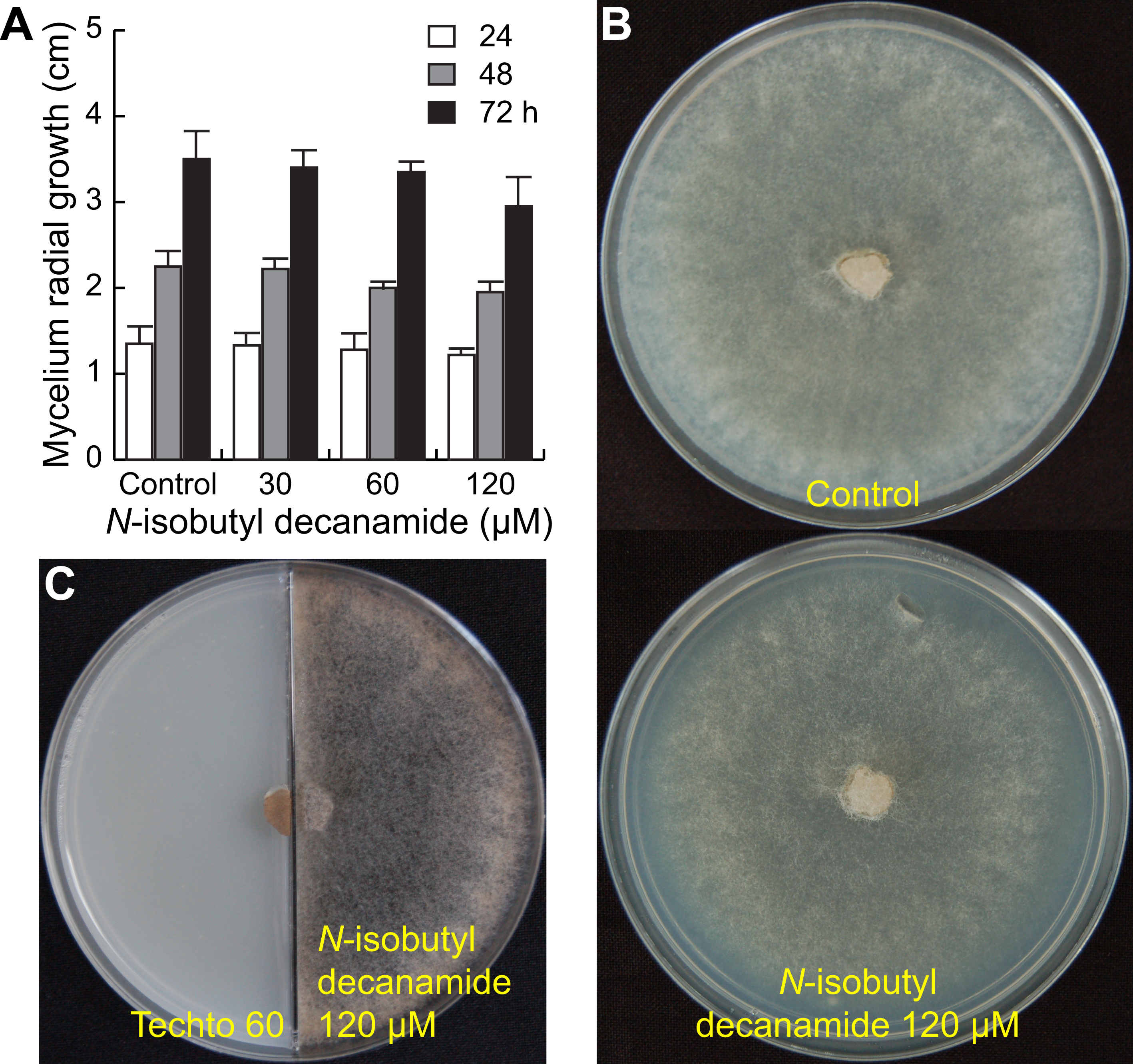

Supplement: Figure S4 — Effect of N -isobutyl decanamide on Botrytis cinerea mycelial growth. B. cinerea mycelium excised from a solid culture in Petri dishes was transferred to potato dextrose agar dishes supplemented with N-isobutyl decanamide at the concentrations indicated. Radial growth of the fungus was measured 24, 48 and 72 h after inoculation (A). Data means average radial growth from three independent samples ± SD; no statistical differences were found at any concentration tested. Mycelial growth at 72 h after inoculation on solvent-containing media (Control) and 120 µM N-isobutyl decanamide-supplied media (B). Fungicide Techto 60 was employed at 1 mg/ml as fungal growth inhibition control to compare with the highest concentration of N-isobutyl decanamide in divided Petri dishes (C). (TIF) [file pone.0027251.s004.tif]
