# Supplementary material for: Alkamides Activate Jasmonic Acid Biosynthesis and Signaling Pathways and Confer Resistance to Botrytis cinerea in Arabidopsis thaliana
Source: PLoS One. 2011 Nov 4;6(11):e27251. doi: 10.1371/journal.pone.0027251 (PMC3208606; doi:10.1371/journal.pone.0027251)
Supplement: Table S1 — Full list of genes differentially expressed by N -isobutyl decanamide in Arabidopsis . (PDF) [file pone.0027251.s005.pdf]

Table S1. Differentially expressed genes by *N* -Isobutyl decanamide in *Arabidopsis* . Genes were selected according to the stringency levels (FDR ≤0.05 and Fold ±2) in at least one of the four sampled time points, 1, 3, 7 and 14 days after transference (d.a.t.). Each gene is represented by a single row of colored boxes and each sampled time points, is represented by a single column. Color scale ranges are shown as intensities of blue and red colors (down-regulated and up-regulated, respecively), indicating the range from fold change values.

	Locus	Description	FDR D*T	FDR T	FoldChange (d.a.t.)			
					1	3	7	14
1	At3g21340	leucine-rich repeat protein kinase, putative	>0.05	0.003	0.346	0.197	0.484	0.563
2	At4g16410	similar to hypothetical protein [Vitis vinifera] (GB:CAN7	0.037	0.022	1.164	0.736	0.484	0.855
3	At5g50915	basic helix-loop-helix (bHLH) family protein	>0.05	0.031	0.987	0.793	0.344	0.808
4	At1g42550	PMI1 (PLASTID MOVEMENT IMPAIRED1)	0.047	0.026	1.018	0.828	0.499	0.903
5	At3g21870	CYCP2;1 (cyclin p2;1); cyclin-dependent protein kinase	0.020	0.008	1.208	0.707	0.282	0.724
6	At4g26530	fructose-bisphosphate aldolase, putative	0.020	0.008	1.170	0.747	0.417	0.796
7	At5g20630	GLP3 (GERMIN-LIKE PROTEIN 3); manganese ion bindir	>0.05	0.024	1.007	0.795	0.338	0.672
8	At5g22020	strictosidine synthase family protein	0.035	0.004	0.967	0.818	0.434	0.636
9	At2g18560	UDP-glucoronosyl/UDP-glucosyl transferase family pro	0.021	0.002	0.922	0.829	0.433	0.690
10	At2g29130	LAC2 (laccase 2); copper ion binding / oxidoreductase	>0.05	0.010	1.030	0.715	0.394	0.689
11	At3g05730	similar to hormone [Arabidopsis thaliana] (TAIR:AT3G0	>0.05	0.031	1.080	0.649	0.423	0.709
12	At5g35630	GS2 (GLUTAMINE SYNTHETASE 2); glutamate-ammonia	0.031	0.014	0.920	0.667	0.425	1.073
13	At5g38420	ribulose bisphosphate carboxylase small chain 2B / RuI	0.039	0.020	0.929	0.668	0.374	1.034
14	At5g03555	permease, cytosine/purines, uracil, thiamine, allantoin	0.049	0.019	0.946	0.737	0.461	0.973
15	At3g22210	unknown protein	0.028	0.005	0.925	0.731	0.456	0.877
16	At4g25700	BETA-OHASE 1 (BETA-HYDROXYLASE 1); beta-carotene	0.038	0.013	1.030	0.695	0.443	0.899
17	At4g28080	binding	0.032	0.006	1.018	0.668	0.445	0.840
18	At5g62210	embryo-specific protein-related	>0.05	0.030	0.933	0.804	0.495	0.978
19	At3g11110	zinc finger (C3HC4-type RING finger) family protein	0.050	0.027	0.927	0.743	0.335	0.945
20	At5g04150	BHLH101; DNA binding / transcription factor	0.037	0.019	0.827	0.773	0.187	0.856
21	At1g50730	similar to unnamed protein product [Vitis vinifera] (GB	0.012	0.017	1.290	0.758	0.399	0.972
22	At1g14150	oxygen evolving enhancer 3 (PsbQ) family protein	0.008	>0.05	1.292	0.798	0.351	0.992
23	At1g68010	HPR (HYDROXYPYRUVATE REDUCTASE); glycerate dehy	0.021	0.020	1.052	0.744	0.457	1.063
24	At3g01500	CA1 (CARBONIC ANHYDRASE 1); carbonate dehydratas	0.035	>0.05	1.143	0.731	0.443	1.095
25	At1g68110	epsin N-terminal homology (ENTH) domain-containing	0.020	>0.05	1.248	0.746	0.424	1.108
26	At1g19150	LHCA6 (Photosystem I light harvesting complex gene 6)	0.032	0.048	1.230	0.689	0.458	0.987
27	At5g05270	chalcone-flavanone isomerase family protein	0.009	0.033	1.144	1.287	0.298	0.873
28	At3g55120	A11/CFI/TT5 (TRANSPARENT TESTA 5); chalcone isome	0.004	0.005	1.111	1.239	0.333	0.783
29	At5g08640	FLS (FLAVONOL SYNTHASE)	0.009	0.010	1.067	1.179	0.243	0.792
30	At3g51240	F3H (TRANSPARENT TESTA 6); naringenin 3-dioxygenas	0.009	>0.05	1.472	1.160	0.322	0.822
31	At3g55630	ATDFD (A. THALIANA DHFS-FPGS HOMOLOG D); tetrah	0.013	0.024	1.197	1.080	0.356	0.790
32	At4g25780	pathogenesis-related protein, putative	0.016	>0.05	1.197	1.085	0.447	0.880
33	At5g05250	similar to unknown protein [Arabidopsis thaliana] (TAII	0.035	>0.05	1.265	1.072	0.331	0.876
34	At1g78380	ATGSTU19 (GLUTATHIONE TRANSFERASE 8); glutathior	0.020	>0.05	1.304	1.136	0.482	0.772
35	At2g22590	glycosyltransferase family protein	0.012	0.022	1.068	1.279	0.410	0.733
36	At4g15480	UGT84A1; UDP-glycosyltransferase/ sinapate 1-glucosy	0.012	>0.05	1.068	1.555	0.273	0.770
37	At4g01915	unknown protein	0.009	>0.05	1.141	1.392	0.450	0.874
38	At5g13930	ATCHS/CHS/TT4 (CHALCONE SYNTHASE); naringenin-ch	0.008	0.002	1.067	1.067	0.330	0.599
39	At1g48300	similar to hypothetical protein [Vitis vinifera] (GB:CAN8	0.017	0.005	1.029	0.993	0.404	0.663
40	At5g16530	PIN5 (PIN-FORMED 5); auxin:hydrogen symporter/ trar	0.015	0.005	0.965	1.009	0.363	0.675
41	At5g43630	zinc knuckle (CCHC-type) family protein	0.021	0.011	0.815	1.166	0.363	0.706

42	At1g32375	F-box family protein	0.048	0.044	0.938	1.132	0.432	0.703
43	At1g50290	unknown protein	0.013	0.012	1.007	0.973	0.417	0.954
44	At1g72645	unknown protein	0.029	0.044	0.947	0.994	0.321	0.939
45	At4g27410	RD26 (RESPONSIVE TO DESSICATION 26); transcription	0.047	>0.05	0.858	1.023	0.452	0.981
46	At1g65060	4CL3 (4-coumarate:CoA ligase 3); 4-coumarate-CoA liga	>0.05	0.038	0.958	0.870	0.328	0.794
47	At1g80440	kelch repeat-containing F-box family protein	0.040	0.026	0.986	0.959	0.489	0.834
48	At4g37760	SQE3 (SQUALENE EPOXIDASE 3); oxidoreductase	0.013	0.006	1.073	0.925	0.462	0.850
49	At1g79270	ECT8 (evolutionarily conserved C-terminal region 8)	0.028	0.016	0.927	1.018	0.481	0.829
50	At5g21430	DNAJ heat shock N-terminal domain-containing protein	0.026	>0.05	1.341	0.951	0.484	1.046
51	At1g66130	oxidoreductase N-terminal domain-containing protein	0.042	>0.05	1.283	0.923	0.497	1.066
52	At2g05620	PGR5 (PROTON GRADIENT REGULATION 5)	0.018	>0.05	1.219	0.952	0.499	1.138
53	At5g38510	rhomboid family protein	0.015	>0.05	1.351	1.016	0.466	1.098
54	At3g47430	PEX11B	0.020	>0.05	1.556	1.054	0.417	1.074
55	At1g22630	heat shock protein binding / unfolded protein binding	0.021	>0.05	1.305	1.013	0.463	1.234
56	At5g25130	CYP71B12 (cytochrome P450, family 71, subfamily B, p	0.019	>0.05	1.326	0.947	0.258	1.085
57	At1g18730	similar to unknown [Populus trichocarpa] (GB:ABK9526	0.043	>0.05	1.140	0.956	0.492	1.042
58	At3g15354	SPA3 (SPA1-RELATED 3); signal transducer	0.013	>0.05	1.098	0.998	0.392	1.091
59	At3g56290	similar to hypothetical protein [Vitis vinifera] (GB:CAN7	0.012	0.019	1.251	0.959	0.252	0.884
60	At5g37260	CIR1/RVE2 (CIRCADIAN 1); DNA binding / transcription	0.020	>0.05	1.687	0.922	0.288	0.698
61	At2g38230	ATPDX1.1 (PYRIDOXINE BIOSYNTHESIS 1.1); protein het	0.020	>0.05	1.374	0.874	0.402	0.863
62	At4g25910	NFU3 (NFU domain protein 3)	0.020	>0.05	1.297	0.895	0.484	0.915
63	At1g14200	zinc finger (C3HC4-type RING finger) family protein	0.031	>0.05	1.373	0.856	0.425	0.842
64	At4g12830	hydrolase, alpha/beta fold family protein	0.013	0.045	1.434	0.916	0.332	0.915
65	At3g02830	ZFN1 (ZINC FINGER PROTEIN 1); nucleic acid binding	0.036	>0.05	1.159	0.897	0.495	1.205
66	At4g33666	unknown protein	0.038	>0.05	1.020	0.963	0.477	1.218
67	At5g10140	FLC (FLOWERING LOCUS C); transcription factor	0.013	>0.05	1.481	1.876	0.451	0.861
68	At2g23910	cinnamoyl-CoA reductase-related	0.009	>0.05	1.419	1.569	0.439	0.885
69	At5g48880	KAT5/PKT1/PKT2 (PEROXISOMAL 3-KETO-ACYL-COA TH	0.006	>0.05	1.378	1.379	0.462	1.033
70	At1g47400	similar to unknown protein [Arabidopsis thaliana] (TAII	0.033	>0.05	1.522	1.535	0.420	1.133
71	At3g24500	ATMBF1C/MBF1C (MULTIPROTEIN BRIDGING FACTOR 1	0.025	>0.05	1.361	1.644	0.446	1.055
72	At5g37550	similar to unknown protein [Arabidopsis thaliana] (TAII	0.017	>0.05	1.119	1.880	0.383	0.974
73	At5g39210	CRR7 (CHLORORESPIRATORY REDUCTION 7)	0.030	>0.05	1.585	1.052	0.491	0.888
74	At5g55570	heat shock protein binding / unfolded protein binding	0.025	>0.05	1.714	1.080	0.493	0.886
75	At1g07400	17.8 kDa class I heat shock protein (HSP17.8-CI)	0.032	>0.05	1.768	1.274	0.467	0.911
76	At3g01440	oxygen evolving enhancer 3 (PsbQ) family protein	0.020	>0.05	1.867	1.204	0.462	0.790
77	At5g58770	dehydrodolichyl diphosphate synthase, putative / DED	0.019	>0.05	1.542	1.372	0.404	1.330
78	At2g32290	BAM6/BMY5 (BETA-AMYLASE 6); beta-amylase	0.019	>0.05	1.628	1.338	0.413	1.378
79	At2g36790	UGT73C6 (UDP-GLUCOSYL TRANSFERASE 73C6); UDP-g	0.037	>0.05	2.231	1.013	0.367	1.519
80	At5g37640	UBQ9 (ubiquitin 9)	0.039	>0.05	0.885	1.410	0.484	0.759
81	At5g04000	similar to hypothetical protein [Vitis vinifera] (GB:CAN7	0.016	>0.05	1.305	1.491	0.430	0.578
82	At4g23496	SP1L5 (SPIRAL1-LIKE5)	0.008	0.009	0.972	1.466	0.593	0.482
83	At1g73120	similar to hypothetical protein [Vitis vinifera] (GB:CAN6	0.018	>0.05	1.325	1.378	0.376	2.427
84	At5g05500	pollen Ole e 1 allergen and extensin family protein	0.033	0.008	0.388	0.405	0.716	1.239
85	At2g25980	jacalin lectin family protein	0.039	0.009	0.327	0.356	0.722	1.236
86	At1g54000	myrosinase-associated protein, putative	>0.05	0.014	0.381	0.373	0.688	1.124
87	At1g43160	RAP2.6 (related to AP2 6); DNA binding / transcription	>0.05	0.016	0.402	0.373	0.791	1.101
88	At5g28510	glycosyl hydrolase family 1 protein	0.045	0.009	0.412	0.489	0.800	1.072
89	At4g33790	acyl CoA reductase, putative	>0.05	0.008	0.141	0.189	0.577	1.042
90	At5g58010	basic helix-loop-helix (bHLH) family protein	>0.05	0.006	0.355	0.430	0.690	1.055

91	At1g67330	similar to unknown protein [Arabidopsis thaliana] (TAII	>0.05	0.024	0.460	0.566	0.774	1.131
92	At5g06640	proline-rich extensin-like family protein	>0.05	0.006	0.311	0.304	0.629	0.841
93	At4g08410	proline-rich extensin-like family protein	>0.05	0.019	0.450	0.459	0.749	0.909
94	At2g41810	similar to unknown protein [Arabidopsis thaliana] (TAII	>0.05	0.021	0.399	0.377	0.721	0.908
95	At3g54580	proline-rich extensin-like family protein	>0.05	0.006	0.353	0.332	0.618	0.908
96	At5g42510	disease resistance-responsive family protein	>0.05	0.016	0.512	0.443	0.779	0.883
97	At2g24980	proline-rich extensin-like family protein	>0.05	0.015	0.382	0.353	0.718	0.830
98	At1g05240	peroxidase, putative	0.040	0.002	0.236	0.185	0.535	0.708
99	At1g05250	peroxidase, putative	>0.05	0.003	0.281	0.202	0.566	0.738
100	At3g06460	GNS1/SUR4 membrane family protein	>0.05	0.007	0.452	0.365	0.648	0.824
101	At1g50520	CYP705A27 (cytochrome P450, family 705, subfamily A	>0.05	0.004	0.436	0.485	0.698	0.789
102	At2g34180	CIPK13 (SNF1-RELATED PROTEIN KINASE 3.7); kinase	>0.05	0.012	0.480	0.485	0.761	0.832
103	At1g11740	ankyrin repeat family protein	>0.05	0.038	0.313	0.412	0.670	0.844
104	At3g54590	ATHRGP1; structural constituent of cell wall	>0.05	0.010	0.347	0.392	0.652	0.866
105	At5g51520	invertase/pectin methylesterase inhibitor family protei	>0.05	0.013	0.453	0.425	0.975	0.826
106	At2g43220	DC1 domain-containing protein	>0.05	0.016	0.368	0.321	0.893	0.765
107	At5g65690	PCK2/PEPCK (PHOSPHOENOLPYRUVATE CARBOXYKINA	>0.05	0.011	0.203	0.218	0.832	0.653
108	At1g52250	dynein light chain type 1 family protein	>0.05	0.008	0.462	0.449	0.838	0.769
109	At3g16240	DELTA-TIP (delta tonoplast integral protein); water cha	0.049	0.003	0.420	0.375	0.843	0.770
110	At4g20460	NAD-dependent epimerase/dehydratase family proteir	>0.05	0.019	0.436	0.491	0.836	0.892
111	At5g05900	UDP-glucoronosyl/UDP-glucosyl transferase family pro	>0.05	0.025	0.560	0.326	0.572	0.868
112	At1g52050	jacalin lectin family protein	>0.05	0.009	0.323	0.118	0.320	0.776
113	At1g52060	similar to jacalin lectin family protein [Arabidopsis thal	>0.05	0.008	0.326	0.087	0.237	0.798
114	At3g26520	TIP2 (TONOPLAST INTRINSIC PROTEIN 2); water channe	0.045	0.002	0.662	0.432	0.632	0.846
115	At1g22530	PATL2; transporter	>0.05	0.001	0.603	0.392	0.554	0.800
116	At1g29490	auxin-responsive family protein	>0.05	0.043	0.617	0.443	0.675	0.872
117	At3g15450	similar to unknown protein [Arabidopsis thaliana] (TAII	>0.05	0.010	0.541	0.364	0.626	0.770
118	At4g12730	FLA2	0.024	0.001	0.611	0.428	0.657	0.817
119	At5g60660	PIP2;4/PIP2F (plasma membrane intrinsic protein 2;4);	>0.05	0.002	0.402	0.282	0.590	0.692
120	At5g28770	BZO2H3 (ARABIDOPSIS THALIANA BASIC LEUCINE ZIPPI	>0.05	0.047	0.628	0.445	0.732	1.002
121	At5g66815	unknown protein	>0.05	0.029	0.557	0.361	0.693	0.990
122	At4g11210	disease resistance-responsive family protein / dirigent	>0.05	0.011	0.562	0.364	0.680	0.907
123	At2g45040	matrix metalloproteinase	>0.05	0.009	0.680	0.472	0.799	0.949
124	At1g17190	ATGSTU26 (Arabidopsis thaliana Glutathione S-transfei	>0.05	0.009	0.620	0.394	0.777	0.958
125	At5g60520	late embryogenesis abundant protein-related / LEA prc	>0.05	0.007	0.393	0.262	0.629	1.050
126	At1g53680	ATGSTU28 (Arabidopsis thaliana Glutathione S-transfei	>0.05	0.040	0.660	0.447	0.869	0.938
127	At3g20940	CYP705A30 (cytochrome P450, family 705, subfamily A	>0.05	0.035	0.587	0.423	0.806	0.918
128	At5g33355	similar to unknown protein [Arabidopsis thaliana] (TAII	>0.05	0.007	0.500	0.202	0.530	0.808
129	At3g19850	phototropic-responsive NPH3 family protein	>0.05	0.010	0.656	0.398	0.698	0.853
130	At5g55480	glycerophosphoryl diester phosphodiesterase family pı	>0.05	0.042	0.697	0.441	0.750	0.875
131	At3g19430	late embryogenesis abundant protein-related / LEA prc	>0.05	0.008	0.613	0.330	0.690	0.851
132	At3g48940	remorin family protein	>0.05	0.029	0.634	0.350	0.780	0.817
133	At3g59930	similar to unknown protein [Arabidopsis thaliana] (TAII	>0.05	0.014	0.454	0.173	0.642	0.791
134	At1g52070	jacalin lectin family protein	0.048	0.006	0.357	0.126	0.397	1.155
135	At2g31085	CLE6 (CLAVATA3/ESR-RELATED 6); receptor binding	0.030	0.006	0.593	0.300	0.638	1.155
136	At2g31082	CLE7 (CLAVATA3/ESR-RELATED 7); receptor binding	>0.05	0.024	0.492	0.157	0.418	1.273
137	At3g21180	ACA9 (autoinhibited Ca2+ -ATPase 9); calcium-transpoı	>0.05	0.025	0.800	0.351	0.630	1.011
138	At5g22940	exostosin family protein	>0.05	0.043	0.793	0.457	0.849	0.959
139	At2g31081	CLE4 (CLAVATA3/ESR-RELATED 4); receptor binding	>0.05	0.009	0.798	0.268	0.652	0.792

140	At3g59020	protein transporter	>0.05	0.010	0.795	0.466	0.823	0.827
141	At5g19040	ATIPT5 (Arabidopsis thaliana isopentenyltransferase 5)	>0.05	0.012	0.542	0.416	0.531	1.067
142	At2g32300	UCC1 (UCLACYANIN 1); copper ion binding	>0.05	0.010	0.540	0.464	0.538	1.049
143	At5g60530	late embryogenesis abundant protein-related / LEA pro	>0.05	0.033	0.573	0.436	0.565	1.226
144	At4g14040	EDA38 (embryo sac development arrest 38); selenium	0.033	0.004	0.592	0.447	0.610	1.097
145	At2g19280	pentatricopeptide (PPR) repeat-containing protein	>0.05	0.039	0.558	0.310	0.445	1.123
146	At5g42180	peroxidase 64 (PER64) (P64) (PRXR4)	>0.05	0.013	0.469	0.566	0.589	0.997
147	At3g10710	pectinesterase family protein	>0.05	0.031	0.422	0.459	0.584	1.016
148	At3g50640	similar to unknown protein [Arabidopsis thaliana] (TAII	>0.05	0.019	0.445	0.457	0.561	1.060
149	At1g03870	FLA9	>0.05	0.003	0.469	0.358	0.478	0.832
150	At1g78000	SULTR1;2 (SULFATE TRANSPORTER 1;2); sulfate transm	>0.05	0.004	0.488	0.498	0.605	0.837
151	At2g28670	disease resistance-responsive family protein / fibroin-r	>0.05	0.006	0.457	0.431	0.514	0.883
152	At1g52240	ATROPGEF11/ROPGEF11 (KINASE PARTNER PROTEIN-L	>0.05	0.012	0.421	0.324	0.554	0.956
153	At5g62340	invertase/pectin methylesterase inhibitor family protei	>0.05	0.024	0.316	0.245	0.502	1.383
154	At4g30420	nodulin MtN21 family protein	0.049	0.012	0.525	0.486	0.617	1.236
155	At3g60550	CYCP3;2 (cyclin p3;2); cyclin-dependent protein kinase	0.033	0.013	0.517	0.474	0.727	1.292
156	At3g45070	sulfotransferase family protein	0.031	0.027	0.488	0.464	0.592	1.617
157	At2g17070	similar to unknown protein [Arabidopsis thaliana] (TAII	>0.05	0.039	0.471	0.579	0.493	1.410
158	At5g40730	AGP24 (ARABINOGALACTAN PROTEIN 24)	0.038	0.003	0.585	0.490	0.976	0.761
159	At5g56870	BGAL4 (beta-galactosidase 4); beta-galactosidase	0.018	0.001	0.285	0.208	0.883	0.452
160	At1g06120	fatty acid desaturase family protein	>0.05	0.003	0.409	0.347	0.809	0.587
161	At4g14980	DC1 domain-containing protein	0.026	0.008	0.532	0.355	1.241	0.696
162	At1g25230	purple acid phosphatase family protein	0.041	0.013	0.618	0.487	1.072	0.901
163	At1g31710	copper amine oxidase, putative	>0.05	0.038	0.781	0.498	0.942	0.656
164	At2g24710	ATGLR2.3 (Arabidopsis thaliana glutamate receptor 2.3	>0.05	0.038	0.599	0.235	0.755	0.536
165	At5g15725	unknown protein	>0.05	0.043	0.484	0.369	0.945	1.262
166	At1g54970	ATPRP1 (PROLINE-RICH PROTEIN 1); structural constitu	0.021	0.007	0.367	0.246	0.827	1.338
167	At1g16370	ATOCT6; carbohydrate transmembrane transporter/ su	0.027	0.008	0.488	0.471	0.915	1.106
168	At4g00680	actin-depolymerizing factor, putative	>0.05	0.017	0.461	0.396	0.971	0.927
169	At4g26010	peroxidase, putative	0.041	0.011	0.436	0.349	0.876	1.081
170	At5g04960	pectinesterase family protein	>0.05	0.021	0.437	0.303	0.908	0.998
171	At4g40090	AGP3 (ARABINOGALACTAN-PROTEIN 3)	0.039	0.008	0.548	0.421	0.851	1.035
172	At1g11655	similar to unknown protein [Arabidopsis thaliana] (TAII	>0.05	0.025	0.325	0.198	1.036	1.109
173	At4g34800	auxin-responsive family protein	0.028	0.026	0.581	0.371	1.073	1.146
174	At5g39320	UDP-glucose 6-dehydrogenase, putative	0.044	>0.05	0.520	0.500	1.045	1.362
175	At1g65310	ATXTH17 (XYLOGLUCAN ENDOTRANSGLUCOSYLASE/HY	0.019	0.023	0.364	0.375	1.107	1.491
176	At4g34580	transporter	0.039	0.044	0.333	0.386	0.941	1.522
177	At5g67400	peroxidase 73 (PER73) (P73) (PRXR11)	0.017	0.008	0.284	0.239	1.031	1.356
178	At2g41970	protein kinase, putative	0.031	0.026	0.421	0.445	1.074	1.220
179	At4g02270	pollen Ole e 1 allergen and extensin family protein	0.019	0.012	0.266	0.264	1.111	1.357
180	At3g09925	pollen Ole e 1 allergen and extensin family protein	0.022	0.017	0.466	0.474	1.031	1.242
181	At1g30870	cationic peroxidase, putative	0.034	>0.05	0.471	0.334	1.088	1.368
182	At1g75780	TUB1 (tubulin beta-1 chain); structural molecule	0.000	0.009	0.620	0.451	1.041	1.335
183	At4g25820	XTR9 (XYLOGLUCAN ENDOTRANSGLYCOSYLASE 9); hyd	0.025	>0.05	0.533	0.434	1.222	1.347
184	At5g47980	transferase family protein	>0.05	0.046	0.672	0.443	0.978	0.943
185	At4g31250	leucine-rich repeat transmembrane protein kinase, put	>0.05	0.017	0.661	0.388	0.935	0.926
186	At2g35990	similar to carboxy-lyase [Arabidopsis thaliana] (TAIR:AT	0.040	0.012	0.689	0.221	0.981	0.728
187	At3g09340	amino acid transporter family protein	0.016	0.007	0.654	0.414	0.878	1.217
188	At4g19030	NLM1 (NOD26-like intrinsic protein 1;1); water channe	>0.05	0.045	0.733	0.443	0.905	1.103

189	At1g24530	transducin family protein / WD-40 repeat family protei	0.008	0.004	0.827	0.457	0.976	1.118
190	At1g34510	peroxidase, putative	0.036	>0.05	0.613	0.301	1.286	1.223
191	At2g25240	serine-type endopeptidase inhibitor	0.041	>0.05	0.770	0.477	1.133	0.989
192	At5g22410	peroxidase, putative	0.009	>0.05	0.483	0.302	1.599	1.758
193	At1g13420	sulfotransferase family protein	0.049	>0.05	0.522	0.331	1.522	1.308
194	At4g25790	allergen V5/Tpx-1-related family protein	0.020	>0.05	0.439	0.362	1.580	1.276
195	At1g02810	pectinesterase family protein	0.031	>0.05	0.449	0.451	1.502	1.097
196	At4g21830	methionine sulfoxide reductase domain-containing prc	0.046	>0.05	0.476	0.487	1.490	1.034
197	At4g30320	allergen V5/Tpx-1-related family protein	0.030	0.028	0.352	0.335	1.314	1.070
198	At3g03190	ATGSTF11 (GLUTATHIONE S-TRANSFERASE F11); glutat	0.019	>0.05	0.466	0.649	1.133	1.522
199	At3g18000	NMT1 (N-METHYLTRANSFERASE 1); phosphoethanolan	0.015	>0.05	0.416	0.469	1.516	1.472
200	At4g29690	type I phosphodiesterase/nucleotide pyrophosphatase	0.044	>0.05	0.351	0.446	1.354	1.691
201	At5g20860	pectinesterase family protein	>0.05	0.021	0.386	0.575	1.046	0.919
202	At3g19390	cysteine proteinase, putative / thiol protease, putative	>0.05	0.022	0.466	0.640	0.940	0.949
203	At1g26250	proline-rich extensin, putative	>0.05	0.048	0.493	0.703	0.900	0.854
204	At5g57625	allergen V5/Tpx-1-related family protein	>0.05	0.027	0.421	0.478	0.984	0.988
205	At3g22710	F-box family protein	>0.05	0.037	0.383	0.460	1.005	0.881
206	At2g30210	LAC3 (laccase 3); copper ion binding / oxidoreductase	0.031	0.010	0.351	0.380	1.115	0.940
207	At1g62980	ATEXPA18 (ARABIDOPSIS THALIANA EXPANSIN A18)	>0.05	0.023	0.397	0.436	1.040	0.750
208	At2g30395	ATOFP17/OFP17 (Arabidopsis thaliana ovate family prc	>0.05	0.047	0.394	0.454	1.153	0.760
209	At5g38930	germin-like protein, putative	0.021	0.017	0.456	0.605	1.090	1.115
210	At5g38940	manganese ion binding / metal ion binding / nutrient r	0.019	0.021	0.389	0.585	1.165	1.149
211	At4g30170	peroxidase, putative	0.046	0.034	0.361	0.529	1.172	0.982
212	At2g37130	peroxidase 21 (PER21) (P21) (PRXR5)	0.023	0.014	0.332	0.530	0.911	1.262
213	At1g47840	hexokinase, putative	>0.05	0.027	0.468	0.835	0.888	0.875
214	At2g21880	AtRABG2/AtRab7A (Arabidopsis Rab GTPase homolog (	0.049	0.018	0.446	0.801	0.951	0.893
215	At5g39240	similar to unknown protein [Arabidopsis thaliana] (TAII	0.044	>0.05	1.004	0.496	0.825	1.177
216	At2g43880	polygalacturonase, putative / pectinase, putative	0.046	>0.05	0.906	0.401	0.807	1.458
217	At1g51470	glycosyl hydrolase family 1 protein	0.033	>0.05	0.706	0.315	0.809	2.280
218	At2g25160	CYP82F1 (cytochrome P450, family 82, subfamily F, pol	0.015	0.020	0.761	0.430	0.676	1.554
219	At3g62760	ATGSTF13 (Arabidopsis thaliana Glutathione S-transfer	0.050	>0.05	0.535	0.306	0.652	1.731
220	At1g16410	CYP79F1 (SUPERSHOOT 1)	0.022	>0.05	0.349	0.324	0.667	2.359
221	At1g59940	ARR3 (RESPONSE REGULATOR 3); transcription regulat	0.042	>0.05	0.803	0.603	1.040	2.138
222	At1g14960	major latex protein-related / MLP-related	0.018	>0.05	0.512	0.302	1.003	2.197
223	At3g49960	peroxidase, putative	0.026	>0.05	0.570	0.402	1.156	2.335
224	At4g38860	auxin-responsive protein, putative	0.031	>0.05	0.853	0.490	1.240	1.526
225	At5g06200	integral membrane family protein	>0.05	0.011	0.544	0.571	0.491	0.662
226	At5g59090	ATSBT4.12; subtilase	>0.05	0.002	0.506	0.484	0.438	0.605
227	At2g36830	GAMMA-TIP (Tonoplast intrinsic protein (TIP) gamma);	>0.05	0.001	0.408	0.404	0.334	0.530
228	At4g15660	glutaredoxin family protein	>0.05	0.001	0.179	0.178	0.141	0.308
229	At4g15690	glutaredoxin family protein	>0.05	0.001	0.236	0.189	0.174	0.360
230	At4g15700	glutaredoxin family protein	>0.05	0.001	0.272	0.214	0.208	0.375
231	At1g73280	SCPL3 (serine carboxypeptidase-like 3); serine carboxy	>0.05	0.004	0.499	0.585	0.499	0.717
232	At2g34000	zinc finger protein-related	>0.05	0.009	0.354	0.401	0.288	0.583
233	At3g23730	xyloglucan:xyloglucosyl transferase, putative / xylogluc	>0.05	0.004	0.587	0.522	0.468	0.721
234	At3g26610	polygalacturonase, putative / pectinase, putative	>0.05	0.004	0.327	0.218	0.196	0.542
235	At5g22920	zinc finger (C3HC4-type RING finger) family protein	>0.05	0.004	0.587	0.527	0.432	0.498
236	At1g72200	zinc finger (C3HC4-type RING finger) family protein	>0.05	0.011	0.629	0.570	0.498	0.561
237	At3g24300	AMT1;3/ATAMT1;3 (AMMONIUM TRANSPORTER 1;3);	>0.05	0.005	0.532	0.459	0.375	0.489

238	At4g15400	transferase family protein	>0.05	0.003	0.563	0.500	0.427	0.544
239	At3g01190	peroxidase 27 (PER27) (P27) (PRXR7)	>0.05	0.001	0.234	0.246	0.188	0.288
240	At3g48100	ARR5 (ARABIDOPSIS RESPONSE REGULATOR 5); transcr	>0.05	0.001	0.550	0.569	0.494	0.581
241	At3g61430	PIP1A (PLASMA MEMBRANE INTRINSIC PROTEIN 1A); w	>0.05	0.002	0.501	0.544	0.420	0.536
242	At3g45700	proton-dependent oligopeptide transport (POT) family	>0.05	0.000	0.529	0.464	0.433	0.539
243	At4g15680	glutaredoxin family protein	>0.05	0.001	0.366	0.332	0.297	0.393
244	At5g23360	GRAM domain-containing protein / ABA-responsive pro	>0.05	0.003	0.520	0.537	0.370	0.531
245	At3g06070	similar to unknown protein [Arabidopsis thaliana] (TAII	>0.05	0.008	0.623	0.661	0.480	0.648
246	At5g48430	aspartic-type endopeptidase/ pepsin A	>0.05	0.004	0.613	0.612	0.442	0.548
247	At4g17340	DELTA-TIP2/TIP2;2 (tonoplast intrinsic protein 2;2); wa	>0.05	0.002	0.455	0.454	0.248	0.391
248	At4g29905	similar to unknown protein [Arabidopsis thaliana] (TAII	>0.05	0.002	0.374	0.434	0.223	0.346
249	At5g65730	xyloglucan:xyloglucosyl transferase, putative / xylogluc	>0.05	0.004	0.412	0.515	0.352	0.571
250	At3g55150	ATEXO70H1 (exocyst subunit EXO70 family protein H1)	>0.05	0.004	0.453	0.509	0.353	0.546
251	At2g19800	MIOX2 (MYO-INOSITOL OXYGENASE 2)	>0.05	0.004	0.401	0.548	0.322	0.494
252	At4g26260	MIOX4 (MYO-INOSITOL OXYGENASE 4)	>0.05	0.007	0.276	0.464	0.295	0.430
253	At5g15180	peroxidase, putative	>0.05	0.006	0.579	0.421	0.370	0.608
254	At4g37540	LBD39 (LOB DOMAIN-CONTAINING PROTEIN 39)	>0.05	0.003	0.692	0.532	0.482	0.697
255	At5g15290	integral membrane family protein	>0.05	0.012	0.522	0.359	0.322	0.524
256	At2g33790	pollen Ole e 1 allergen and extensin family protein	>0.05	0.004	0.365	0.204	0.166	0.300
257	At3g62950	glutaredoxin family protein	>0.05	0.003	0.386	0.236	0.163	0.355
258	At1g80380	phosphoribulokinase/uridine kinase-related	>0.05	0.000	0.696	0.482	0.453	0.615
259	At3g18780	ACT2 (ACTIN 2); structural constituent of cytoskeleton	>0.05	0.000	0.750	0.549	0.493	0.682
260	At2g02610	DC1 domain-containing protein	>0.05	0.003	0.621	0.476	0.353	0.548
261	At4g22460	protease inhibitor/seed storage/lipid transfer protein (	>0.05	0.005	0.596	0.454	0.352	0.474
262	At5g20250	DIN10 (DARK INDUCIBLE 10); hydrolase, hydrolyzing O-	>0.05	0.002	0.530	0.339	0.368	0.508
263	At1g13300	myb family transcription factor	>0.05	0.013	0.623	0.473	0.501	0.562
264	At1g55380	DC1 domain-containing protein	>0.05	0.049	0.709	0.493	0.494	0.563
265	At2g38080	IRX12/LAC4 (laccase 4); copper ion binding / oxidoredu	>0.05	0.008	0.602	0.347	0.388	0.454
266	At3g30775	ERD5 (EARLY RESPONSIVE TO DEHYDRATION 5); proline	>0.05	0.002	0.602	0.371	0.424	0.500
267	At5g19190	similar to unknown protein [Arabidopsis thaliana] (TAII	>0.05	0.002	0.672	0.449	0.385	0.468
268	At3g48360	BT2 (BTB AND TAZ DOMAIN PROTEIN 2); protein bindir	>0.05	0.002	0.678	0.285	0.260	0.338
269	At4g19690	IRT1 (IRON-REGULATED TRANSPORTER 1); cadmium ioi	>0.05	0.001	0.601	0.275	0.270	0.301
270	At3g13750	BGAL1 (BETA GALACTOSIDASE 1); beta-galactosidase	>0.05	0.002	0.807	0.482	0.562	0.560
271	At5g21940	similar to unknown protein [Arabidopsis thaliana] (TAII	>0.05	0.004	0.691	0.581	0.390	0.656
272	At1g49500	similar to unknown protein [Arabidopsis thaliana] (TAII	>0.05	0.008	0.690	0.580	0.390	0.648
273	At3g02850	SKOR (stelar K+ outward rectifier); cyclic nucleotide bir	>0.05	0.014	0.557	0.416	0.204	0.443
274	At4g17460	HAT1 (homeobox-leucine zipper protein 1); DNA bindir	>0.05	0.006	0.724	0.676	0.459	0.682
275	At3g06080	similar to unknown protein [Arabidopsis thaliana] (TAII	>0.05	0.007	0.745	0.624	0.421	0.726
276	At1g22230	similar to unknown protein [Arabidopsis thaliana] (TAII	>0.05	0.028	0.782	0.612	0.479	0.693
277	At5g25810	TNY (TINY); DNA binding / transcription factor	>0.05	0.002	0.538	0.298	0.176	0.340
278	At3g05950	germin-like protein, putative	>0.05	0.004	0.669	0.430	0.260	0.470
279	At1g63180	UGE3 (UDP-D-GLUCOSE/UDP-D-GALACTOSE 4-EPIMERA	>0.05	0.022	0.779	0.635	0.482	0.621
280	At3g16690	nodulin MtN3 family protein	>0.05	0.016	0.782	0.546	0.433	0.550
281	At3g57520	ATSIP2 (ARABIDOPSIS THALIANA SEED IMBIBITION 2); I	>0.05	0.000	0.739	0.529	0.414	0.542
282	At1g72610	GLP1 (GERMIN-LIKE PROTEIN 1); manganese ion bindir	>0.05	0.006	0.831	0.579	0.427	0.582
283	At5g06570	hydrolase	>0.05	0.017	0.494	0.750	0.328	0.435
284	At3g25190	nodulin, putative	>0.05	0.005	0.588	0.829	0.470	0.516
285	At5g17300	myb family transcription factor	>0.05	0.011	0.653	0.765	0.431	0.517
286	At4g04840	methionine sulfoxide reductase domain-containing prc	0.028	0.001	0.407	0.660	0.196	0.261

287	At4g08360	KOW domain-containing protein	>0.05	0.008	0.462	0.672	0.244	0.301
288	At2g22630	AGL17 (AGAMOUS-LIKE 17); transcription factor	>0.05	0.002	0.495	0.805	0.274	0.340
289	At1g70970	F-box family protein	>0.05	0.002	0.454	0.698	0.372	0.434
290	At3g45690	proton-dependent oligopeptide transport (POT) family	>0.05	0.005	0.583	0.766	0.500	0.586
291	At4g18940	cyclic phosphodiesterase, putative	>0.05	0.003	0.415	0.786	0.404	0.473
292	At5g14070	ROXY2; thiol-disulfide exchange intermediate	>0.05	0.019	0.526	0.573	0.353	0.441
293	At4g30450	glycine-rich protein	>0.05	0.002	0.515	0.604	0.357	0.440
294	At5g41080	glycerophosphoryl diester phosphodiesterase family pı	>0.05	0.001	0.575	0.551	0.348	0.454
295	At1g22740	RAB7 (Ras-related protein 7); GTP binding	>0.05	0.005	0.676	0.668	0.484	0.540
296	At1g30840	ATPUP4 (Arabidopsis thaliana purine permease 4); pur	>0.05	0.002	0.589	0.691	0.461	0.477
297	At5g43350	ATPT1 (PHOSPHATE TRANSPORTER 1); carbohydrate tr	>0.05	0.002	0.552	0.597	0.398	0.411
298	At1g70850	MLP34 (MLP-LIKE PROTEIN 34)	>0.05	0.031	0.619	0.688	0.495	0.497
299	At5g43370	APT1/PHT1;2/PHT2 (PHOSPHATE TRANSPORTER 2); caı	>0.05	0.002	0.565	0.653	0.450	0.428
300	At2g01880	ATPAP7/PAP7 (purple acid phosphatase 7); acid phosp	>0.05	0.003	0.606	0.712	0.446	0.503
301	At1g30360	ERD4 (EARLY-RESPONSIVE TO DEHYDRATION 4)	>0.05	0.001	0.702	0.624	0.436	0.530
302	At2g18700	ATTPS11 (Arabidopsis thaliana trehalose phosphatase/	>0.05	0.008	0.724	0.646	0.456	0.595
303	At2g01530	MLP329 (MLP-LIKE PROTEIN 329)	>0.05	0.003	0.578	0.540	0.280	0.411
304	At1g11380	similar to unknown protein [Arabidopsis thaliana] (TAII	>0.05	0.014	0.702	0.704	0.464	0.586
305	At1g66230	MYB20 (myb domain protein 20); DNA binding / transc	>0.05	0.011	0.670	0.655	0.391	0.463
306	At1g73165	CLE1 (CLAVATA3/ESR-RELATED 1); receptor binding	>0.05	0.034	0.520	0.441	0.143	0.273
307	At5g48010	pentacyclic triterpene synthase, putative	>0.05	0.016	0.666	0.768	0.493	0.701
308	At4g39070	zinc finger (B-box type) family protein	>0.05	0.007	0.500	0.574	0.249	0.521
309	At1g67710	ARR11 (RESPONSE REGULATOR 11); transcription facto	>0.05	0.008	0.686	0.735	0.499	0.656
310	At1g74760	zinc finger (C3HC4-type RING finger) family protein cor	>0.05	0.002	0.564	0.524	0.246	0.445
311	At4g37410	CYP81F4 (cytochrome P450, family 81, subfamily F, pol	>0.05	0.004	0.641	0.685	0.383	0.588
312	At3g61060	ATPP2-A13	>0.05	0.004	0.565	0.696	0.309	0.492
313	At5g24410	glucosamine/galactosamine-6-phosphate isomerase-re	>0.05	0.001	0.379	0.414	0.312	0.320
314	At5g47450	AtTIP2;3 (Arabidopsis thaliana tonoplast intrinsic prote	>0.05	0.002	0.261	0.288	0.164	0.152
315	At3g45710	proton-dependent oligopeptide transport (POT) family	>0.05	0.001	0.532	0.526	0.393	0.412
316	At5g63160	BT1 (BTB and TAZ domain protein 1); protein binding /	>0.05	0.003	0.590	0.556	0.513	0.418
317	At3g46880	similar to unknown protein [Arabidopsis thaliana] (TAII	>0.05	0.004	0.663	0.580	0.554	0.494
318	At4g12545	protease inhibitor/seed storage/lipid transfer protein (	>0.05	0.001	0.291	0.247	0.175	0.149
319	At2g42170	actin, putative	>0.05	0.012	0.680	0.577	0.465	0.507
320	At3g01760	lysine and histidine specific transporter, putative	>0.05	0.043	0.585	0.469	0.461	0.455
321	At1g02640	BXL2 (BETA-XYLOSIDASE 2); hydrolase, hydrolyzing O-g	>0.05	0.030	0.601	0.433	0.482	0.429
322	At1g76410	ATL8; protein binding / zinc ion binding	>0.05	0.005	0.609	0.523	0.416	0.349
323	At4g12550	AIR1 (Auxin-Induced in Root cultures 1); lipid binding	>0.05	0.004	0.513	0.449	0.340	0.251
324	At4g37700	similar to unknown protein [Arabidopsis thaliana] (TAII	>0.05	0.001	0.453	0.468	0.254	0.201
325	At2g22122	unknown protein	>0.05	0.001	0.577	0.354	0.319	0.221
326	At1g14185	glucose-methanol-choline (GMC) oxidoreductase famil	>0.05	0.018	0.719	0.585	0.579	0.444
327	At1g33055	unknown protein	>0.05	0.004	0.401	0.475	0.453	0.319
328	At1g05650	polygalacturonase, putative / pectinase, putative	>0.05	0.034	0.544	0.591	0.572	0.429
329	At2g04800	unknown protein	>0.05	0.008	0.604	0.705	0.666	0.491
330	At4g39675	unknown protein	>0.05	0.001	0.252	0.394	0.319	0.134
331	At1g78120	tetratricopeptide repeat (TPR)-containing protein	>0.05	0.003	0.452	0.627	0.409	0.413
332	At2g37750	unknown protein	>0.05	0.002	0.393	0.530	0.396	0.329
333	At1g01580	FRO2 (FERRIC REDUCTION OXIDASE 2); ferric-chelate re	>0.05	0.013	0.591	0.682	0.537	0.334
334	At1g07610	MT1C (metallothionein 1C)	>0.05	0.005	0.562	0.686	0.524	0.389
335	At4g24015	zinc finger (RING-H2 type) protein-related	>0.05	0.002	0.583	0.703	0.510	0.420

336	At1g31050	transcription factor	>0.05	0.023	0.552	0.691	0.447	0.754
337	At1g07560	leucine-rich repeat protein kinase, putative	>0.05	0.022	0.487	0.681	0.445	0.683
338	At2g14160	nucleic acid binding	>0.05	0.010	0.390	0.654	0.395	0.765
339	At4g15670	glutaredoxin family protein	>0.05	0.006	0.292	0.528	0.267	0.660
340	At3g05890	RCI2B (RARE-COLD-INDUCIBLE 2B)	>0.05	0.006	0.539	0.756	0.498	0.792
341	At3g51910	AT-HSFA7A (Arabidopsis thaliana heat shock transcript	>0.05	0.016	0.555	0.762	0.391	0.658
342	At5g03310	auxin-responsive family protein	>0.05	0.007	0.478	0.782	0.446	0.670
343	At5g03570	iron-responsive transporter-related	>0.05	0.008	0.350	0.764	0.264	0.461
344	At5g42580	CYP705A12 (cytochrome P450, family 705, subfamily A	>0.05	0.017	0.410	0.807	0.619	0.701
345	At1g49030	similar to unknown protein [Arabidopsis thaliana] (TAII	>0.05	0.002	0.286	0.592	0.405	0.396
346	At2g01900	endonuclease/exonuclease/phosphatase family protei	>0.05	0.004	0.319	0.633	0.376	0.435
347	At4g15340	ATPEN1 (Arabidopsis thaliana pentacyclic triterpene sy	>0.05	0.002	0.224	0.473	0.374	0.495
348	At4g38390	similar to unknown protein [Arabidopsis thaliana] (TAII	>0.05	0.008	0.447	0.654	0.649	0.613
349	At5g09530	hydroxyproline-rich glycoprotein family protein	>0.05	0.006	0.686	0.411	0.494	0.627
350	At1g50560	CYP705A25 (cytochrome P450, family 705, subfamily A	>0.05	0.011	0.776	0.498	0.610	0.739
351	At2g37440	endonuclease/exonuclease/phosphatase family protei	>0.05	0.026	0.745	0.399	0.582	0.625
352	At1g45015	MD-2-related lipid recognition domain-containing prot	>0.05	0.013	0.511	0.253	0.407	0.626
353	At3g22970	similar to unknown protein [Arabidopsis thaliana] (TAII	>0.05	0.039	0.723	0.454	0.594	0.810
354	At2g42870	HLH1/PAR1 (PHY RAPIDLY REGULATED 1); transcriptior	>0.05	0.030	0.695	0.408	0.498	0.743
355	At5g18600	glutaredoxin family protein	>0.05	0.003	0.430	0.319	0.467	0.593
356	At5g53250	AGP22/ATAGP22 (ARABINOGALACTAN PROTEINS 22)	>0.05	0.001	0.273	0.158	0.274	0.420
357	At4g12510	protease inhibitor/seed storage/lipid transfer protein (	>0.05	0.004	0.318	0.189	0.310	0.470
358	At4g12520	protease inhibitor/seed storage/lipid transfer protein (	>0.05	0.004	0.330	0.220	0.317	0.465
359	At1g19900	glyoxal oxidase-related	>0.05	0.012	0.351	0.262	0.364	0.474
360	At4g15290	ATCSLB05 (Cellulose synthase-like B5); transferase/ tra	>0.05	0.005	0.395	0.310	0.402	0.517
361	At5g19970	similar to unnamed protein product [Vitis vinifera] (GB	>0.05	0.012	0.561	0.360	0.507	0.585
362	At1g52750	hydrolase, alpha/beta fold family protein	>0.05	0.010	0.592	0.454	0.537	0.655
363	At4g26320	AGP13 (ARABINOGALACTAN PROTEIN 13)	>0.05	0.001	0.208	0.097	0.188	0.284
364	At1g12080	contains domain PTHR22683 (PTHR22683)	>0.05	0.003	0.557	0.318	0.482	0.608
365	At4g34790	auxin-responsive family protein	>0.05	0.006	0.569	0.372	0.532	0.645
366	At3g59370	contains domain PTHR22683 (PTHR22683)	>0.05	0.004	0.403	0.221	0.363	0.544
367	At3g25790	myb family transcription factor	>0.05	0.004	0.624	0.434	0.619	0.675
368	At5g24100	leucine-rich repeat transmembrane protein kinase, put	>0.05	0.012	0.529	0.393	0.497	0.760
369	At5g56540	AGP14 (ARABINOGALACTAN PROTEIN 14)	>0.05	0.004	0.407	0.248	0.405	0.626
370	At3g56000	ATCSLA14 (Cellulose synthase-like A14); transferase, tr	>0.05	0.002	0.511	0.357	0.442	0.672
371	At5g44020	acid phosphatase class B family protein	>0.05	0.001	0.585	0.333	0.612	0.537
372	At4g25250	invertase/pectin methylesterase inhibitor family protei	>0.05	0.018	0.676	0.473	0.736	0.624
373	At4g18510	CLE2 (CLAVATA3/ESR-RELATED); receptor binding	>0.05	0.001	0.284	0.151	0.399	0.397
374	ath-MIR169a	miRNA gene Arabidopsis thaliana miR169a stem-loop	>0.05	0.010	0.619	0.423	0.583	0.542
375	At2g28270	DC1 domain-containing protein	>0.05	0.046	0.617	0.425	0.586	0.549
376	At1g16390	ATOCT3 (ARABIDOPSIS THALIANA ORGANIC CATION/C/	>0.05	0.001	0.576	0.441	0.519	0.506
377	At5g14750	ATMYB66/WER/WER1 (WEREWOLF 1); DNA binding / p	>0.05	0.006	0.520	0.479	0.666	0.737
378	At1g03700	integral membrane family protein	>0.05	0.009	0.547	0.495	0.711	0.751
379	At2g19660	DC1 domain-containing protein	>0.05	0.022	0.585	0.495	0.695	0.752
380	At5g42785	unknown protein	>0.05	0.004	0.350	0.348	0.543	0.662
381	At3g18450	similar to unknown protein [Arabidopsis thaliana] (TAII	>0.05	0.007	0.170	0.176	0.380	0.496
382	At5g49360	BXL1 (BETA-XYLOSIDASE 1); hydrolase, hydrolyzing O-g	>0.05	0.009	0.358	0.391	0.561	0.657
383	At5g64100	peroxidase, putative	>0.05	0.002	0.367	0.389	0.530	0.546
384	At4g35100	PIP3 (PLASMA MEMBRANE INTRINSIC PROTEIN 3); wat	>0.05	0.001	0.499	0.527	0.611	0.675

385	At2g30930	similar to unknown protein [Arabidopsis thaliana] (TAII	>0.05	0.004	0.414	0.473	0.597	0.683
386	At4g22210	LCR85 (Low-molecular-weight cysteine-rich 85)	>0.05	0.006	0.362	0.400	0.537	0.599
387	At5g17820	peroxidase 57 (PER57) (P57) (PRXR10)	>0.05	0.003	0.487	0.391	0.759	0.670
388	At4g08620	SULTR1;1 (sulfate transporter 1;1); sulfate transmembr	>0.05	0.004	0.494	0.440	0.713	0.584
389	At5g36180	SCPL1 (serine carboxypeptidase-like 1); serine carboxy|	>0.05	0.002	0.418	0.461	0.448	0.771
390	At1g62320	early-responsive to dehydration protein-related / ERD	>0.05	0.008	0.398	0.466	0.464	0.682
391	At4g33610	glycine-rich protein	>0.05	0.041	0.446	0.604	0.496	0.735
392	At5g38450	CYP735A1 (cytochrome P450, family 735, subfamily A,	>0.05	0.001	0.315	0.423	0.482	0.349
393	At2g18800	xyloglucan:xyloglucosyl transferase, putative / xygluc	>0.05	0.008	0.453	0.500	0.598	0.432
394	At4g22212	similar to LCR85 (Low-molecular-weight cysteine-rich 8	>0.05	0.002	0.309	0.388	0.496	0.303
395	At2g37180	RD28 (plasma membrane intrinsic protein 2;3); water c	>0.05	0.001	0.388	0.464	0.479	0.401
396	At3g13760	DC1 domain-containing protein	>0.05	0.004	0.468	0.532	0.532	0.464
397	At2g44380	DC1 domain-containing protein	>0.05	0.002	0.398	0.475	0.481	0.485
398	At1g23870	ATTPS9 (Arabidopsis thaliana trehalose-phosphatase/s	>0.05	0.002	0.467	0.531	0.516	0.510
399	At4g13620	AP2 domain-containing transcription factor, putative	>0.05	0.021	0.465	0.586	0.574	0.568
400	At5g46890	protease inhibitor/seed storage/lipid transfer protein (	>0.05	0.003	0.342	0.262	0.394	0.360
401	At5g47950	transferase family protein	>0.05	0.004	0.360	0.290	0.397	0.365
402	At4g37220	stress-responsive protein, putative	>0.05	0.011	0.400	0.409	0.531	0.464
403	At1g01620	PIP1C (PLASMA MEMBRANE INTRINSIC PROTEIN 1;3); \	>0.05	0.001	0.451	0.491	0.544	0.615
404	At2g37170	PIP2B (plasma membrane intrinsic protein 2;2); water (	>0.05	0.000	0.352	0.391	0.433	0.500
405	At3g49330	invertase/pectin methylesterase inhibitor family protei	>0.05	0.002	0.442	0.463	0.502	0.545
406	At1g15040	glutamine amidotransferase-related	>0.05	0.002	0.424	0.501	0.580	0.543
407	At3g11385	DC1 domain-containing protein	>0.05	0.007	0.300	0.430	0.457	0.487
408	At1g33700	catalytic	>0.05	0.008	0.395	0.407	0.597	0.518
409	At4g22230	similar to unknown protein [Arabidopsis thaliana] (TAII	>0.05	0.000	0.219	0.286	0.460	0.344
410	At3g22570	protease inhibitor/seed storage/lipid transfer protein (	>0.05	0.004	0.308	0.445	0.582	0.449
411	At2g22330	CYP79B3 (cytochrome P450, family 79, subfamily B, po	>0.05	0.002	0.429	0.493	0.757	0.563
412	At5g07190	ATS3 (ARABIDOPSIS THALIANA SEED GENE 3)	>0.05	0.031	0.724	0.490	0.366	1.272
413	At5g08250	cytochrome P450 family protein	>0.05	0.038	0.655	0.567	0.463	0.856
414	At3g45720	proton-dependent oligopeptide transport (POT) family	>0.05	0.007	0.642	0.483	0.423	0.860
415	At3g50120	similar to unknown protein [Arabidopsis thaliana] (TAII	>0.05	0.006	0.649	0.526	0.442	0.895
416	At5g61440	thioredoxin family protein	>0.05	0.002	0.662	0.446	0.424	0.783
417	At5g35940	jacalin lectin family protein	>0.05	0.008	0.471	0.305	0.365	0.767
418	At1g75750	GASA1 (GAST1 PROTEIN HOMOLOG 1)	>0.05	0.014	0.526	0.352	0.400	0.840
419	At2g39040	peroxidase, putative	>0.05	0.008	0.461	0.326	0.358	0.834
420	At2g23130	AGP17 (ARABINOGALACTAN PROTEIN 17)	>0.05	0.006	0.491	0.429	0.376	1.090
421	At3g62570	DNAJ heat shock N-terminal domain-containing protei	0.029	0.003	0.562	0.522	0.375	1.102
422	At5g24030	SLAH3 (SLAC1 HOMOLOGUE 3); transporter	>0.05	0.017	0.747	0.671	0.350	0.949
423	At2g05540	glycine-rich protein	>0.05	0.019	0.761	0.698	0.397	0.956
424	At1g64780	ATAMT1;2 (AMMONIUM TRANSPORTER 1;2); ammoniu	>0.05	0.013	0.749	0.682	0.308	0.865
425	At2g07678	Identical to Uncharacterized mitochondrial protein AtM	>0.05	0.041	0.805	0.708	0.487	1.027
426	At5g02350	DC1 domain-containing protein	>0.05	0.019	0.608	0.506	0.282	1.086
427	At2g30520	RPT2 (ROOT PHOTOTROPISM 2); protein binding	>0.05	0.011	0.666	0.678	0.426	1.013
428	At1g22460	similar to unknown protein [Arabidopsis thaliana] (TAII	>0.05	0.044	0.648	0.774	0.411	1.022
429	At5g44110	POP1	>0.05	0.007	0.651	0.738	0.457	0.912
430	At1g68440	similar to unknown protein [Arabidopsis thaliana] (TAII	>0.05	0.007	0.682	0.772	0.452	0.894
431	At1g21910	AP2 domain-containing transcription factor family prot	>0.05	0.026	0.612	0.714	0.424	0.788
432	At2g20670	similar to unknown protein [Arabidopsis thaliana] (TAII	>0.05	0.006	0.564	0.547	0.259	0.677
433	At1g02660	lipase class 3 family protein	>0.05	0.010	0.706	0.636	0.446	0.831

434	At5g44130	FLA13 (FASCICLIN-LIKE ARABINOGALACTAN PROTEIN 1	>0.05	0.007	0.812	0.457	0.315	0.781
435	At4g04850	KEA3 (K+ efflux antiporter 3); potassium:hydrogen anti	>0.05	0.012	0.840	0.557	0.435	0.872
436	At1g12740	CYP87A2 (cytochrome P450, family 87, subfamily A, po	>0.05	0.007	0.728	0.383	0.284	0.764
437	At2g28190	CSD2 (COPPER/ZINC SUPEROXIDE DISMUTASE 2); copp	>0.05	0.004	0.833	0.569	0.497	0.865
438	At5g53500	WD-40 repeat family protein	0.017	0.001	0.918	0.549	0.424	0.813
439	At2g32740	exostosin family protein	>0.05	0.019	0.933	0.473	0.395	0.829
440	At1g33340	epsin N-terminal homology (ENTH) domain-containing	>0.05	0.019	0.849	0.560	0.408	0.746
441	At2g40230	transferase family protein	>0.05	0.003	0.723	0.461	0.279	0.681
442	At1g14430	glyoxal oxidase-related	>0.05	0.027	0.838	0.625	0.485	0.771
443	At1g72430	auxin-responsive protein-related	>0.05	0.008	0.861	0.625	0.463	0.714
444	At1g19510	myb family transcription factor	>0.05	0.010	0.818	0.592	0.372	0.837
445	At2g25430	epsin N-terminal homology (ENTH) domain-containing	>0.05	0.044	1.105	0.447	0.386	0.826
446	At4g34620	SSR16 (ribosomal protein S16); structural constituent c	0.035	0.004	1.028	0.569	0.484	0.766
447	At3g16880	F-box protein-related	0.031	0.007	0.991	0.309	0.304	0.869
448	At1g06830	glutaredoxin family protein	>0.05	0.014	0.975	0.477	0.657	0.705
449	At2g40670	ARR16 (response regulator 16); transcription regulator	>0.05	0.050	0.999	0.402	0.553	0.880
450	At3g09580	amine oxidase family protein	>0.05	0.020	0.980	0.493	0.683	0.899
451	At3g50780	protein binding	>0.05	0.040	0.841	0.468	0.699	0.862
452	At4g29800	PLA IVD/PLP8 (Patatin-like protein 8)	>0.05	0.015	0.721	0.322	0.498	0.755
453	At3g24260	similar to unknown protein [Arabidopsis thaliana] (TAII	>0.05	0.038	1.545	0.328	0.348	0.605
454	At5g08150	unknown protein	>0.05	0.019	0.802	0.836	0.460	0.632
455	At3g23530	cyclopropane fatty acid synthase, putative / CPA-FA sy	>0.05	0.007	0.863	0.799	0.456	0.617
456	At3g56980	BHLH039/ORG3 (OBP3-RESPONSIVE GENE 3); DNA binc	>0.05	0.012	0.770	0.721	0.241	0.406
457	At5g25240	similar to NHL repeat-containing protein [Arabidopsis t	>0.05	0.042	0.841	0.756	0.473	0.568
458	At3g59340	similar to unknown protein [Arabidopsis thaliana] (TAII	>0.05	0.002	0.764	0.725	0.351	0.478
459	At1g04240	SHY2 (SHORT HYPOCOTYL 2); transcription factor	0.041	0.001	0.730	0.574	0.270	0.448
460	At3g19680	similar to unknown protein [Arabidopsis thaliana] (TAII	>0.05	0.017	0.854	0.705	0.448	0.612
461	At1g54740	similar to structural constituent of ribosome [Arabidop	>0.05	0.005	0.854	0.737	0.431	0.612
462	At2g39570	ACT domain-containing protein	>0.05	0.003	0.791	0.671	0.405	0.625
463	At5g01015	similar to unknown protein [Arabidopsis thaliana] (TAII	>0.05	0.014	0.793	0.632	0.410	0.633
464	At1g03440	leucine-rich repeat family protein	0.030	0.003	0.847	0.754	0.393	0.717
465	At2g28305	similar to unknown protein [Arabidopsis thaliana] (TAII	0.032	0.002	0.803	0.721	0.316	0.566
466	At1g17345	auxin-responsive protein-related	>0.05	0.047	0.786	0.770	0.403	0.640
467	At5g56550	similar to unknown protein [Arabidopsis thaliana] (TAII	>0.05	0.015	0.678	0.778	0.290	0.612
468	At1g35140	PHI-1 (PHOSPHATE-INDUCED 1)	>0.05	0.027	0.689	0.746	0.281	0.639
469	At5g59520	ZIP2 (ZINC TRANSPORTER 2 PRECURSOR); transferase, '	>0.05	0.017	0.802	0.916	0.455	0.700
470	At3g57020	strictosidine synthase family protein	0.042	0.003	0.688	0.785	0.253	0.498
471	At3g25820	ATTPS-CIN (TERPENE SYNTHASE-LIKE SEQUENCE-1,8-CI	0.042	0.007	0.592	1.034	0.217	0.511
472	At3g25830	ATTPS-CIN (TERPENE SYNTHASE-LIKE SEQUENCE-1,8-CI	0.042	0.005	0.604	0.936	0.231	0.509
473	ath-MIR163	miRNA gene Arabidopsis thaliana miR163 stem-loop	0.036	0.013	0.652	1.118	0.407	0.763
474	At3g12955	auxin-responsive protein-related	0.034	0.009	0.496	1.314	0.233	0.471
475	At1g64590	short-chain dehydrogenase/reductase (SDR) family prc	>0.05	0.028	0.657	0.964	0.388	0.829
476	At5g02270	ATNAP9 (Non-intrinsic ABC protein 9)	0.023	0.003	0.753	0.920	0.491	0.856
477	At5g14470	GHMP kinase-related	>0.05	0.038	1.112	0.829	0.395	0.570
478	At1g18330	EPR1 (EARLY-PHYTOCHROME-RESPONSIVE1); DNA binc	>0.05	0.022	1.021	0.926	0.413	0.591
479	At1g78450	SOUL heme-binding family protein	0.037	0.006	1.074	0.791	0.320	0.366
480	At3g23880	F-box family protein	0.018	0.010	1.339	0.819	0.381	0.596
481	At5g03240	UBQ3 (POLYUBIQUITIN 3); protein binding	0.032	0.019	1.242	0.840	0.442	0.626
482	At5g36910	THI2.2 (THIONIN 2.2); toxin receptor binding	0.035	0.022	1.105	1.064	0.348	0.464

483	At5g66130	ATRAD17 (RADIATION SENSITIVE)	0.037	0.046	1.202	1.019	0.460	0.658
484	At5g52640	HSP81-1 (HEAT SHOCK PROTEIN 81-1); ATP binding / ui	0.042	>0.05	1.115	1.240	0.395	0.598
485	At2g30540	glutaredoxin family protein	0.024	0.039	1.391	1.093	0.400	0.448
486	At1g03620	phagocytosis and cell motility protein ELMO1-related	>0.05	0.022	0.818	0.941	0.450	0.520
487	At1g13670	unknown protein	>0.05	0.007	0.761	0.989	0.407	0.449
488	At1g60740	peroxiredoxin type 2, putative	0.031	0.003	0.817	0.928	0.461	0.449
489	At1g62975	basic helix-loop-helix (bHLH) family protein (bHLH125)	>0.05	0.033	0.835	0.907	0.508	0.484
490	At1g77530	O-methyltransferase family 2 protein	>0.05	0.034	0.951	1.001	0.588	0.468
491	At4g29740	CKX4 (CYTOKININ OXIDASE 4); cytokinin dehydrogenas	>0.05	0.024	0.954	1.063	0.498	0.374
492	At1g78290	serine/threonine protein kinase, putative similar to ser	>0.05	0.038	0.923	1.105	0.477	0.468
493	At2g16005	MD-2-related lipid recognition domain-containing prot	0.031	0.012	0.832	1.220	0.387	0.497
494	At5g17970	disease resistance protein (TIR-NBS-LRR class), putative	0.028	0.001	0.642	0.946	0.490	0.434
495	At4g11320	cysteine proteinase, putative	>0.05	0.003	0.632	0.884	0.490	0.434
496	At5g19530	ACL5 (ACAULIS 5)	>0.05	0.003	0.703	0.906	0.642	0.497
497	At2g38600	acid phosphatase class B family protein	0.040	0.002	0.695	0.810	0.455	0.383
498	At3g23510	cyclopropane fatty acid synthase, putative / CPA-FA syi	0.023	0.001	0.684	0.689	0.331	0.280
499	At2g47180	ATGOLS1 (ARABIDOPSIS THALIANA GALACTINOL SYNTH	>0.05	0.010	0.709	0.807	0.574	0.426
500	At3g59480	pfkB-type carbohydrate kinase family protein	>0.05	0.012	0.717	0.774	0.603	0.408
501	At1g19050	ARR7 (RESPONSE REGULATOR 7); transcription regulato	>0.05	0.002	0.816	0.845	0.620	0.489
502	At5g63600	flavonol synthase, putative	>0.05	0.010	0.664	1.042	0.606	0.493
503	At1g11450	nodulin MtN21 family protein	0.048	0.004	0.617	1.047	0.598	0.435
504	At4g31730	GDU1 (GLUTAMINE DUMPER 1)	>0.05	0.038	0.751	1.066	0.585	0.499
505	At5g26280	meprin and TRAF homology domain-containing proteir	>0.05	0.003	0.425	0.952	0.443	0.417
506	At2g28970	leucine-rich repeat protein kinase, putative	>0.05	0.014	0.433	0.964	0.417	0.464
507	At5g51790	basix helix-loop-helix (bHLH) family protein	>0.05	0.020	0.479	0.886	0.449	0.440
508	At5g62920	ARR6 (RESPONSE REGULATOR 6); transcription regulato	>0.05	0.001	0.535	0.829	0.520	0.419
509	At1g12010	1-aminocyclopropane-1-carboxylate oxidase, putative ,	>0.05	0.003	0.346	0.682	0.411	0.305
510	At1g21100	O-methyltransferase, putative	>0.05	0.003	0.522	0.803	0.550	0.484
511	At1g31320	LBD4 (LOB DOMAIN-CONTAINING PROTEIN 4)	>0.05	0.006	0.661	0.890	0.807	0.492
512	At5g62630	HIPL2 (HIPL2 PROTEIN PRECURSOR); catalytic	0.035	0.004	0.807	0.966	0.735	0.495
513	At2g40330	Bet v I allergen family protein	>0.05	0.009	0.779	0.945	0.763	0.459
514	At1g58370	RXF12; hydrolase, hydrolyzing O-glycosyl compounds	>0.05	0.020	0.886	0.719	0.595	0.464
515	At3g14770	nodulin MtN3 family protein	>0.05	0.019	0.886	0.622	0.471	0.504
516	At3g15250	similar to unknown protein [Arabidopsis thaliana] (TAII	>0.05	0.027	0.864	0.675	0.520	0.493
517	At2g01800	COP1-interacting protein-related	>0.05	0.036	0.843	0.609	0.662	0.454
518	At2g41990	similar to unknown protein [Arabidopsis thaliana] (TAII	>0.05	0.040	0.903	0.656	0.801	0.477
519	At5g14130	peroxidase, putative	0.033	0.006	0.578	0.925	0.964	0.472
520	At1g70880	Bet v I allergen family protein	>0.05	0.022	0.556	1.080	0.700	0.429
521	At1g72360	ethylene-responsive element-binding protein, putative	>0.05	0.036	0.547	1.185	0.807	0.417
522	At1g15380	lactoylglutathione lyase family protein / glyoxalase I fa	>0.05	0.023	0.462	1.025	0.679	0.474
523	At5g33360	transposable element gene	>0.05	0.045	0.493	0.960	0.894	0.633
524	At5g43520	DC1 domain-containing protein	>0.05	0.010	0.468	0.680	0.783	0.373
525	At4g08160	glycosyl hydrolase family 10 protein / carbohydrate-bir	>0.05	0.012	0.493	0.763	0.886	0.500
526	At1g07550	leucine-rich repeat protein kinase, putative	>0.05	0.010	0.553	0.597	0.800	0.443
527	At2g19060	GDSL-motif lipase/hydrolase family protein	>0.05	0.007	0.528	0.554	0.843	0.418
528	At5g44610	MAP18 (MICROTUBULE-ASSOCIATED PROTEIN 18); mic	>0.05	0.002	0.405	0.580	0.724	0.497
529	At1g60140	ATTPS10 (TREHALOSE PHOSPHATE SYNTHASE); transfe	>0.05	0.002	0.415	0.595	0.761	0.524
530	At2g39530	integral membrane protein, putative	>0.05	0.002	0.399	0.637	0.842	0.543
531	At3g13435	unknown protein	>0.05	0.005	0.354	0.607	0.869	0.430

532	At4g18340	glycosyl hydrolase family 17 protein	>0.05	0.002	0.354	0.648	0.781	0.450
533	At2g44390	DC1 domain-containing protein	>0.05	0.008	0.454	0.629	0.732	0.669
534	At4g11290	peroxidase, putative	>0.05	0.004	0.441	0.592	0.692	0.684
535	At3g20140	CYP705A23 (cytochrome P450, family 705, subfamily A	>0.05	0.029	0.391	0.646	0.708	0.653
536	At4g15300	CYP702A2 (cytochrome P450, family 702, subfamily A,	>0.05	0.023	0.479	0.706	0.821	0.758
537	At3g14260	similar to unknown protein [Arabidopsis thaliana] (TAII	>0.05	0.038	0.471	0.612	0.866	0.640
538	At4g07820	pathogenesis-related protein, putative	>0.05	0.006	0.386	0.449	0.852	0.556
539	At4g29180	leucine-rich repeat protein kinase, putative	>0.05	0.032	0.496	0.602	0.822	0.788
540	At2g28990	leucine-rich repeat protein kinase, putative	>0.05	0.041	0.478	0.814	0.846	0.716
541	At3g60020	ASK5 (ARABIDOPSIS SKP1-LIKE 5); ubiquitin-protein liga	>0.05	0.024	0.418	0.733	0.766	0.598
542	At4g13130	DC1 domain-containing protein	>0.05	0.024	0.475	0.857	0.787	0.800
543	At5g49770	leucine-rich repeat transmembrane protein kinase, put	0.026	0.024	0.408	0.797	1.431	0.606
544	At1g77145	similar to unknown protein [Arabidopsis thaliana] (TAII	>0.05	0.040	0.475	0.841	1.113	0.760
545	At1g49860	ATGSTF14 (Arabidopsis thaliana Glutathione S-transfer	0.019	0.003	0.232	0.472	1.433	0.421
546	At4g15390	transferase family protein	>0.05	0.005	0.282	0.554	0.999	0.512
547	At2g23620	esterase, putative	0.050	0.006	0.399	0.413	1.211	0.432
548	At1g14160	integral membrane family protein	>0.05	0.029	0.489	1.130	0.628	0.834
549	At1g74690	IQD31 (IQ-domain 31); calmodulin binding	0.021	>0.05	1.342	0.487	0.872	1.002
550	At5g15830	ATBZIP3 (ARABIDOPSIS THALIANA BASIC LEUCINE-ZIPPI	0.021	>0.05	0.623	1.716	0.883	0.456
551	At4g23410	TET5 (TETRASPANIN5)	0.025	0.032	0.584	1.383	0.909	0.493
552	At2g01520	MLP328 (MLP-LIKE PROTEIN 328)	0.020	0.012	0.584	1.490	0.633	0.439
553	At4g23420	short-chain dehydrogenase/reductase (SDR) family prc	>0.05	0.038	0.704	1.210	0.746	0.498
554	At5g56060	ATP binding / nucleotide binding / phenylalanine-tRNA	0.018	>0.05	0.876	1.444	0.967	0.476
555	At3g49760	ATBZIP5 (ARABIDOPSIS THALIANA BASIC LEUCINE-ZIPPI	0.024	>0.05	0.681	2.457	0.619	0.509
556	At1g23120	major latex protein-related / MLP-related	0.033	>0.05	0.545	2.164	0.742	0.607
557	At2g29330	TRI (TROPINONE REDUCTASE); oxidoreductase	0.026	>0.05	0.452	2.039	1.067	1.181
558	At3g02885	GASA5 (GAST1 PROTEIN HOMOLOG 5)	0.008	0.010	0.466	1.287	1.054	0.701
559	At1g02920	ATGSTF7 (GLUTATHIONE S-TRANSFERASE 11); glutathic	>0.05	0.007	1.473	3.155	3.041	1.931
560	At2g41350	similar to unknown protein [Arabidopsis thaliana] (TAII	>0.05	0.038	1.275	1.899	2.012	1.471
561	At2g47950	unknown protein	>0.05	0.002	1.463	2.905	3.165	1.660
562	At1g26380	FAD-binding domain-containing protein	>0.05	0.002	1.559	2.455	2.298	1.734
563	At5g27760	hypoxia-responsive family protein	>0.05	0.001	1.391	2.183	2.069	1.528
564	At4g28460	unknown protein	>0.05	0.004	1.692	3.330	2.795	1.927
565	At2g37870	protease inhibitor/seed storage/lipid transfer protein (	>0.05	0.001	1.742	3.044	2.743	1.719
566	At1g43910	AAA-type ATPase family protein	>0.05	0.003	1.701	2.334	2.325	1.630
567	At1g26420	FAD-binding domain-containing protein	>0.05	0.003	1.404	2.699	2.303	1.593
568	At3g44300	NIT2 (NITRILASE 2)	>0.05	0.003	1.403	2.908	2.522	1.718
569	At1g52700	phospholipase/carboxylesterase family protein	0.031	0.001	1.305	2.618	1.956	1.601
570	At3g11580	DNA-binding protein, putative	>0.05	0.000	1.413	3.201	2.412	1.841
571	At5g39130	germin-like protein, putative	>0.05	0.003	1.454	2.790	2.103	1.604
572	At2g32210	similar to unknown protein [Arabidopsis thaliana] (TAII	0.021	0.000	1.358	2.222	1.821	1.404
573	At4g19970	similar to unknown protein [Arabidopsis thaliana] (TAII	>0.05	0.021	1.407	2.337	1.967	1.412
574	At4g26270	phosphofructokinase family protein	>0.05	0.001	1.310	2.032	1.782	1.355
575	At5g06760	late embryogenesis abundant group 1 domain-contain	>0.05	0.004	1.714	2.919	2.068	2.064
576	At5g39110	germin-like protein, putative	>0.05	0.002	1.804	4.205	2.540	2.396
577	At2g23270	similar to unknown protein [Arabidopsis thaliana] (TAII	>0.05	0.001	1.825	4.735	2.962	2.604
578	At2g46680	ATHB-7 (ARABIDOPSIS THALIANA HOMEOBOX 7); trans	>0.05	0.001	1.527	2.496	1.952	1.802
579	At2g20145	Toll-Interleukin-Resistance (TIR) domain-containing prc	0.009	0.000	1.396	2.760	1.830	1.865
580	At5g39160	germin-like protein (GLP2a) (GLP5a)	>0.05	0.001	1.595	3.242	2.036	1.817

581	At5g39190	GLP2A (GERMIN-LIKE PROTEIN 2A); manganese ion bin	>0.05	0.002	1.748	3.635	2.373	1.919
582	At5g52750	heavy-metal-associated domain-containing protein	>0.05	0.001	1.810	2.488	2.287	1.983
583	At1g33030	O-methyltransferase family 2 protein	>0.05	0.003	1.703	2.728	2.590	2.083
584	At5g57220	CYP81F2 (cytochrome P450, family 81, subfamily F, pol	>0.05	0.012	1.593	2.290	2.191	1.764
585	At2g29460	ATGSTU4 (GLUTATHIONE S-TRANSFERASE 22); glutathi	>0.05	0.001	1.844	3.111	2.610	2.531
586	At2g32190	similar to unknown protein [Arabidopsis thaliana] (TAII	>0.05	0.000	1.613	2.344	2.089	2.055
587	At5g26920	calmodulin binding	>0.05	0.002	1.371	2.415	2.439	2.248
588	At5g39180	germin-like protein, putative	>0.05	0.002	1.617	6.602	5.317	4.065
589	At2g43510	ATTI1 (ARABIDOPSIS THALIANA TRYPSIN INHIBITOR PR	>0.05	0.002	1.843	7.454	5.229	4.959
590	At5g44820	similar to unknown protein [Arabidopsis thaliana] (TAII	>0.05	0.024	1.334	2.410	2.047	1.752
591	At4g10500	oxidoreductase, 2OG-Fe(II) oxygenase family protein	>0.05	0.004	1.280	2.772	2.129	1.878
592	At2g43570	chitinase, putative	>0.05	0.002	1.627	3.274	3.093	2.229
593	At5g52390	photoassimilate-responsive protein, putative	>0.05	0.010	1.380	3.253	2.239	3.198
594	At1g48000	MYB112 (myb domain protein 112); DNA binding / trar	>0.05	0.004	1.371	2.446	1.853	2.198
595	At1g66700	PXMT1; S-adenosylmethionine-dependent methyltrans	>0.05	0.006	1.544	3.814	2.631	2.716
596	At1g48325	Expressed protein	0.025	0.001	1.237	2.984	2.114	2.289
597	At5g06080	LBD33 (LOB DOMAIN-CONTAINING PROTEIN 33)	>0.05	0.004	1.665	5.890	3.552	1.786
598	At5g13320	PBS3 (AVRPPHB SUSCEPTIBLE 3)	>0.05	0.019	1.384	2.462	1.953	1.265
599	At1g74045	TET17 (TETRASPANIN16)	>0.05	0.002	1.376	2.666	2.133	1.328
600	At1g57630	disease resistance protein (TIR class), putative	>0.05	0.006	1.311	2.086	1.593	1.287
601	At1g73010	phosphoric monoester hydrolase	>0.05	0.004	1.520	3.123	2.032	1.535
602	At1g17710	phosphoric monoester hydrolase	>0.05	0.002	1.334	2.185	1.654	1.274
603	At5g38910	germin-like protein, putative	>0.05	0.007	2.942	9.495	6.693	1.975
604	At5g40370	glutaredoxin, putative	>0.05	0.001	1.448	2.190	1.617	1.334
605	At1g47960	C/VIF1 (CELL WALL / VACUOLAR INHIBITOR OF FRUCTC	0.020	0.000	1.823	3.233	2.067	1.549
606	At1g65690	harpin-induced protein-related / HIN1-related / harpin	>0.05	0.002	1.396	2.119	1.604	1.334
607	At1g60730	aldo/keto reductase family protein	>0.05	0.000	1.843	3.415	2.392	1.727
608	At3g08860	alanine--glyoxylate aminotransferase, putative / beta-a	>0.05	0.002	2.090	4.086	2.475	2.013
609	At4g01380	plastocyanin-like domain-containing protein	0.039	0.000	1.658	2.691	1.807	1.554
610	At2g31200	ADF6 (ACTIN DEPOLYMERIZING FACTOR 6); actin bindi	>0.05	0.002	1.429	2.016	1.439	1.277
611	At1g17490	similar to unknown protein [Arabidopsis thaliana] (TAII	0.021	0.001	1.317	2.043	1.432	1.286
612	At1g73040	jacalin lectin family protein	>0.05	0.002	1.879	2.795	2.268	1.813
613	At2g18660	EXLB3 (EXPANSIN-LIKE B3 PRECURSOR)	>0.05	0.002	1.920	2.658	2.225	1.452
614	At4g17670	senescence-associated protein-related	0.025	0.000	1.863	2.739	2.212	1.498
615	At1g12200	flavin-containing monooxygenase family protein / FMC	>0.05	0.001	1.664	2.296	2.048	1.407
616	At3g16360	AHP4 (HPT PHOSPHOTRANSMITTER 4); histidine phosp	>0.05	0.002	2.438	3.521	2.587	1.687
617	At1g26410	FAD-binding domain-containing protein	0.029	0.002	1.001	3.342	1.904	1.929
618	At1g60470	ATGOLS4 (ARABIDOPSIS THALIANA GALACTINOL SYNTH	0.046	0.003	1.052	5.105	2.769	2.465
619	At3g60180	uridylate kinase, putative / uridine monophosphate kir	>0.05	0.009	1.061	2.248	1.646	1.504
620	At3g53160	UGT73C7 (UDP-GLUCOSYL TRANSFERASE 73C7); UDP-g	0.035	0.002	1.162	3.023	1.987	1.911
621	At4g37390	YDK1 (YADOKARI 1); indole-3-acetic acid amido synthet	>0.05	0.009	1.040	2.821	1.965	2.002
622	At5g66700	HB53 (homeobox-8); DNA binding / transcription facto	>0.05	0.011	1.089	2.389	1.384	1.654
623	At2g42430	LBD16 (ASYMMETRIC LEAVES2-LIKE18)	>0.05	0.007	1.096	2.305	1.467	1.597
624	At1g60970	clathrin adaptor complex small chain family protein	0.042	0.004	1.076	2.717	1.532	1.581
625	At2g39800	P5CS1 (DELTA1-PYRROLINE-5-CARBOXYLATE SYNTHASE	>0.05	0.014	1.183	2.132	1.426	1.524
626	At3g21720	isocitrate lyase, putative	0.032	0.002	1.217	3.283	1.443	2.079
627	At5g57123	similar to unknown protein [Arabidopsis thaliana] (TAII	>0.05	0.033	1.162	2.142	1.407	1.888
628	At5g13210	similar to unknown protein [Arabidopsis thaliana] (TAII	>0.05	0.002	1.539	2.946	1.515	1.845
629	At4g02280	SUS3; UDP-glycosyltransferase/ sucrose synthase/ tran	>0.05	0.001	1.671	3.796	1.777	2.074

630	At3g48920	AtMYB45 (myb domain protein 45); DNA binding / trar	>0.05	0.003	1.288	2.262	1.372	1.548
631	At5g52760	heavy-metal-associated domain-containing protein	>0.05	0.004	1.817	3.483	1.769	1.983
632	At4g04620	ATG8B (AUTOPHAGY 8B); microtubule binding	>0.05	0.002	1.418	2.823	1.388	1.464
633	At4g25010	nodulin MtN3 family protein	>0.05	0.038	1.617	2.694	1.362	1.530
634	At4g31330	similar to unknown protein [Arabidopsis thaliana] (TAII	0.040	0.002	1.433	2.552	1.273	1.545
635	At2g41730	similar to unknown protein [Arabidopsis thaliana] (TAII	0.035	0.001	2.279	4.180	1.557	2.197
636	At4g12735	similar to unknown protein [Arabidopsis thaliana] (TAII	>0.05	0.004	3.208	7.388	2.092	3.713
637	At5g17220	ATGSTF12 (GLUTATHIONE S-TRANSFERASE 26); glutath	>0.05	0.005	2.258	2.365	1.734	1.724
638	At2g47520	AP2 domain-containing transcription factor, putative	>0.05	0.004	2.593	2.769	1.921	1.717
639	At3g50770	calmodulin-related protein, putative	>0.05	0.002	2.840	2.726	1.949	2.031
640	At4g22880	LDOX (TANNIN DEFICIENT SEED 4)	>0.05	0.009	4.310	3.895	2.599	2.728
641	At4g37370	CYP81D8 (cytochrome P450, family 81, subfamily D, pc	>0.05	0.002	2.971	2.716	1.909	2.243
642	At5g40010	AATP1 (AAA-ATPASE 1); ATP binding / ATPase	>0.05	0.030	1.663	2.077	1.569	1.562
643	At4g35190	similar to unknown protein [Arabidopsis thaliana] (TAII	>0.05	0.005	2.277	3.013	2.088	1.949
644	At5g56630	phosphofructokinase family protein	>0.05	0.002	1.663	2.053	1.630	1.625
645	At1g52855	similar to unknown protein [Arabidopsis thaliana] (TAII	>0.05	0.001	2.276	2.589	2.169	1.918
646	At1g70810	C2 domain-containing protein	>0.05	0.000	1.878	2.009	1.740	1.520
647	At4g14400	ACD6 (ACCELERATED CELL DEATH 6); protein binding	>0.05	0.001	3.599	3.079	3.196	1.680
648	At5g05300	similar to unknown protein [Arabidopsis thaliana] (TAII	0.024	0.001	1.540	2.237	4.185	1.499
649	At1g73800	calmodulin-binding protein similar to calmodulin-bindi	>0.05	0.017	1.320	1.601	2.386	1.239
650	At3g25882	NIMIN-2 (NIM1-INTERACTING 2)	>0.05	0.004	1.497	2.014	3.325	1.353
651	At4g21366	S-locus protein kinase-related	>0.05	0.014	1.315	1.498	2.098	1.314
652	At2g22190	trehalose-phosphatase	>0.05	0.012	1.349	1.395	2.311	1.255
653	At3g62730	similar to unknown protein [Arabidopsis thaliana] (TAII	>0.05	0.047	1.440	1.826	4.077	1.506
654	At1g26390	FAD-binding domain-containing protein	>0.05	0.028	1.231	2.358	3.350	1.383
655	At1g55780	heavy-metal-associated domain-containing protein	>0.05	0.004	1.154	1.877	2.381	1.245
656	At5g66640	LIM domain-containing protein-related	>0.05	0.028	1.108	1.578	2.160	1.162
657	At4g21390	B120; protein kinase/ sugar binding	>0.05	0.034	1.175	1.468	2.079	1.163
658	At5g16200	50S ribosomal protein-related	0.035	0.002	1.131	1.620	2.154	1.288
659	At5g49850	jacalin lectin family protein	>0.05	0.035	1.180	1.846	2.519	1.492
660	At3g52780	ATPAP20/PAP20; acid phosphatase/ protein serine/thr	>0.05	0.000	1.415	2.261	4.411	1.695
661	At5g40000	AAA-type ATPase family protein	>0.05	0.010	1.134	3.824	6.576	2.013
662	At2g18360	hydrolase, alpha/beta fold family protein	>0.05	0.014	1.045	1.602	2.015	1.270
663	At4g25900	aldose 1-epimerase family protein	>0.05	0.011	1.110	1.947	2.377	1.352
664	At5g43650	basic helix-loop-helix (bHLH) family protein	>0.05	0.040	1.127	2.501	4.816	1.760
665	At5g53820	similar to unknown protein [Arabidopsis thaliana] (TAII	0.035	0.000	2.998	4.978	8.179	2.609
666	At3g48840	RNA recognition motif (RRM)-containing protein	>0.05	0.001	1.861	2.456	3.108	1.567
667	At2g30750	CYP71A12 (CYTOCHROME P450, FAMILY 71, SUBFAMIL	>0.05	0.005	2.813	6.868	8.746	3.161
668	At1g77120	ADH1 (ALCOHOL DEHYDROGENASE 1)	>0.05	0.004	1.395	2.192	2.991	1.479
669	At3g11840	U-box domain-containing protein	>0.05	0.004	1.345	1.700	2.096	1.281
670	At3g61990	O-methyltransferase family 3 protein	>0.05	0.041	1.268	1.715	2.061	1.434
671	At5g09290	3'(2'),5'-bisphosphate nucleotidase, putative / inositol	>0.05	0.029	1.028	1.975	2.068	1.204
672	At1g51030	similar to unknown protein [Arabidopsis thaliana] (TAII	>0.05	0.018	1.150	3.112	3.355	1.200
673	At1g13195	zinc finger (C3HC4-type RING finger) family protein	0.045	0.012	1.059	2.862	2.998	1.159
674	At1g64405	unknown protein	0.039	0.007	1.008	1.862	2.275	1.117
675	At2g19990	PR-1-LIKE (PATHOGENESIS-RELATED PROTEIN-1-LIKE)	>0.05	0.012	1.096	2.285	2.874	1.118
676	At4g30140	GDSL-motif lipase/hydrolase family protein	0.047	0.008	1.068	1.939	2.732	1.135
677	At5g19650	ATOFP8/OFP8 (Arabidopsis thaliana ovate family prote	>0.05	0.019	1.229	2.292	2.729	1.090
678	At2g14610	PR1 (PATHOGENESIS-RELATED GENE 1)	>0.05	0.006	1.813	5.347	7.516	1.539

679	At4g30650	hydrophobic protein, putative / low temperature and s	>0.05	0.006	1.229	1.727	2.029	1.133
680	At2g15220	secretory protein, putative	>0.05	0.004	1.412	2.914	3.064	1.286
681	At4g19810	glycosyl hydrolase family 18 protein	>0.05	0.002	1.566	3.770	3.931	1.452
682	At3g18250	contains domain PROKAR_LIPOPROTEIN (PS51257)	>0.05	0.006	1.351	2.938	3.338	1.169
683	At4g33550	lipid binding	0.027	0.002	1.411	2.899	3.193	1.211
684	At3g50550	unknown protein	>0.05	0.023	1.183	2.045	2.158	1.182
685	At1g21312	RNA recognition motif (RRM)-containing protein conta	>0.05	0.025	1.233	2.595	2.483	1.190
686	At4g34210	ASK11 (ARABIDOPSIS SKP1-LIKE 11); ubiquitin-protein l	>0.05	0.005	1.245	2.747	2.491	1.315
687	At5g24770	VSP2 (VEGETATIVE STORAGE PROTEIN 2); acid phospha	>0.05	0.033	1.313	2.006	2.278	1.297
688	At5g36925	similar to unknown protein [Arabidopsis thaliana] (TAII	>0.05	0.006	1.540	2.673	2.829	1.401
689	At1g51000	similar to unknown protein [Arabidopsis thaliana] (TAII	0.036	0.009	1.254	5.485	4.281	0.882
690	At2g18600	RUB1-conjugating enzyme, putative	0.013	0.001	1.207	2.221	2.052	1.025
691	At2g30660	3-hydroxyisobutyryl-coenzyme A hydrolase, putative /	>0.05	0.030	1.257	2.388	2.117	0.991
692	At4g11890	protein kinase family protein	>0.05	0.026	1.349	2.502	2.130	0.982
693	At4g20370	TSF (TWIN SISTER OF FT); phosphatidylethanolamine b	0.033	0.004	1.430	2.930	3.129	1.012
694	At2g14560	similar to unknown protein [Arabidopsis thaliana] (TAII	>0.05	0.000	2.767	4.601	5.859	1.501
695	At3g13950	similar to unknown protein [Arabidopsis thaliana] (TAII	0.050	0.003	1.962	2.316	3.696	1.130
696	At2g14600	transposable element gene	>0.05	0.006	1.812	2.910	3.658	0.940
697	At5g18170	GDH1 (GLUTAMATE DEHYDROGENASE 1); oxidoreducta	>0.05	0.038	1.130	1.355	2.120	0.983
698	At1g66600	WRKY63 (WRKY DNA-binding protein 63); transcription	>0.05	0.019	1.263	2.789	4.401	0.838
699	At1g26400	FAD-binding domain-containing protein	>0.05	0.003	1.235	5.192	2.733	1.744
700	At5g01225	similar to unknown protein [Arabidopsis thaliana] (TAII	0.029	0.002	1.191	2.729	1.858	1.392
701	At5g44565	unknown protein	>0.05	0.003	1.202	2.708	2.068	1.577
702	At1g70130	lectin protein kinase, putative	>0.05	0.041	1.148	2.722	2.302	1.556
703	At1g74590	ATGSTU10 (Arabidopsis thaliana Glutathione S-transfe	>0.05	0.003	1.227	4.862	3.757	1.888
704	At2g42440	LOB domain protein 17 / lateral organ boundaries dom	>0.05	0.010	1.154	5.510	3.875	1.646
705	At4g11655	transmembrane protein, putative	0.043	0.003	1.239	3.860	2.935	1.493
706	At2g43580	chitinase, putative	0.016	0.002	0.944	4.219	2.612	1.487
707	At4g10720	ankyrin repeat family protein	>0.05	0.032	0.989	2.428	1.730	1.300
708	At5g62150	peptidoglycan-binding LysM domain-containing protei	0.041	0.009	1.054	2.451	1.702	1.083
709	At2g14330	transposable element gene	>0.05	0.015	1.042	2.986	2.016	1.176
710	At2g20142	transmembrane receptor	>0.05	0.010	1.178	2.053	1.646	1.091
711	At4g34470	ASK12 (ARABIDOPSIS SKP1-LIKE 12); protein binding / u	>0.05	0.017	1.356	3.269	2.164	1.154
712	At3g12510	similar to At3g12510-like protein [Boechera stricta] (GI	>0.05	0.040	1.226	3.308	2.302	1.116
713	At2g40750	WRKY54 (WRKY DNA-binding protein 54); transcription	>0.05	0.007	1.221	2.425	2.022	1.219
714	At2g48150	ATGPX4 (GLUTATHIONE PEROXIDASE 4); glutathione pe	>0.05	0.015	1.192	2.296	1.815	1.175
715	At3g57260	BGL2 (PATHOGENESIS-RELATED PROTEIN 2); glucan 1,3	>0.05	0.017	1.220	2.595	1.973	1.258
716	At4g12500	protease inhibitor/seed storage/lipid transfer protein (	>0.05	0.022	1.204	2.258	1.701	1.216
717	At3g55890	yippee family protein	>0.05	0.028	1.118	2.304	1.751	1.178
718	At1g56600	ATGOLS2 (ARABIDOPSIS THALIANA GALACTINOL SYNTH	0.019	0.002	1.084	3.478	1.737	1.456
719	At2g29950	ELF4-L1 (ELF4-LIKE 1)	>0.05	0.028	1.112	2.089	1.371	1.285
720	At4g37970	mannitol dehydrogenase, putative	0.043	0.013	1.043	2.986	1.507	1.180
721	At5g01300	phosphatidylethanolamine-binding family protein	0.029	0.005	1.011	2.711	1.600	1.250
722	At5g22555	unknown protein	0.048	0.018	0.834	3.713	3.076	1.204
723	At4g23885	similar to unknown protein [Arabidopsis thaliana] (TAII	>0.05	0.034	0.854	2.021	1.924	1.108
724	At3g44510	similar to esterase/lipase/thioesterase family protein [	0.041	0.007	0.930	2.132	2.189	1.213
725	At2g19190	FRK1 (FLG22-INDUCED RECEPTOR-LIKE KINASE 1); kina	>0.05	0.018	1.004	2.283	2.170	1.021
726	At4g11650	ATOSM34 (OSMOTIN 34)	0.044	0.013	0.961	3.182	2.801	1.077
727	At2g27535	ribosomal protein L10A family protein	0.029	0.018	0.850	2.467	1.724	1.071

728	At4g14060	major latex protein-related / MLP-related	0.024	0.007	0.857	4.991	2.632	1.175
729	At3g24310	MYB305 (myb domain protein 305); DNA binding / trar	0.050	0.049	0.831	2.368	1.836	0.996
730	At3g27070	TOM20-1 (TRANSLOCASE OUTER MEMBRANE 20-1)	0.010	0.003	0.843	2.624	2.123	0.965
731	At5g10760	aspartyl protease family protein	>0.05	0.015	2.044	3.485	2.125	1.041
732	At4g10860	similar to unknown protein [Arabidopsis thaliana] (TAII	>0.05	0.023	2.119	3.615	1.936	1.034
733	At5g19470	ATNUDT24 (Arabidopsis thaliana Nudix hydrolase hom	>0.05	0.008	3.442	4.937	3.109	1.348
734	At1g70800	C2 domain-containing protein	0.048	0.002	1.627	2.108	1.505	1.144
735	At5g42040	RPN12B (REGULATORY PARTICLE NON-ATPASE 12B); pe	>0.05	0.010	1.559	2.290	2.106	1.005
736	At3g63380	calcium-transporting ATPase, plasma membrane-type,	0.040	0.002	1.697	3.159	2.421	1.053
737	At2g23680	stress-responsive protein, putative	>0.05	0.003	1.449	2.064	1.832	1.122
738	ath-MIR395e	miRNA gene Arabidopsis thaliana miR395e stem-loop	>0.05	0.039	1.538	2.322	1.817	0.976
739	At5g46720	similar to unknown protein [Arabidopsis thaliana] (TAII	0.046	0.005	1.181	2.065	1.392	1.119
740	At1g35190	oxidoreductase, 2OG-Fe(II) oxygenase family protein	0.032	0.003	1.158	2.183	1.464	1.153
741	At4g37295	unknown protein	0.019	0.001	1.309	2.434	1.593	1.108
742	At5g02780	In2-1 protein, putative	0.043	0.004	1.303	3.074	1.514	1.274
743	At5g50130	short-chain dehydrogenase/reductase (SDR) family prc	>0.05	0.003	1.734	3.345	1.724	1.321
744	At4g12490	protease inhibitor/seed storage/lipid transfer protein (	>0.05	0.003	1.585	3.667	1.806	1.376
745	At1g17020	SRG1 (SENESCENCE-RELATED GENE 1); oxidoreductase	0.031	0.001	1.680	3.263	1.935	1.313
746	At1g53030	cytochrome c oxidase copper chaperone family proteir	>0.05	0.006	1.319	2.037	1.396	1.040
747	At2g36270	ABI5 (ABA INSENSITIVE 5); DNA binding / transcription	>0.05	0.039	1.310	2.068	1.417	1.077
748	At2g43720	similar to unknown protein [Arabidopsis thaliana] (TAII	0.028	0.002	1.505	2.359	1.510	1.076
749	At1g73220	ATOCT1 (ARABIDOPSIS THALIANA ORGANIC CATION/C/	>0.05	0.025	1.543	3.236	1.562	1.181
750	At1g60920	AGL55 (AGAMOUS-LIKE 55); DNA binding / transcriptio	0.019	0.002	1.214	2.027	1.381	1.015
751	At1g05730	similar to unknown protein [Arabidopsis thaliana] (TAII	>0.05	0.018	1.274	2.054	1.418	0.987
752	At4g25380	zinc finger (AN1-like) family protein	0.016	0.003	1.275	3.098	1.826	0.913
753	At2g05500	unknown protein	0.035	0.010	1.134	2.225	1.343	1.011
754	At5g26690	heavy-metal-associated domain-containing protein	0.028	0.008	1.214	2.123	1.145	1.028
755	At2g03250	EXS family protein / ERD1/XPR1/SYG1 family protein	>0.05	0.026	1.221	2.160	1.232	1.031
756	At1g67640	lysine and histidine specific transporter, putative	>0.05	0.037	1.280	2.148	1.125	1.025
757	At3g24780	similar to unknown protein [Arabidopsis thaliana] (TAII	>0.05	0.021	1.318	2.103	1.093	1.042
758	At5g48410	ATGLR1.3 (Arabidopsis thaliana glutamate receptor 1.3	0.028	0.004	1.331	2.440	1.178	1.172
759	At3g30725	ATGDU6 (ARABIDOPSIS THALIANA GLUTAMINE DUMPE	0.019	0.002	1.384	3.368	1.354	1.292
760	At2g20380	kelch repeat-containing F-box family protein	>0.05	0.024	1.910	3.096	1.385	1.168
761	At4g16310	LDL3 (LSD1-LIKE3); amine oxidase	>0.05	0.049	1.728	3.198	1.380	1.121
762	At5g24640	similar to unknown protein [Arabidopsis thaliana] (TAII	>0.05	0.007	2.529	6.017	1.202	1.831
763	At3g28210	PMZ; zinc ion binding	>0.05	0.007	1.472	2.135	1.171	1.207
764	At1g48605	ATHAL3B (Arabidopsis thaliana Hal3-like protein B); ele	0.033	0.002	1.755	2.373	1.191	1.379
765	At1g51090	heavy-metal-associated domain-containing protein	>0.05	0.038	1.937	2.434	1.207	1.204
766	At1g75280	isoflavone reductase, putative	>0.05	0.010	2.456	2.789	1.188	1.430
767	At3g54530	similar to unknown protein [Arabidopsis thaliana] (TAII	>0.05	0.007	2.331	3.624	1.114	1.441
768	At4g16146	similar to unknown protein [Arabidopsis thaliana] (TAII	>0.05	0.013	1.958	2.501	0.993	1.271
769	At2g18860	syntaxin family protein	0.009	0.001	1.778	2.142	1.049	1.033
770	At2g38465	similar to unnamed protein product [Vitis vinifera] (GB	>0.05	0.014	1.751	2.444	1.101	1.089
771	At5g42800	DFR (DIHYDROFLAVONOL 4-REDUCTASE); dihydrokaem	>0.05	0.002	4.408	5.150	2.508	1.731
772	At4g37290	similar to unknown protein [Arabidopsis thaliana] (TAII	>0.05	0.021	2.380	3.148	1.767	1.413
773	At4g28703	similar to unknown protein [Arabidopsis thaliana] (TAII	>0.05	0.001	1.880	2.308	1.433	1.398
774	At5g50800	nodulin MtN3 family protein	>0.05	0.005	2.046	2.553	1.487	1.180
775	At3g05400	sugar transporter, putative	>0.05	0.002	2.362	3.499	1.508	1.556
776	At4g23990	ATCSLG3 (Cellulose synthase-like G3); transferase/ trar	>0.05	0.003	2.329	3.201	1.499	1.376

777	At3g60140	DIN2 (DARK INDUCIBLE 2); hydrolase, hydrolyzing O-gly	>0.05	0.007	2.156	3.184	1.564	1.392
778	At4g21920	unknown protein	>0.05	0.007	1.571	2.030	1.364	1.193
779	At5g26220	ChaC-like family protein	>0.05	0.012	2.267	2.702	1.402	1.859
780	At3g60420	similar to unknown protein [Arabidopsis thaliana] (TAII	>0.05	0.009	2.334	2.538	1.344	1.985
781	At5g48850	male sterility MS5 family protein	>0.05	0.015	2.379	2.438	1.225	1.760
782	At3g49580	similar to unknown protein [Arabidopsis thaliana] (TAII	>0.05	0.006	2.161	2.149	1.211	1.582
783	At1g66380	MYB114 (myb domain protein 114); DNA binding / trar	>0.05	0.002	2.374	2.656	1.167	2.102
784	At5g54610	ANK (ANKYRIN); protein binding	>0.05	0.014	3.804	3.820	2.047	1.883
785	At4g36880	CP1 (CYSTEINE PROTEINASE1); cysteine-type peptidase	>0.05	0.004	2.581	2.316	1.454	1.701
786	At1g53610	similar to unknown protein [Arabidopsis thaliana] (TAII	>0.05	0.001	2.123	2.041	1.293	1.364
787	At2g44460	glycosyl hydrolase family 1 protein	>0.05	0.006	3.076	3.768	1.509	1.796
788	At1g56650	PAP1 (PRODUCTION OF ANTHOCYANIN PIGMENT 1); D	>0.05	0.005	2.400	3.608	1.090	1.965
789	At2g38823	similar to unknown protein [Arabidopsis thaliana] (TAII	0.028	0.001	2.428	4.816	1.340	2.721
790	At3g01970	WRKY45 (WRKY DNA-binding protein 45); transcription	0.049	0.002	1.649	2.195	1.113	1.728
791	At3g22370	AOX1A (alternative oxidase 1A); alternative oxidase	0.019	0.001	1.596	2.142	1.065	1.655
792	At5g40070	similar to AGL81/EMB3008 (EMBRYO DEFECTIVE 3008)	>0.05	0.018	3.904	2.605	1.700	0.870
793	At5g54060	UF3GT (UDP-GLUCOSE:FLAVONOID 3-O-GLUCOSYLTRA	>0.05	0.029	4.042	3.996	1.401	1.226
794	At3g30590	transposable element gene	>0.05	0.042	3.336	2.440	1.602	1.082
795	At4g22870	leucoanthocyanidin dioxygenase, putative / anthocyan	>0.05	0.003	4.487	3.786	2.079	1.300
796	At3g19620	glycosyl hydrolase family 3 protein	>0.05	0.048	2.001	1.472	1.345	1.237
797	At5g51580	unknown protein	>0.05	0.011	2.244	1.729	2.420	0.973
798	At5g05340	peroxidase, putative	>0.05	0.024	1.205	1.151	2.802	2.649
799	At4g24110	similar to unnamed protein product [Vitis vinifera] (GB	>0.05	0.008	1.171	1.098	2.207	2.055
800	At5g28300	trihelix DNA-binding protein, putative	>0.05	0.018	1.142	1.071	2.300	2.196
801	At2g42610	similar to unknown protein [Arabidopsis thaliana] (TAII	>0.05	0.004	1.142	1.043	2.456	2.405
802	At3g12145	FLR1 (FLOR1); enzyme inhibitor	>0.05	0.011	1.098	1.022	2.226	2.273
803	At5g57240	oxysterol-binding family protein	>0.05	0.022	1.319	0.981	2.447	2.407
804	At2g02010	GAD4 (GLUTAMATE DECARBOXYLASE 4); calmodulin bi	>0.05	0.050	1.271	1.002	1.975	2.134
805	At1g53170	ATERF-8/ATERF8 (ETHYLENE RESPONSE ELEMENT BINI	>0.05	0.004	1.435	1.045	2.418	2.080
806	At1g67860	similar to unknown protein [Arabidopsis thaliana] (TAII	>0.05	0.010	1.304	0.965	2.057	1.789
807	At1g66400	calmodulin-related protein, putative	0.011	0.002	1.078	0.924	2.233	1.933
808	At5g21960	AP2 domain-containing transcription factor, putative	>0.05	0.042	1.224	1.037	2.259	1.814
809	At1g06350	fatty acid desaturase family protein	0.011	0.001	1.215	1.059	5.150	3.127
810	At4g15210	ATBETA-AMY (BETA-AMYLASE); beta-amylase	0.008	0.000	1.494	1.005	8.108	3.808
811	At3g10930	similar to unknown protein [Arabidopsis thaliana] (TAII	0.018	0.002	1.225	1.086	2.527	1.774
812	At1g20620	CAT3 (CATALASE 3); catalase	>0.05	0.020	1.146	1.132	2.520	1.783
813	At4g22470	protease inhibitor/seed storage/lipid transfer protein (	>0.05	0.017	1.154	1.081	2.496	1.692
814	At1g72260	THI2.1 (THIONIN 2.1); toxin receptor binding	>0.05	0.019	1.069	1.202	3.072	2.420
815	At2g44840	ATERF13/EREBP (ETHYLENE-RESPONSIVE ELEMENT BIN	0.042	0.004	1.076	1.212	2.600	2.156
816	At5g02230	haloacid dehalogenase-like hydrolase family protein	>0.05	0.033	0.933	1.175	2.268	2.020
817	At2g18480	mannitol transporter, putative	>0.05	0.024	1.018	1.269	2.390	1.701
818	At2g24600	ankyrin repeat family protein	0.018	0.004	0.871	1.229	2.648	1.746
819	At5g15160	bHLH family protein	0.012	0.003	1.063	0.876	3.015	1.963
820	At4g25470	CBF2 (FREEZING TOLERANCE QTL 4); DNA binding / trai	0.028	0.017	1.099	0.758	3.837	2.188
821	At4g29780	similar to unknown protein [Arabidopsis thaliana] (TAII	0.049	0.041	1.078	0.806	2.537	1.748
822	At2g46510	basic helix-loop-helix (bHLH) family protein	0.026	0.012	1.015	0.953	2.085	1.462
823	At4g36220	FAH1 (FERULATE-5-HYDROXYLASE 1); ferulate 5-hydro›	>0.05	0.042	1.079	0.958	2.139	1.428
824	At3g15210	ATERF-4/ATERF4/ERF4/RAP2.5 (ETHYLENE RESPONSIVI	0.027	0.013	1.155	0.877	2.128	1.468
825	At4g25490	CBF1 (C-REPEAT/DRE BINDING FACTOR 1); DNA binding	0.021	0.005	1.507	0.717	9.732	3.002

826	At3g61820	aspartyl protease family protein	0.035	0.048	1.166	0.758	2.235	1.460
827	At5g15970	KIN2 (COLD-RESPONSIVE 6.6)	>0.05	0.008	1.283	1.170	2.361	1.430
828	At2g41180	sigA-binding protein-related	>0.05	0.009	1.325	1.111	2.274	1.402
829	At3g44860	FAMT (FARNESOIC ACID CARBOXYL-O-METHYLTRANSFI	>0.05	0.009	2.026	1.213	6.297	2.530
830	At4g29190	zinc finger (CCCH-type) family protein	>0.05	0.006	1.277	1.101	2.284	1.538
831	At5g66120	3-dehydroquinate synthase, putative	>0.05	0.010	1.381	1.164	2.102	1.667
832	At2g26440	pectinesterase family protein	>0.05	0.003	1.600	1.337	3.903	2.223
833	At1g19210	AP2 domain-containing transcription factor, putative	>0.05	0.007	1.494	1.209	2.691	1.684
834	At5g51990	CBF4/DREB1D (C- REPEAT-BINDING FACTOR 4); DNA bi	0.021	0.003	1.230	1.194	3.468	1.589
835	At1g59950	aldo/keto reductase, putative	>0.05	0.040	1.393	1.301	2.756	1.558
836	At3g56070	ROC2 (rotamase CyP 2); peptidyl-prolyl cis-trans isome	>0.05	0.006	1.247	1.222	2.034	1.373
837	At2g31880	leucine-rich repeat transmembrane protein kinase, put	0.046	0.003	1.278	1.262	2.386	1.450
838	At3g02840	immediate-early fungal elicitor family protein	>0.05	0.006	1.335	1.685	5.698	2.328
839	At4g34410	AP2 domain-containing transcription factor, putative	>0.05	0.030	1.200	1.366	3.041	1.637
840	At1g74010	strictosidine synthase family protein	>0.05	0.010	1.561	1.359	2.824	1.569
841	At4g14450	Identical to Uncharacterized protein At4g14450, chlorc	>0.05	0.022	2.113	1.589	5.693	1.717
842	At3g13857	unknown protein	0.049	0.004	1.651	1.479	4.285	1.443
843	At3g44870	S-adenosyl-L-methionine:carboxyl methyltransferase fa	>0.05	0.007	1.486	1.497	4.474	1.544
844	At5g67080	MAPKKK19 (Mitogen-activated protein kinase kinase ki	0.030	0.013	1.017	1.112	2.524	1.288
845	At4g37490	CYC1 (CYCLIN 1); cyclin-dependent protein kinase regu	0.034	0.016	1.073	1.103	2.198	1.139
846	At4g28660	PSB28 (PHOTOSYSTEM II REACTION CENTER PSB28 PRC	0.018	0.011	1.328	0.739	0.479	0.748
847	At5g14740	CA2 (BETA CARBONIC ANHYDRASE 2); carbonate dehyc	0.026	0.022	1.233	0.608	0.400	0.837
848	At1g72830	HAP2C (Heme activator protein (yeast) homolog 2C); tr	>0.05	0.002	1.058	2.317	2.394	1.515
849	At3g22235	unknown protein	0.035	0.002	1.068	2.499	2.799	1.557
850	At2g02850	ARPN (PLANTACYANIN); copper ion binding	0.040	0.002	1.096	2.542	2.917	1.701
851	At2g08986	similar to unknown protein [Arabidopsis thaliana] (TAII	>0.05	0.020	1.219	3.421	3.896	1.805
852	At3g62990	similar to unknown protein [Arabidopsis thaliana] (TAII	>0.05	0.004	1.140	2.024	2.011	1.478
853	At3g28510	AAA-type ATPase family protein	0.031	0.002	1.164	3.098	2.972	1.577
854	At3g54150	embryo-abundant protein-related	>0.05	0.005	1.173	3.695	3.958	1.595
855	At1g74080	MYB122 (myb domain protein 122); DNA binding / trar	0.044	0.005	0.903	2.808	2.311	1.723
856	At2g07981	similar to unknown protein [Arabidopsis thaliana] (TAII	>0.05	0.011	0.975	2.758	2.790	1.618
857	At2g39370	similar to unknown protein [Arabidopsis thaliana] (TAII	>0.05	0.009	0.993	2.374	2.220	1.478
858	At2g43590	chitinase, putative	0.035	0.004	0.872	3.508	3.188	1.801
859	At5g39120	germin-like protein, putative	>0.05	0.003	1.028	3.393	2.990	2.709
860	At3g04070	ANAC047 (Arabidopsis NAC domain containing protein	>0.05	0.002	1.099	2.266	2.156	2.071
861	At5g39150	germin-like protein, putative	0.045	0.003	0.949	7.051	5.055	3.615
862	At1g13480	similar to unknown protein [Arabidopsis thaliana] (TAII	0.020	0.001	0.999	2.291	2.029	1.817
863	At3g19200	similar to unknown protein [Arabidopsis thaliana] (TAII	0.027	0.002	0.979	2.181	1.800	1.633
864	At4g23700	ATCHX17 (CATION/H+ EXCHANGER 17); monovalent ca	>0.05	0.004	1.092	2.663	2.166	1.946
865	At5g42830	transferase family protein	>0.05	0.005	1.029	2.718	2.804	1.919
866	At1g09932	phosphoglycerate/bisphosphoglycerate mutase-relatec	>0.05	0.004	0.996	2.068	2.137	1.612
867	At1g69920	ATGSTU12 (Arabidopsis thaliana Glutathione S-transfei	0.046	0.002	1.055	2.475	2.324	1.822
868	At5g18470	curculin-like (mannose-binding) lectin family protein	>0.05	0.006	1.283	1.818	3.796	2.241
869	At3g28930	AIG2 (AVRRPT2-INDUCED GENE 2)	>0.05	0.004	1.230	1.632	2.880	1.775
870	At5g46295	unknown protein	0.032	0.002	1.117	1.301	2.011	1.455
871	At5g64905	PROPEP3 (Elicitor peptide 3 precursor)	0.043	0.002	1.064	1.643	2.351	1.619
872	At1g08430	ALMT1/ATALMT1 (AL-ACTIVATED MALATE TRANSPORT	>0.05	0.007	1.186	2.413	5.117	2.397
873	At2g39510	nodulin MtN21 family protein	>0.05	0.013	1.200	2.232	4.633	2.246
874	At2g39350	ABC transporter family protein	>0.05	0.007	1.106	1.759	2.838	1.687

875	At5g65300	unknown protein	0.027	0.003	1.158	1.491	3.023	1.353
876	At1g58420	similar to unknown protein [Arabidopsis thaliana] (TAII	>0.05	0.007	1.046	1.383	2.435	1.438
877	At3g23250	AtMYB15/AtY19/MYB15 (myb domain protein 15); DN/	>0.05	0.007	1.116	1.932	5.802	1.994
878	At1g59730	ATH7 (thioredoxin H-type 7); thiol-disulfide exchange i	0.028	0.004	0.973	1.540	2.522	1.355
879	At3g47540	chitinase, putative	>0.05	0.008	0.955	1.459	2.129	1.348
880	At1g24909	anthranilate synthase beta subunit, putative	0.031	0.004	0.981	1.459	2.040	1.321
881	At3g13650	disease resistance response	>0.05	0.011	1.016	1.485	2.373	1.440
882	At4g01360	similar to BPS1 (BYPASS 1) [Arabidopsis thaliana] (TAIR	>0.05	0.018	1.005	1.597	2.685	1.505
883	At1g25155	anthranilate synthase beta subunit, putative	0.027	0.003	0.949	1.375	2.041	1.393
884	At5g22500	acyl CoA reductase, putative / male-sterility protein, pı	0.045	0.012	0.800	1.743	2.231	1.707
885	At3g12500	ATHCHIB (BASIC CHITINASE); chitinase	>0.05	0.014	0.798	2.113	3.061	2.051
886	At1g30135	JAZ8/TIFY5A (JASMONATE-ZIM-DOMAIN PROTEIN 8)	>0.05	0.007	0.964	2.186	3.365	1.838
887	At5g59490	haloacid dehalogenase-like hydrolase family protein	>0.05	0.021	0.931	1.660	2.437	1.506
888	At1g69930	ATGSTU11 (Arabidopsis thaliana Glutathione S-transfeı	>0.05	0.017	0.871	1.777	2.575	1.597
889	At2g38860	YLS5 (yellow-leaf-specific gene 5)	0.022	0.001	0.871	2.226	3.393	1.935
890	At5g25460	similar to unknown protein [Arabidopsis thaliana] (TAII	>0.05	0.018	0.889	1.378	2.010	1.494
891	At3g45960	ATEXLA3 (ARABIDOPSIS THALIANA EXPANSIN-LIKE A3)	0.035	0.008	0.805	1.633	2.887	1.768
892	At5g57510	unknown protein	>0.05	0.022	0.818	1.512	2.146	1.787
893	At5g53990	glycosyltransferase family protein	>0.05	0.026	0.889	2.122	2.247	1.809
894	At4g13420	HAK5 (High affinity K+ transporter 5); potassium ion tra	>0.05	0.044	0.880	2.404	2.472	2.225
895	At3g53150	UGT73D1 (UDP-GLUCOSYL TRANSFERASE 73D1); UDP-ε	>0.05	0.012	0.968	2.238	2.301	2.273
896	At2g43660	glycosyl hydrolase family protein 17	>0.05	0.002	0.981	1.871	2.175	1.863
897	At3g26830	PAD3 (PHYTOALEXIN DEFICIENT 3); oxygen binding	>0.05	0.036	1.036	2.678	3.846	2.522
898	At4g20000	VQ motif-containing protein	>0.05	0.004	0.991	1.898	2.689	2.044
899	At3g48580	xyloglucan:xyloglucosyl transferase, putative / xylogluc	0.027	0.002	0.912	2.308	2.908	1.966
900	At5g25440	protein kinase family protein	0.031	0.006	0.832	1.889	2.686	1.317
901	At4g08780	peroxidase, putative	>0.05	0.020	0.669	2.511	4.670	1.812
902	At4g14630	GLP9 (GERMIN-LIKE PROTEIN 9); manganese ion bindir	0.024	0.011	0.722	1.923	2.741	1.379
903	At5g45090	ATPP2-A7 (Phloem protein 2-A7); transmembrane rece	0.035	0.008	0.811	1.894	2.198	1.467
904	At1g35910	trehalose-6-phosphate phosphatase, putative	0.035	0.007	0.708	2.807	4.460	1.861
905	At3g48850	mitochondrial phosphate transporter, putative	0.021	0.005	0.777	1.880	2.163	1.352
906	At5g49630	AAP6 (AMINO ACID PERMEASE 6); amino acid transme	>0.05	0.008	0.927	2.140	2.898	1.563
907	At3g09950	similar to unknown protein [Arabidopsis thaliana] (TAII	0.017	0.002	0.919	2.145	2.837	1.329
908	At2g18780	F-box family protein	>0.05	0.049	0.994	1.443	2.017	1.077
909	At3g04720	PR4 (PATHOGENESIS-RELATED 4)	>0.05	0.018	0.891	1.792	2.881	1.255
910	At4g37710	VQ motif-containing protein	>0.05	0.033	0.898	1.694	3.119	1.325
911	At5g38700	similar to unknown protein [Arabidopsis thaliana] (TAII	>0.05	0.037	0.757	1.498	2.031	1.426
912	At4g08770	peroxidase, putative	0.048	0.042	0.611	1.681	2.821	1.840
913	At3g13610	oxidoreductase, 2OG-Fe(II) oxygenase family protein	0.050	0.026	0.778	1.566	2.381	1.446
914	At3g54640	TSA1 (TRYPTOPHAN SYNTHASE ALPHA CHAIN); tryptop	0.023	0.010	0.773	1.515	2.593	1.388
915	At4g19980	unknown protein	0.033	0.041	0.551	1.630	3.905	1.575
916	At2g46650	B5 #1 (cytochrome b5 family protein #1); heme binding	0.018	0.011	0.694	1.308	2.097	1.823
917	At5g06720	peroxidase, putative	>0.05	0.019	1.028	1.388	2.009	2.266
918	At1g52040	MBP1 (MYROSINASE-BINDING PROTEIN 1)	0.041	0.002	1.252	2.410	9.920	7.977
919	At2g26530	AR781	>0.05	0.007	1.185	1.204	1.739	2.042
920	At4g30180	transcription factor/ transcription regulator	>0.05	0.007	1.206	1.282	2.183	3.094
921	At4g35720	similar to unknown protein [Arabidopsis thaliana] (TAII	>0.05	0.002	1.388	1.595	2.324	3.617
922	At5g20190	binding	>0.05	0.006	1.689	1.288	2.113	1.839
923	At5g45350	proline-rich family protein	>0.05	0.002	1.585	1.338	2.095	1.650

924	At2g34810	FAD-binding domain-containing protein	>0.05	0.026	1.514	1.323	2.008	1.861
925	At1g17745	PGDH (3-PHOSPHOGLYCERATE DEHYDROGENASE); pho	>0.05	0.000	1.562	1.420	2.237	2.018
926	At3g45140	LOX2 (LIPOXYGENASE 2)	>0.05	0.007	2.238	1.946	5.202	4.258
927	At4g27654	unknown protein	>0.05	0.004	1.472	1.351	2.196	1.790
928	At5g53030	similar to unknown protein [Arabidopsis thaliana] (TAII	>0.05	0.001	1.593	1.504	2.124	2.028
929	At3g55130	ATWBC19 (WHITE-BROWN COMPLEX HOMOLOG 19); /	>0.05	0.004	1.949	1.568	2.619	2.695
930	At4g25480	DREB1A (DEHYDRATION RESPONSE ELEMENT B1A); DN	>0.05	0.002	2.513	1.860	3.135	3.526
931	At5g42380	CML37/CML39; calcium ion binding	>0.05	0.003	1.557	1.743	2.896	2.630
932	At1g52890	ANAC019 (Arabidopsis NAC domain containing protein	>0.05	0.002	1.950	2.578	5.860	4.444
933	At1g64360	unknown protein	0.039	0.000	1.900	2.623	5.986	4.792
934	At1g80840	WRKY40 (WRKY DNA-binding protein 40); transcription	>0.05	0.004	1.370	1.443	2.338	2.066
935	At1g60190	armadillo/beta-catenin repeat family protein / U-box d	>0.05	0.004	1.382	1.687	3.260	2.472
936	At4g17470	palmitoyl protein thioesterase family protein	>0.05	0.006	1.412	1.884	4.095	3.074
937	At2g22880	VQ motif-containing protein	>0.05	0.001	1.383	1.534	2.647	2.090
938	At1g75040	PR5 (PATHOGENESIS-RELATED GENE 5)	>0.05	0.002	1.705	1.628	3.568	2.192
939	At2g06050	OPR3 (OPDA-REDUCTASE 3); 12-oxophytodienoate red	>0.05	0.013	1.502	1.485	2.912	1.936
940	At2g26560	PLP2 (PHOSPHOLIPASE A 2A); nutrient reservoir	>0.05	0.005	1.597	1.586	2.988	2.093
941	At5g42530	unknown protein	>0.05	0.005	1.636	1.480	2.732	2.946
942	At3g25760	AOC1 (ALLENE OXIDE CYCLASE 1)	>0.05	0.010	1.452	1.326	2.080	2.261
943	At4g23600	CORI3 (CORONATINE INDUCED 1, JASMONIC ACID RESI	>0.05	0.000	2.235	1.941	5.619	5.723
944	At4g14365	zinc finger (C3HC4-type RING finger) family protein / ar	>0.05	0.005	1.597	1.602	2.740	3.198
945	At2g41090	calmodulin-like calcium-binding protein, 22 kDa (CaBP-	>0.05	0.002	2.008	1.533	2.816	3.418
946	At1g24020	MLP423 (MLP-LIKE PROTEIN 423)	>0.05	0.004	1.539	1.404	2.060	2.519
947	At2g02990	RNS1 (RIBONUCLEASE 1); endoribonuclease	>0.05	0.001	2.006	2.785	4.719	6.934
948	At2g38530	LTP2 (LIPID TRANSFER PROTEIN 2); lipid binding	>0.05	0.002	1.603	2.168	4.017	5.000
949	At5g22250	CCR4-NOT transcription complex protein, putative	>0.05	0.012	1.241	1.162	2.160	1.649
950	At2g39030	GCN5-related N-acetyltransferase (GNAT) family protei	>0.05	0.004	1.676	1.691	6.013	3.459
951	At1g61340	F-box family protein	>0.05	0.004	1.372	1.438	4.206	3.347
952	At2g15050	LTP; lipid binding	0.032	0.000	1.400	1.180	3.144	2.256
953	At2g22500	mitochondrial substrate carrier family protein	>0.05	0.007	1.332	1.209	3.523	2.547
954	At5g36220	CYP81D1 (CYTOCHROME P450 91A1); oxygen binding	>0.05	0.005	1.531	1.510	2.925	2.749
955	At4g08870	arginase, putative	>0.05	0.008	1.565	1.402	3.023	2.678
956	At4g08170	inositol 1,3,4-trisphosphate 5/6-kinase family protein	>0.05	0.009	1.279	1.391	2.292	1.997
957	At4g23810	WRKY53 (WRKY DNA-binding protein 53); DNA binding	>0.05	0.006	1.287	1.304	2.232	1.974
958	At1g76640	calmodulin-related protein, putative	>0.05	0.007	1.473	1.242	2.835	2.323
959	At1g78490	CYP708A3 (cytochrome P450, family 708, subfamily A,	>0.05	0.005	1.409	1.273	2.730	2.276
960	At5g22580	Identical to Uncharacterized protein At5g22580 [Arabi	0.036	0.002	1.227	1.157	2.105	2.149
961	At5g25610	RD22 (RESPONSIVE TO DESSICATION 22)	>0.05	0.008	1.299	1.199	2.377	2.391
962	At2g41230	unknown protein	>0.05	0.005	1.286	1.198	2.266	2.253
963	At1g54020	myrosinase-associated protein, putative	0.039	0.000	1.451	1.312	3.752	3.451
964	At5g37600	ATGSR1 (Arabidopsis thaliana glutamine synthase clon	>0.05	0.012	1.273	1.120	2.064	2.023
965	At3g50760	GATL2 (Galacturonosyltransferase-like 2); polygalactur	>0.05	0.024	1.394	1.216	2.238	2.544
966	At1g17420	LOX3 (Lipoxygenase 3); iron ion binding / lipoxygenase	>0.05	0.012	1.654	1.120	2.498	1.929
967	At5g06730	peroxidase, putative	>0.05	0.002	1.450	2.803	5.665	3.226
968	At5g22460	esterase/lipase/thioesterase family protein	>0.05	0.002	1.463	2.484	4.984	3.720
969	At1g61380	S-locus protein kinase, putative	>0.05	0.006	1.186	1.518	2.058	1.788
970	At2g47190	MYB2 (myb domain protein 2); DNA binding / transcrip	>0.05	0.002	1.380	1.944	3.050	2.379
971	At5g64810	WRKY51 (WRKY DNA-binding protein 51); transcription	>0.05	0.011	1.516	2.762	4.553	2.465
972	At3g50900	similar to unknown protein [Arabidopsis thaliana] (TAII	>0.05	0.006	1.189	1.734	2.108	1.665

973	At1g19960	similar to transmembrane receptor [Arabidopsis thaliar	>0.05	0.001	1.542	2.664	4.012	2.687
974	At2g46400	WRKY46 (WRKY DNA-binding protein 46); transcription	>0.05	0.004	1.246	2.298	2.916	2.363
975	At4g15440	HPL1 (HYDROPEROXIDE LYASE 1); heme binding / iron i	>0.05	0.011	1.229	1.771	2.184	1.860
976	At1g24575	unknown protein	>0.05	0.004	1.167	1.855	2.384	2.009
977	At5g59320	LTP3 (LIPID TRANSFER PROTEIN 3); lipid binding	>0.05	0.002	2.366	5.826	9.914	5.657
978	At4g39670	glycolipid binding / glycolipid transporter	>0.05	0.006	1.350	1.963	2.296	1.894
979	At1g24145	unknown protein	>0.05	0.011	1.311	1.754	2.010	1.653
980	At1g51420	sucrose-phosphatase, putative	>0.05	0.012	1.310	1.922	2.103	1.693
981	At2g30770	CYP71A13 (CYTOCHROME P450, FAMILY 71, SUBFAMIL	>0.05	0.006	1.825	3.056	4.278	2.468
982	At4g01390	meprin and TRAF homology domain-containing proteir	>0.05	0.004	1.370	1.989	2.270	1.660
983	At1g28480	GRX480; thiol-disulfide exchange intermediate	>0.05	0.001	1.520	1.693	2.633	1.836
984	At1g61800	GPT2 (glucose-6-phosphate/phosphate translocator 2)	>0.05	0.002	2.334	2.560	5.597	3.008
985	At4g28490	HAESA (RECEPTOR-LIKE PROTEIN KINASE 5); ATP bindir	>0.05	0.038	1.471	1.477	2.188	1.645
986	At5g66650	similar to unknown protein [Arabidopsis thaliana] (TAII	>0.05	0.002	1.895	1.917	3.150	2.081
987	At3g57700	protein kinase, putative	>0.05	0.015	1.725	1.588	2.560	1.755
988	At5g39670	calcium-binding EF hand family protein	>0.05	0.002	1.646	2.213	3.312	2.376
989	At1g11210	similar to unknown protein [Arabidopsis thaliana] (TAII	>0.05	0.001	1.646	1.840	2.627	2.057
990	At5g25250	similar to unknown protein [Arabidopsis thaliana] (TAII	>0.05	0.008	1.355	1.508	2.377	1.509
991	At4g36500	similar to unknown protein [Arabidopsis thaliana] (TAII	>0.05	0.002	1.383	1.476	2.186	1.450
992	At2g14290	F-box family protein	>0.05	0.003	1.466	1.708	2.734	1.750
993	At1g56580	similar to unknown protein [Arabidopsis thaliana] (TAII	>0.05	0.004	1.178	1.435	2.071	1.402
994	At3g49550	similar to unknown protein [Arabidopsis thaliana] (TAII	>0.05	0.005	1.227	1.564	2.212	1.500
995	At3g51895	SULTR3;1 (SULFATE TRANSPORTER 1); sulfate transmer	>0.05	0.018	1.312	1.622	2.646	1.643
996	At5g06860	PGIP1 (POLYGALACTURONASE INHIBITING PROTEIN 1);	>0.05	0.004	1.321	1.967	1.783	2.583
997	At1g68290	ENDO 2 (ENDONUCLEASE 2); T/G mismatch-specific en	>0.05	0.005	1.250	1.559	1.512	2.068
998	At5g09800	U-box domain-containing protein	>0.05	0.005	1.358	1.852	2.034	2.236
999	At2g43530	trypsin inhibitor, putative	>0.05	0.002	1.548	2.146	2.347	2.940
1000	At1g56240	ATPP2-B13 (Phloem protein 2-B13); carbohydrate bind	>0.05	0.011	1.436	1.636	1.975	2.220
1001	At4g26950	similar to unknown protein [Arabidopsis thaliana] (TAII	>0.05	0.002	2.098	2.215	3.050	3.753
1002	At1g80130	binding	>0.05	0.003	1.920	2.381	2.950	4.498
1003	At2g25625	similar to unnamed protein product [Vitis vinifera] (GB	>0.05	0.000	2.200	2.927	3.293	5.829
1004	At5g47220	ATERF-2/ATERF2/ERF2 (ETHYLENE RESPONSE FACTOR 2	>0.05	0.004	1.453	1.414	1.713	2.137
1005	At1g20990	DC1 domain-containing protein	>0.05	0.010	1.887	1.870	2.350	3.348
1006	At3g26180	CYP71B20 (cytochrome P450, family 71, subfamily B, p	>0.05	0.006	1.503	1.354	1.544	2.001
1007	At1g02930	ATGSTF6 (EARLY RESPONSIVE TO DEHYDRATION 11); gl	>0.05	0.004	1.942	3.289	3.494	3.230
1008	At1g76960	unknown protein	>0.05	0.002	1.555	2.193	2.485	2.315
1009	At1g32350	AOX1D (ALTERNATIVE OXIDASE 1D); alternative oxidas	>0.05	0.001	1.944	2.775	3.576	2.872
1010	At5g59220	protein phosphatase 2C, putative / PP2C, putative	>0.05	0.002	1.451	2.206	2.216	2.324
1011	At2g28500	LBD11 (LOB DOMAIN-CONTAINING PROTEIN 11)	>0.05	0.036	1.385	1.988	1.884	2.069
1012	At5g38900	DSBA oxidoreductase family protein	>0.05	0.004	2.341	3.311	3.872	3.240
1013	At4g33040	glutaredoxin family protein	>0.05	0.004	1.666	1.942	2.180	2.099
1014	At3g47480	calcium-binding EF hand family protein	>0.05	0.003	2.683	3.296	3.872	3.173
1015	At1g65500	similar to unknown protein [Arabidopsis thaliana] (TAII	>0.05	0.021	1.980	2.249	2.344	2.402
1016	At1g23730	carbonic anhydrase, putative / carbonate dehydratase,	>0.05	0.002	2.683	3.850	3.622	4.439
1017	At2g41380	embryo-abundant protein-related	>0.05	0.002	1.494	1.760	1.711	2.016
1018	At4g23190	CRK11 (CYSTEINE-RICH RLK11); kinase	>0.05	0.001	1.595	1.789	1.931	2.068
1019	At5g47060	senescence-associated protein-related	>0.05	0.001	1.941	2.020	2.772	2.416
1020	At3g25770	AOC2 (ALLENE OXIDE CYCLASE 2)	>0.05	0.003	3.011	3.104	5.006	4.611
1021	At2g29350	SAG13 (Senescence-associated gene 13); oxidoreducta	>0.05	0.001	3.033	4.288	8.049	6.565

1022	At2g29470	ATGSTU3 (GLUTATHIONE S-TRANSFERASE 21); glutathi	>0.05	0.004	2.638	3.750	6.978	6.061
1023	At3g23240	ATERF1/ERF1 (ETHYLENE RESPONSE FACTOR 1); DNA b	>0.05	0.001	1.749	2.129	2.773	2.845
1024	At5g39030	protein kinase family protein	>0.05	0.001	1.361	2.364	2.954	2.621
1025	At5g39520	similar to unknown protein [Arabidopsis thaliana] (TAII	>0.05	0.016	1.455	2.581	3.000	3.020
1026	At1g47510	endonuclease/exonuclease/phosphatase family protei	>0.05	0.004	1.359	2.052	2.514	2.683
1027	At1g69880	ATH8 (thioredoxin H-type 8); thiol-disulfide exchange i	>0.05	0.003	1.818	3.262	5.042	5.149
1028	At3g28580	AAA-type ATPase family protein	>0.05	0.014	1.214	1.681	2.029	2.052
1029	At4g22710	CYP706A2 (cytochrome P450, family 706, subfamily A,	>0.05	0.010	1.291	1.652	2.086	1.966
1030	At1g52000	jacalin lectin family protein	>0.05	0.005	1.405	2.181	2.759	3.220
1031	At3g50930	AAA-type ATPase family protein	>0.05	0.004	1.256	1.692	1.979	2.216
1032	At2g28400	similar to unknown protein [Arabidopsis thaliana] (TAII	>0.05	0.005	1.212	1.683	2.101	2.329
1033	At4g24380	hydrolase, acting on ester bonds	>0.05	0.002	1.219	1.819	2.170	2.465
1034	At1g63055	similar to unknown protein [Arabidopsis thaliana] (TAII	>0.05	0.006	1.325	3.163	4.014	5.427
1035	At2g23170	GH3.3; indole-3-acetic acid amido synthetase	>0.05	0.004	1.129	2.077	2.560	2.670
1036	At2g44580	protein binding / zinc ion binding	0.034	0.001	1.097	2.529	3.253	2.947
1037	At1g52565	similar to unknown protein [Arabidopsis thaliana] (TAII	0.035	0.002	1.052	1.760	1.643	2.063
1038	At4g38410	dehydrin, putative	>0.05	0.004	1.249	2.892	2.353	4.109
1039	At5g14780	FDH (FORMATE DEHYDROGENASE); NAD binding / binc	>0.05	0.006	1.875	1.472	2.060	1.729
1040	At5g27420	zinc finger (C3HC4-type RING finger) family protein	>0.05	0.001	2.270	1.814	3.031	2.079
1041	At5g63130	octicosapeptide/Phox/Bem1p (PB1) domain-containing	>0.05	0.005	1.825	1.637	2.083	1.985
1042	At4g23170	EP1; protein kinase	>0.05	0.003	1.951	1.620	2.154	2.004
1043	At3g14440	NCED3 (NINE-CIS-EPOXYCAROTENOID DIOXYGENASE3)	>0.05	0.012	1.912	1.311	2.002	1.614
1044	At4g02520	ATGSTF2 (Arabidopsis thaliana Glutathione S-transfera	>0.05	0.008	1.764	1.359	2.459	1.584
1045	At5g24860	FPF1 (FLOWERING PROMOTING FACTOR 1)	>0.05	0.038	3.680	3.848	5.714	2.874
1046	At2g42540	COR15A (COLD-REGULATED 15A)	>0.05	0.000	2.480	2.888	3.697	2.175
1047	At5g03210	unknown protein	>0.05	0.001	3.549	3.332	5.484	3.207
1048	At2g42530	COR15B	>0.05	0.004	2.670	2.355	2.843	1.987
1049	At2g02930	ATGSTF3 (GLUTATHIONE S-TRANSFERASE 16); glutathic	>0.05	0.016	1.656	1.651	2.324	1.397
1050	At4g14020	rapid alkalinization factor (RALF) family protein	>0.05	0.002	1.917	2.006	2.851	1.552
1051	At3g03530	NPC4 (NONSPECIFIC PHOSPHOLIPASE C4); hydrolase, a	>0.05	0.002	2.295	1.967	3.270	1.736
1052	At3g45350	transposable element gene	>0.05	0.007	2.004	1.726	2.894	1.322
1053	At4g35180	LHT7 (LYS/HIS TRANSPORTER 7); amino acid transmem	>0.05	0.013	2.606	1.898	3.605	1.330
1054	At5g08790	ATAF2 (Arabidopsis NAC domain containing protein 81	>0.05	0.002	1.805	1.631	1.280	2.020
1055	At4g09020	ATISA3/ISA3 (ISOAMYLASE 3); alpha-amylase	>0.05	0.045	2.065	1.654	1.362	2.268
1056	At5g22860	serine carboxypeptidase S28 family protein	>0.05	0.002	1.720	1.653	1.434	2.130
1057	At4g12580	unknown protein	>0.05	0.031	1.601	1.584	1.318	2.045
1058	At1g02850	glycosyl hydrolase family 1 protein	>0.05	0.002	2.789	2.431	1.445	2.699
1059	At1g26770	ATEXPA10 (ARABIDOPSIS THALIANA EXPANSIN A10)	>0.05	0.011	2.118	1.414	1.497	2.788
1060	At1g29395	COR414-TM1 (cold regulated 414 thylakoid membrane	>0.05	0.002	1.743	1.556	1.365	2.439
1061	At2g46640	unknown protein	>0.05	0.006	1.589	1.369	1.303	2.223
1062	At1g71140	MATE efflux family protein	>0.05	0.027	2.216	1.432	1.459	2.265
1063	At3g25250	AGC2-1 (OXIDATIVE SIGNAL-INDUCIBLE1); kinase	>0.05	0.001	2.052	1.563	1.500	2.469
1064	At5g17860	CAX7 (CALCIUM EXCHANGER 7); calcium:sodium antipo	>0.05	0.003	2.865	1.807	2.581	3.464
1065	At3g44310	ATNIT1/NIT1/NITI (NITRILASE 1); nitrilase	>0.05	0.008	1.875	1.375	1.641	2.381
1066	At5g17760	AAA-type ATPase family protein	>0.05	0.000	1.997	2.325	1.972	2.418
1067	At5g53420	similar to unknown protein [Arabidopsis thaliana] (TAII	>0.05	0.002	1.803	2.031	1.663	2.126
1068	At1g21520	unknown protein	>0.05	0.001	1.969	2.663	2.035	2.451
1069	At1g54575	unknown protein	>0.05	0.002	2.121	2.499	2.254	2.150
1070	At3g56710	SIB1 (SIGMA FACTOR BINDING PROTEIN 1); binding	>0.05	0.002	2.698	2.443	2.505	2.446

1071	At1g31580	ECS1	>0.05	0.017	1.823	1.736	1.642	2.228
1072	At2g36970	UDP-glucoronosyl/UDP-glucosyl transferase family pro	>0.05	0.003	1.827	1.823	1.845	2.315
1073	ath-MIR157b	miRNA gene Arabidopsis thaliana miR157b stem-loop	>0.05	0.007	1.556	1.621	1.472	2.010
1074	At2g37430	zinc finger (C2H2 type) family protein (ZAT11)	>0.05	0.001	1.855	2.089	1.823	2.627
1075	At5g59310	LTP4 (LIPID TRANSFER PROTEIN 4); lipid binding	>0.05	0.002	2.914	4.971	3.274	6.907
1076	At5g47070	protein kinase, putative	>0.05	0.002	1.467	1.578	1.464	2.044
1077	At1g17180	ATGSTU25 (Arabidopsis thaliana Glutathione S-transfe	>0.05	0.007	1.470	1.703	1.581	2.418
1078	At2g21640	similar to unknown protein [Arabidopsis thaliana] (TAII	>0.05	0.004	1.960	2.405	1.525	2.773
1079	At3g46080	zinc finger (C2H2 type) family protein	>0.05	0.005	1.743	2.215	1.534	2.263
1080	At5g38200	hydrolase	>0.05	0.005	3.008	1.569	2.355	2.087
1081	At1g61550	S-locus protein kinase, putative	>0.05	0.042	4.161	1.777	3.092	3.343
1082	At4g12290	copper amine oxidase, putative	>0.05	0.004	2.723	1.880	2.083	2.557
1083	At1g64950	CYP89A5 (cytochrome P450, family 87, subfamily A, po	>0.05	0.021	2.360	1.624	1.635	1.718
1084	At4g34710	ADC2 (ARGININE DECARBOXYLASE 2)	>0.05	0.047	2.189	1.210	1.483	1.935
1085	At5g54490	PBP1 (PINOID-BINDING PROTEIN 1); calcium ion bindin	0.028	0.003	1.282	1.444	1.194	2.491
1086	At1g72920	disease resistance protein (TIR-NBS class), putative	>0.05	0.007	1.365	1.576	1.343	2.666
1087	At1g02470	similar to unknown protein [Arabidopsis thaliana] (TAII	>0.05	0.006	1.173	1.512	1.294	2.056
1088	At1g34060	alliinase family protein	>0.05	0.043	1.339	1.515	1.061	2.543
1089	At2g33380	RD20 (RESPONSIVE TO DESSICATION 20); calcium ion b	>0.05	0.005	1.827	2.712	1.457	3.834
1090	At2g43500	RWP-RK domain-containing protein	>0.05	0.010	1.457	1.854	1.100	2.439
1091	At3g13130	similar to hypothetical protein [Vitis vinifera] (GB:CAN7	>0.05	0.038	1.432	1.572	1.143	2.221
1092	At1g53870	similar to unknown protein [Arabidopsis thaliana] (TAII	>0.05	0.001	1.751	1.729	1.061	2.323
1093	At1g53890	similar to unknown protein [Arabidopsis thaliana] (TAII	>0.05	0.008	1.866	1.703	1.121	2.257
1094	At1g72900	disease resistance protein (TIR-NBS class), putative	0.025	0.002	2.368	2.167	0.983	2.970
1095	At2g04050	MATE efflux family protein	>0.05	0.004	1.950	1.708	1.049	2.233
1096	At1g59700	ATGSTU16 (Arabidopsis thaliana Glutathione S-transfe	0.042	0.003	1.709	1.591	1.091	2.614
1097	At2g03760	ST (steroid sulfotransferase); sulfotransferase	0.047	0.005	1.668	1.441	0.997	2.197
1098	At1g72910	disease resistance protein (TIR-NBS class), putative	0.040	0.005	1.965	1.746	0.898	2.546
1099	At4g15490	UGT84A3; UDP-glycosyltransferase/ sinapate 1-glucosy	0.048	0.006	1.848	1.580	0.886	2.126
1100	At4g01870	tolB protein-related	>0.05	0.010	3.019	1.384	1.099	2.317
1101	At5g44580	similar to unknown protein [Arabidopsis thaliana] (TAII	>0.05	0.009	1.502	1.157	1.151	2.154
1102	At3g52340	SPP2; sucrose-phosphatase	>0.05	0.008	1.644	1.215	1.309	2.468
1103	At2g20870	cell wall protein precursor, putative	0.050	0.024	1.373	1.091	1.048	2.812
1104	At5g58390	peroxidase, putative	>0.05	0.013	1.828	1.225	2.467	2.801
1105	At4g11530	protein kinase family protein	>0.05	0.004	1.796	1.183	1.873	2.679
1106	At5g01180	proton-dependent oligopeptide transport (POT) family	>0.05	0.009	1.747	1.131	2.082	2.741
1107	At3g18080	glycosyl hydrolase family 1 protein	>0.05	0.005	1.333	1.130	1.511	2.008
1108	At4g23060	IQD22 (IQ-domain 22); calmodulin binding	>0.05	0.007	1.390	1.139	1.589	2.244
1109	At4g37990	ELI3-2 (ELICITOR-ACTIVATED GENE 3)	>0.05	0.007	2.006	0.915	2.263	2.906
1110	At1g72940	disease resistance protein (TIR-NBS class), putative	>0.05	0.025	1.248	1.242	1.352	2.126
1111	At1g75580	auxin-responsive protein, putative	0.025	0.003	1.154	1.098	1.387	2.066
1112	At5g06870	PGIP2 (POLYGALACTURONASE INHIBITING PROTEIN 2);	0.032	0.006	0.912	1.374	2.279	3.729
1113	At2g29120	ATGLR2.7 (Arabidopsis thaliana glutamate receptor 2.7	>0.05	0.008	1.028	1.230	1.637	2.039
1114	At5g50335	unknown protein	0.013	0.002	1.085	0.946	3.330	5.096
1115	At3g55240	similar to unknown protein [Arabidopsis thaliana] (TAII	0.017	0.003	1.005	1.002	3.787	6.332
1116	At1g19670	ATCLH1 (CORONATINE-INDUCED PROTEIN 1)	0.037	0.005	1.188	1.074	2.943	3.787
1117	At4g02330	ATPMEPCRB; pectinesterase	>0.05	0.012	1.174	1.122	3.748	4.996
1118	At2g29440	ATGSTU6 (GLUTATHIONE S-TRANSFERASE 24); glutathi	>0.05	0.022	1.042	1.079	1.545	2.054
1119	At2g47780	rubber elongation factor (REF) protein-related	0.035	0.005	1.067	1.278	3.316	5.843

1120	At2g38540	LP1 (nonspecific lipid transfer protein 1)	>0.05	0.024	1.088	1.124	1.682	2.240
1121	At3g60650	unknown protein	0.032	0.010	0.967	1.163	1.351	2.181
1122	At4g30290	ATXTH19 (XYLOGLUCAN ENDOTRANSGLUCOSYLASE/HY	>0.05	0.046	0.799	1.057	2.174	3.846
1123	At5g12940	leucine-rich repeat family protein	0.019	0.027	0.797	0.869	2.104	1.955
1124	At1g26945	transcription regulator	0.035	0.028	0.795	0.863	4.281	4.043
1125	At1g52400	BGL1 (BETA-GLUCOSIDASE HOMOLOG 1); hydrolase, hy	0.013	0.003	0.754	1.150	7.569	10.173
1126	At1g67750	pectate lyase family protein	0.015	0.004	0.885	1.008	2.437	2.529
1127	At4g32460	similar to unknown protein [Arabidopsis thaliana] (TAII	0.020	>0.05	0.899	0.764	2.311	2.620
1128	At4g36110	auxin-responsive protein, putative	0.012	0.017	0.766	0.744	2.208	3.195
1129	At5g49170	similar to unknown protein [Arabidopsis thaliana] (TAII	0.035	0.043	0.995	0.784	1.636	2.938
1130	At3g14210	ESM1 (EPITHIOSPECIFIER MODIFIER 1); carboxylesteras	0.018	0.024	1.040	0.675	1.866	2.937
1131	At2g41280	M10	0.026	0.008	1.193	0.857	1.870	2.926
1132	At4g24120	YSL1 (YELLOW STRIPE LIKE 1); oligopeptide transporter	0.031	0.014	1.041	0.834	2.096	2.714
1133	At5g15230	GASA4 (GAST1 PROTEIN HOMOLOG 4)	0.011	0.004	1.031	0.954	1.198	2.011
1134	At5g47230	ERF5 (ETHYLENE RESPONSIVE ELEMENT BINDING FACT	0.012	0.003	1.317	0.884	1.219	2.033
1135	At2g39980	transferase family protein	0.033	>0.05	1.151	0.809	1.121	2.104
1136	At1g19610	LCR78/PDF1.4 (Low-molecular-weight cysteine-rich 78)	0.021	0.035	0.992	1.144	0.944	2.118
1137	At5g07990	TT7 (TRANSPARENT TESTA 7); flavonoid 3'-monooxyge	0.008	>0.05	2.108	2.121	0.436	0.969
1138	At5g61950	ubiquitin carboxyl-terminal hydrolase-related	0.012	0.030	2.245	1.729	0.534	1.300
1139	At4g08555	unknown protein	0.019	0.038	2.401	1.932	0.573	1.244
1140	At1g09500	cinnamyl-alcohol dehydrogenase family / CAD family	0.020	>0.05	2.108	2.123	0.493	1.673
1141	At4g19515	disease resistance family protein	0.018	>0.05	1.634	2.086	0.583	1.256
1142	At1g05680	UDP-glucoronosyl/UDP-glucosyl transferase family pro	0.019	0.017	2.086	1.864	0.535	2.486
1143	At2g36750	UGT72C1 (UDP-GLUCOSYL TRANSFERASE 72C1); UDP-g	0.046	0.034	2.770	1.936	0.576	2.707
1144	At2g36780	UDP-glucoronosyl/UDP-glucosyl transferase family pro	>0.05	0.019	3.536	1.824	0.765	2.516
1145	At2g36800	DOGT1 (DON-GLUCOSYLTRANSFERASE); UDP-glycosyltı	0.026	0.006	2.438	1.782	0.738	2.204
1146	At3g28730	ATHMG (HIGH MOBILITY GROUP); transcription factor	0.022	0.041	3.235	1.761	0.433	1.794
1147	At4g31870	ATGPX7 (GLUTATHIONE PEROXIDASE 7); glutathione pe	0.037	>0.05	2.038	1.242	0.683	1.478
1148	At3g28740	cytochrome P450 family protein	0.016	>0.05	2.324	1.196	0.495	1.808
1149	At5g09470	mitochondrial substrate carrier family protein	>0.05	0.049	2.823	2.054	1.220	1.283
1150	At1g75290	isoflavone reductase, putative	0.045	0.004	2.310	2.164	1.048	1.331
1151	At3g55910	unknown protein	>0.05	0.040	2.221	2.076	0.971	1.275
1152	At3g56260	unknown protein	>0.05	0.039	2.043	1.969	0.970	1.292
1153	At5g16970	AT-AER (ALKENAL REDUCTASE); 2-alkenal reductase	>0.05	0.008	2.670	1.951	0.895	1.718
1154	At3g49570	similar to unknown protein [Arabidopsis thaliana] (TAII	0.047	0.007	2.169	1.649	0.903	1.562
1155	At5g17000	NADP-dependent oxidoreductase, putative	0.035	0.005	2.293	1.909	0.869	1.668
1156	At2g37770	aldo/keto reductase family protein	>0.05	0.004	4.624	3.061	1.058	2.885
1157	At3g04000	short-chain dehydrogenase/reductase (SDR) family prc	>0.05	0.004	2.245	2.064	1.055	1.760
1158	At3g14620	CYP72A8 (cytochrome P450, family 72, subfamily A, po	>0.05	0.008	3.434	2.828	1.073	2.213
1159	At3g17790	ATACP5 (acid phosphatase 5); acid phosphatase/ prote	>0.05	0.005	2.570	2.136	1.166	1.710
1160	At5g43450	2-oxoglutarate-dependent dioxygenase, putative	0.025	0.004	2.194	2.795	0.801	1.843
1161	At3g02480	ABA-responsive protein-related	>0.05	0.004	2.284	2.637	0.951	1.981
1162	At5g13370	auxin-responsive GH3 family protein	0.030	0.007	1.867	2.387	0.878	1.261
1163	At5g20150	SPX (SYG1/Pho81/XPR1) domain-containing protein	0.013	0.008	1.995	2.406	0.712	1.147
1164	At3g09390	MT2A (METALLOTHIONEIN 2A)	0.019	0.010	2.221	2.025	0.766	1.145
1165	At5g16980	NADP-dependent oxidoreductase, putative	>0.05	0.036	2.422	1.757	0.750	1.472
1166	At5g58570	unknown protein	0.021	0.013	2.040	1.736	0.710	1.397
1167	At5g24660	similar to unknown protein [Arabidopsis thaliana] (TAII	>0.05	0.021	2.694	2.327	0.769	1.543
1168	At2g44130	kelch repeat-containing F-box family protein	0.020	0.022	2.718	1.371	0.779	1.102

1169	At4g26280	sulfotransferase family protein	>0.05	0.034	30.387	5.621	0.789	0.906
1170	At4g35640	ATSERAT3;2 (SERINE ACETYLTRANSFERASE 3;2); acetylt	>0.05	0.034	2.300	1.837	1.099	0.963
1171	At5g42900	similar to unknown protein [Arabidopsis thaliana] (TAII	0.000	>0.05	2.796	2.023	0.907	0.388
1172	At2g40080	ELF4 (EARLY FLOWERING 4)	0.047	>0.05	1.569	2.222	0.657	0.731
1173	At5g43570	serine-type endopeptidase inhibitor	0.026	>0.05	1.350	2.604	1.436	0.610
1174	At2g40340	AP2 domain-containing transcription factor, putative (I	>0.05	0.025	1.662	2.386	1.371	0.800
1175	At3g22231	PCC1 (PATHOGEN AND CIRCADIAN CONTROLLED 1)	>0.05	0.025	2.858	3.490	1.849	0.720
1176	At1g68450	VQ motif-containing protein	0.015	0.009	0.953	2.698	1.456	0.922
1177	At2g02120	LCR70/PDF2.1 (Low-molecular-weight cysteine-rich 70)	0.036	>0.05	0.859	2.223	1.423	0.814
1178	At2g28700	AGL46; DNA binding / transcription factor	0.032	0.026	1.120	2.615	1.201	0.956
1179	At3g04420	ANAC048 (Arabidopsis NAC domain containing protein	0.020	0.040	1.030	2.374	1.146	0.847
1180	At5g43580	serine-type endopeptidase inhibitor	0.035	>0.05	0.829	2.371	1.110	0.581
1181	At1g68500	similar to hypothetical protein [Vitis vinifera] (GB:CAN(	0.021	>0.05	1.253	2.303	0.911	0.705
1182	At5g03545	unknown protein	0.013	>0.05	1.099	2.169	0.979	0.619
1183	At1g52690	late embryogenesis abundant protein, putative / LEA p	0.022	>0.05	0.996	2.578	0.734	1.339
1184	At1g02520	PGP11 (P-GLYCOPROTEIN 11); ATPase, coupled to tran:	0.049	0.041	1.066	2.324	0.984	1.299
1185	At4g31240	electron carrier	0.025	0.005	1.190	2.018	1.047	1.235
1186	At4g11600	ATGPX6 (GLUTATHIONE PEROXIDASE 6); glutathione pe	0.019	0.016	1.324	2.172	0.850	1.100
1187	At5g05960	protease inhibitor/seed storage/lipid transfer protein (	0.032	0.034	0.757	2.150	3.435	0.927
1188	At5g22545	similar to unknown protein [Arabidopsis thaliana] (TAII	0.038	0.042	0.755	2.200	2.854	0.981
1189	At5g16360	NC domain-containing protein	0.017	0.023	0.742	1.811	2.135	0.961
1190	At4g14390	ankyrin repeat family protein	0.045	>0.05	0.716	1.645	2.147	0.971
1191	At1g64160	disease resistance-responsive family protein / dirigent	0.026	>0.05	0.727	2.373	3.975	0.731
1192	At5g01870	lipid transfer protein, putative	0.021	>0.05	0.671	1.800	3.240	0.740
1193	At2g19970	pathogenesis-related protein, putative	0.028	>0.05	0.446	1.699	2.732	0.882
1194	At5g15120	similar to unknown protein [Arabidopsis thaliana] (TAII	0.017	>0.05	0.733	1.039	2.072	0.848
1195	At3g02550	LBD41 (LOB DOMAIN-CONTAINING PROTEIN 41)	0.012	>0.05	0.735	1.004	2.028	0.774
1196	At2g16385	similar to unknown protein [Arabidopsis thaliana] (TAII	0.017	>0.05	0.697	1.309	3.263	0.765
1197	At5g39890	similar to unknown protein [Arabidopsis thaliana] (TAII	0.013	>0.05	0.916	0.944	2.680	0.677
1198	At5g14650	polygalacturonase, putative / pectinase, putative	0.018	>0.05	0.526	1.621	2.305	0.511
1199	At5g11420	similar to unknown protein [Arabidopsis thaliana] (TAII	0.012	0.021	0.774	0.974	2.326	1.330
1200	At5g44420	PDF1.2 (Low-molecular-weight cysteine-rich 77)	0.017	0.012	0.472	0.991	12.577	5.452
1201	At1g75830	LCR67/PDF1.1 (Low-molecular-weight cysteine-rich 67)	0.018	0.010	0.533	0.913	14.635	5.030
1202	At1g67760	ATP binding / protein binding / unfolded protein bindir	0.023	0.027	0.744	1.034	2.714	1.681
1203	At2g26020	PDF1.2b (plant defensin 1.2b)	0.012	0.010	0.564	0.908	11.183	3.060
1204	At5g44430	PDF1.2c (plant defensin 1.2c)	0.018	0.008	0.696	0.912	15.505	4.179
1205	At1g19380	similar to unknown protein [Arabidopsis thaliana] (TAII	>0.05	0.048	0.951	0.959	2.426	1.632
1206	At5g24420	glucosamine/galactosamine-6-phosphate isomerase-re	0.039	>0.05	0.894	0.815	2.159	1.308
1207	At2g15530	zinc finger (C3HC4-type RING finger) family protein	0.024	>0.05	0.839	0.737	2.758	1.337
1208	At2g18010	auxin-responsive family protein	0.010	0.038	0.817	0.745	2.704	1.416
1209	At2g17160	protein kinase-related	0.022	>0.05	0.892	0.812	2.553	1.481
1210	At4g27310	zinc finger (B-box type) family protein	0.020	0.030	0.935	0.843	2.469	1.420
1211	At2g26010	PDF1.3 (plant defensin 1.3)	0.013	0.013	0.735	0.771	8.512	2.350
1212	At2g17530	protein kinase family protein	0.016	0.045	0.843	0.819	2.078	1.473
1213	At2g15340	glycine-rich protein	0.009	>0.05	0.750	0.834	2.953	1.116
1214	At2g18210	similar to unknown protein [Arabidopsis thaliana] (TAII	0.032	>0.05	0.758	0.811	2.907	1.178
1215	At2g15390	FUT4 (fucosyltransferase 4); fucosyltransferase/ transfe	0.001	>0.05	0.792	0.771	3.185	1.227
1216	At2g18140	peroxidase, putative	0.021	>0.05	0.810	0.736	3.374	1.320
1217	At2g15420	myosin heavy chain-related	0.026	>0.05	0.838	0.721	3.384	1.221

1218	At2g18220	Identical to Nucleolar complex protein 2 homolog [Ara	0.021	>0.05	0.827	0.734	3.513	1.162
1219	At2g15430	RBP36A (RNA polymerase II 36 kDa polypeptide A); DN	0.026	>0.05	0.931	0.853	2.140	1.097
1220	At2g16300	F-box family protein	0.011	0.028	0.920	0.815	2.823	1.154
1221	At2g16060	AHB1 (ARABIDOPSIS HEMOGLOBIN 1)	0.017	>0.05	0.916	0.905	2.069	1.067
1222	At2g18150	peroxidase, putative	0.030	>0.05	0.835	0.874	3.497	1.307
1223	At1g29920	CAB2 (Chlorophyll a/b-binding protein 2); chlorophyll b	0.011	>0.05	0.712	0.775	2.204	1.203
1224	At3g26200	CYP71B22 (cytochrome P450, family 71, subfamily B, p	0.034	>0.05	0.717	0.747	2.265	1.403
1225	At4g37770	ACS8 (1-Amino-cyclopropane-1-carboxylate synthase 8	0.020	>0.05	0.507	0.574	5.306	2.458
1226	At1g29660	GDSL-motif lipase/hydrolase family protein	0.011	>0.05	0.965	0.726	2.009	1.069
1227	At5g26150	protein kinase family protein	0.031	>0.05	0.913	0.699	2.242	1.009
1228	At5g04120	phosphoglycerate/bisphosphoglycerate mutase family	0.029	>0.05	0.842	0.622	2.923	1.118
1229	At3g59060	PIL6 (PHYTOCHROME-INTERACTING FACTOR 5); DNA b	0.017	>0.05	0.944	0.757	2.178	1.078
1230	At2g18200	similar to unknown protein [Arabidopsis thaliana] (TAII	0.024	>0.05	0.845	0.740	2.276	1.140
1231	At2g14580	ATPRB1 (Arabidopsis thaliana basic pathogenesis-relat	0.043	>0.05	0.844	0.818	2.001	0.983
1232	At2g16575	similar to unknown protein [Arabidopsis thaliana] (TAII	0.015	>0.05	0.756	0.727	3.024	0.964
1233	At2g15500	similar to PAB2 (POLY(A) BINDING PROTEIN 2), RNA bir	0.013	>0.05	0.708	0.727	3.427	0.970
1234	At2g17525	pentatricopeptide (PPR) repeat-containing protein	0.010	>0.05	0.744	0.741	3.342	0.989
1235	At2g16580	auxin-responsive protein, putative	0.030	>0.05	0.751	0.758	2.608	0.947
1236	At2g15400	RBP36B (RNA polymerase II 36 kDa polypeptide B); DN.	0.013	>0.05	0.689	0.670	3.661	1.016
1237	At2g17150	RWP-RK domain-containing protein	0.020	>0.05	0.768	0.755	2.503	1.034
1238	At2g15520	transposable element gene	0.010	>0.05	0.731	0.726	3.386	1.040
1239	At2g15550	transposable element gene	0.014	>0.05	0.764	0.737	3.096	1.037
1240	At2g15535	LCR10 (Low-molecular-weight cysteine-rich 10)	0.017	>0.05	0.722	0.703	3.807	1.077
1241	At2g17480	MLO8 (MILDEW RESISTANCE LOCUS O 8); calmodulin b	0.030	>0.05	0.732	0.780	2.569	1.083
1242	At2g16450	F-box family protein	0.018	>0.05	0.746	0.693	3.538	0.894
1243	At2g17310	SON1 (SUPPRESSOR OF NIM1-1 1)	0.020	>0.05	0.795	0.737	2.728	0.937
1244	At2g16587	expressed protein	0.013	>0.05	0.754	0.743	3.269	0.899
1245	At2g18080	EDA2 (embryo sac development arrest 2); serine-type p	0.013	>0.05	0.796	0.740	3.226	0.894
1246	At2g15327	unknown protein	0.020	>0.05	0.839	0.851	2.452	0.961
1247	At2g17210	pentatricopeptide (PPR) repeat-containing protein	0.016	>0.05	0.838	0.761	3.257	0.927
1248	At4g09400	transposable element gene	0.015	>0.05	0.856	0.763	3.372	0.971
1249	At3g13140	hydroxyproline-rich glycoprotein family protein	0.010	>0.05	0.928	0.862	2.234	0.893
1250	At5g04310	pectate lyase family protein	0.031	>0.05	0.885	0.866	2.105	0.915
1251	At2g17270	mitochondrial substrate carrier family protein	0.032	>0.05	0.763	0.853	2.212	0.888
1252	At2g18270	unknown protein	0.033	>0.05	0.754	0.724	3.257	0.789
1253	At2g17510	EMB2763 (EMBRYO DEFECTIVE 2763); RNA binding / ri	0.009	>0.05	0.713	0.725	2.459	0.825
1254	At2g15345	enzyme inhibitor/ pectinesterase	0.044	>0.05	0.772	0.732	2.106	1.005
1255	At2g16440	DNA replication licensing factor, putative	0.012	>0.05	0.746	0.689	2.436	0.986
1256	At2g16530	3-oxo-5-alpha-steroid 4-dehydrogenase family protein	0.048	>0.05	0.743	0.771	2.317	0.755
1257	At2g17470	similar to unknown protein [Arabidopsis thaliana] (TAII	0.040	>0.05	0.719	0.692	2.882	0.631
1258	At4g10270	wound-responsive family protein	0.011	>0.05	0.684	0.884	2.022	0.829
1259	At1g78460	SOUL heme-binding family protein	0.031	>0.05	1.143	0.918	2.131	0.966
1260	At5g19110	extracellular dermal glycoprotein-related / EDGP-relate	0.030	>0.05	0.483	0.484	3.354	2.285
1261	At3g12610	DRT100 (DNA-DAMAGE REPAIR/TOLERATION 100); pro	0.007	>0.05	0.634	0.473	2.088	2.096
1262	At4g33220	pectinesterase family protein	0.010	>0.05	0.893	0.520	2.312	1.390
1263	At4g33720	pathogenesis-related protein, putative	0.042	>0.05	0.675	0.486	3.712	1.355
1264	At2g24850	TAT3 (TYROSINE AMINOTRANSFERASE 3); transaminase	0.018	0.036	1.355	0.328	6.414	3.198
1265	At5g02760	protein phosphatase 2C family protein / PP2C family pr	0.008	0.012	1.060	0.670	2.097	1.581
1266	At3g12710	methyladenine glycosylase family protein	0.019	>0.05	0.909	0.644	2.442	1.892

1267	At1g20190	ATEXPA11 (ARABIDOPSIS THALIANA EXPANSIN A11)	0.018	0.037	0.928	0.602	2.610	2.636
1268	At3g49110	ATPCA/ATPRX33/PRX33/PRXCA (PEROXIDASE 33); perc	0.018	0.024	1.148	0.654	1.898	2.115
1269	At5g36270	similar to DHAR2, glutathione dehydrogenase (ascorba	0.020	>0.05	0.404	0.699	1.864	1.216
1270	At3g56220	transcription regulator	0.035	>0.05	0.487	0.743	1.451	1.321
1271	At3g09940	ATMDAR3/MDHAR (MONODEHYDROASCORBATE REDU	0.024	>0.05	0.377	0.530	1.780	1.119
1272	At1g16400	CYP79F2 (cytochrome P450, family 79, subfamily F, pol	0.019	>0.05	0.416	0.862	1.973	1.690
1273	At3g19710	BCAT4 (BRANCHED-CHAIN AMINOTRANSFERASE4); cat	0.021	>0.05	0.278	0.868	1.722	1.972
1274	At3g56390	unknown protein	0.032	>0.05	0.494	1.109	1.792	1.033
1275	At1g62560	flavin-containing monooxygenase family protein / FMC	0.035	>0.05	0.571	0.824	1.259	2.389
1276	At1g65860	flavin-containing monooxygenase family protein / FMC	0.022	>0.05	0.460	0.557	1.323	2.715
1277	At3g57240	BG3 (BETA-1,3-GLUCANASE 3); hydrolase, hydrolyzing (	>0.05	0.046	2.033	1.493	4.340	1.192
1278	At5g06510	CCAAT-binding transcription factor (CBF-B/NF-YA) fami	0.031	0.001	1.279	3.535	4.020	2.439
1279	At4g17030	ATEXLB1 (ARABIDOPSIS THALIANA EXPANSIN-LIKE B1)	>0.05	0.002	1.107	2.527	3.032	1.920
1280	At3g05690	ATHAP2B/HAP2B/UNE8 (HEME ACTIVATOR PROTEIN (Y	>0.05	0.007	1.237	2.553	3.586	2.056
1281	At5g52720	metal ion binding	>0.05	0.006	1.092	3.883	4.309	2.082
