# Supplementary material for: Alkamides Activate Jasmonic Acid Biosynthesis and Signaling Pathways and Confer Resistance to Botrytis cinerea in Arabidopsis thaliana
Source: PLoS One. 2011 Nov 4;6(11):e27251. doi: 10.1371/journal.pone.0027251 (PMC3208606; doi:10.1371/journal.pone.0027251)
Supplement: Table S2 — Functional categories over-represented in N -isobutyl decanamide responsive genes. (PDF) [file pone.0027251.s006.pdf]

Table S2. Functional categories over-represented in *N*-isobutyl decanamide responsive genes. GO categorization was obtained according to the Munich Information Center for Protein Sequences classification (MIPS, http://mips.helmholtz-muenchen.de/proj/funcatDB/) and *Arabidopsis* annotation. Hypergeometric method and a Bonferroni correction were used for the analysis with a cutoff of 0.05. Bold capital fonts indicate significant categories, lowercase black letters subcategorie, and font grey letters correspond to not statitical significative categories (p-value ≤ 0.05).

<table>
<tr><th></th><th>FUNCTIONAL CATEGORY</th><th>abs SET</th><th>rel SET</th><th>genes SET</th><th>abs GENOME</th><th>P-VALUE</th></tr>
<tr><td>01</td><td>METABOLISM</td><td>290</td><td>22.7</td><td>AT1G02520|AT1G02640|AT1G02660|AT1G02850|AT1G02920|AT1G02930|
AT1G05650|AT1G05680|AT1G06120|AT1G06350|AT1G07550|AT1G07560|
AT1G09500|AT1G12010|AT1G13420|AT1G15380|AT1G16400|AT1G16410|
AT1G17020|AT1G17180|AT1G17190|AT1G17420|AT1G17710|AT1G17745|
AT1G21100|AT1G22740|AT1G23730|AT1G23870|AT1G25230|AT1G29660|
AT1G33030|AT1G35190|AT1G35910|AT1G43910|AT1G47840|AT1G48605|
AT1G49860|AT1G51420|AT1G51470|AT1G52400|AT1G52700|AT1G53680|
AT1G54000|AT1G54020|AT1G56600|AT1G56650|AT1G58370|AT1G59700|
AT1G60140|AT1G60470|AT1G61380|AT1G61550|AT1G63180|AT1G64160|
AT1G65060|AT1G65310|AT1G66700|AT1G68290|AT1G69920|AT1G69930|
AT1G70130|AT1G73010|AT1G74010|AT1G74590|AT1G77120|AT1G77530|
AT1G78290|AT1G78380|AT1G80380|AT2G01880|AT2G02010|AT2G02930|
AT2G02990|AT2G03760|AT2G06050|AT2G15390|AT2G16440|AT2G16530|
AT2G17510|AT2G17530|AT2G18360|AT2G18560|AT2G18700|AT2G18800|
AT2G19060|AT2G19190|AT2G19800|AT2G21880|AT2G22190|AT2G22330|
AT2G22590|AT2G23910|AT2G24850|AT2G26560|AT2G28970|AT2G28990|
AT2G29350|AT2G29440|AT2G29460|AT2G29470|AT2G31880|AT2G32290|
AT2G34180|AT2G36750|AT2G36780|AT2G36790|AT2G36800|AT2G36970|
AT2G38600|AT2G39030|AT2G39350|AT2G39800|AT2G41970|AT2G43570|
AT2G43580|AT2G43590|AT2G43880|AT2G44460|AT2G47180|AT3G01500|
AT3G02830|AT3G03190|AT3G03530|AT3G08860|AT3G09940|AT3G12500|
AT3G13610|AT3G13650|AT3G13750|AT3G14210|AT3G14440|AT3G15354|
AT3G16360|AT3G17790|AT3G18000|AT3G18080|AT3G19620|AT3G19710|
AT3G21340|AT3G21870|AT3G23510|AT3G23530|AT3G23730|AT3G25250|
AT3G25760|AT3G25770|AT3G25820|AT3G25830|AT3G26610|AT3G26830|
[illegible]</td><td>4932</td><td>3.23E-07</td></tr>
<tr><td>01.01</td><td>amino acid metabolism</td><td>19</td><td>1.48</td><td>AT1G17745|AT2G02010|AT2G22330|AT2G24850|AT2G39800|AT3G08860|
AT3G30775|AT3G54640|AT4G08870|AT4G23600|AT4G34710|AT4G35640|
AT4G37770|AT4G37990|AT5G18170|AT5G19530|AT5G35630|AT5G37600|
AT5G66120</td><td>342</td><td>2.01E-01</td></tr>
<tr><td>01.01.03</td><td>assimilation of ammonia, metabolism of the glutamate group</td><td>6</td><td>0.47</td><td>AT2G02010|AT2G39800|AT3G30775|AT4G34710|AT5G35630|AT5G37600</td><td>57</td><td>4.20E-02</td></tr>
<tr><td>01.01.03.01</td><td>metabolism of glutamine</td><td>3</td><td>0.23</td><td>AT4G34710|AT5G35630|AT5G37600</td><td>11</td><td>1.14E-02</td></tr>
<tr><td>01.01.05</td><td>metabolism of urea cycle, creatine and polyamines</td><td>3</td><td>0.23</td><td>AT4G08870|AT4G34710|AT5G19530</td><td>23</td><td>8.21E-02</td></tr>
<tr><td>01.01.05.01</td><td>metabolism of polyamines</td><td>3</td><td>0.23</td><td>AT4G08870|AT4G34710|AT5G19530</td><td>14</td><td>2.27E-02</td></tr>
<tr><td>01.02</td><td>nitrogen, sulfur and selenium metabolism</td><td>15</td><td>1.17</td><td>AT1G13420|AT2G03760|AT2G24850|AT3G44300|AT3G44310|AT3G45070|
AT3G60180|AT4G16310|AT4G23600|AT4G26280|AT4G29740|AT4G35640|
AT5G09290|AT5G35630|AT5G37600</td><td>162</td><td>6.45E-03</td></tr>
</table>

01.02.02	nitrogen metabolism	8	0.62	AT2G24850|AT3G44300|AT3G44310|AT4G16310|AT4G23600|AT4G29740|AT5G35630|AT5G37600	86	3.93E-02
01.02.02.06	assimilation of ammonia	1	0.07	AT5G35630	1	4.49E-02
01.02.03	sulfur metabolism	7	0.54	AT1G13420|AT2G03760|AT3G45070|AT3G60180|AT4G26280|AT4G35640|AT5G09290	72	4.25E-02
01.02.03.04	conjunction of sulfate	4	0.31	AT1G13420|AT2G03760|AT3G45070|AT4G26280	22	1.55E-02
01.05	C-compound and carbohydrate metabolism	129	10.1	AT1G02640|AT1G02850|AT1G05650|AT1G05680|AT1G09500|AT1G15380|AT1G21100|AT1G23730|AT1G23870|AT1G29660|AT1G33030|AT1G35910|AT1G51420|AT1G51470|AT1G52400|AT1G52700|AT1G54000|AT1G54020|AT1G56600|AT1G58370|AT1G60140|AT1G60470|AT1G63180|AT1G65310|AT1G66700|AT1G77120|AT1G77530|AT2G01880|AT2G02010|AT2G15390|AT2G18360|AT2G18560|AT2G18700|AT2G18800|AT2G19800|AT2G22190|AT2G22590|AT2G29350|AT2G32290|AT2G36750|AT2G36780|AT2G36790|AT2G36800|AT2G36970|AT2G38600|AT2G39030|AT2G43570|AT2G43580|AT2G43590|AT2G43880|AT2G44460|AT2G47180|AT3G01500|AT3G12500|AT3G13750|AT3G14210|AT3G17790|AT3G18000|AT3G18080|AT3G19620|AT3G23510|AT3G23530|AT3G23730|AT3G26610|AT3G44860|AT3G44870|AT3G47540|AT3G48580|AT3G50760|AT3G52340|AT3G52780|AT3G53150|AT3G53160|AT3G55630|AT3G56000|AT3G57240|AT3G57260|AT3G57520|AT3G59480|AT3G60140|AT3G61990|AT4G02280|AT4G08160|AT4G09020|AT4G12830|AT4G15210|AT4G15290|AT4G15480|AT4G15490|AT4G16310|AT4G18340|AT4G19810|AT4G20460|AT4G23990|AT4G24380|AT4G25820|AT4G25900|AT4G26260|AT4G26270|AT4G26530|AT4G29740|AT4G30140|AT4G30290|AT4G37770|AT4G37990|AT5G05900|AT5G14470|AT5G14650|AT5G14740|AT5G19040|AT5G20250|AT5G24410|AT5G24420|AT5G24770|AT5G28510|AT5G38420|AT5G39320|AT5G41080|AT5G42800|AT5G44020|AT5G49360|AT5G53990|AT5G54060|AT5G55480|AT5G56630|AT5G56870|AT5G59520|AT5G65730|AT5G66120	1646	4.18E-10
01.05.02	sugar, glucoside, polyol and carboxylate metabolism	67	5.25	AT1G02640|AT1G02850|AT1G05680|AT1G23870|AT1G35910|AT1G51420|AT1G51470|AT1G52400|AT1G56600|AT1G58370|AT1G60140|AT1G60470|AT1G63180|AT2G15390|AT2G18560|AT2G18700|AT2G19800|AT2G22190|AT2G22590|AT2G36750|AT2G36780|AT2G36790|AT2G36800|AT2G36970|AT2G44460|AT2G47180|AT3G13750|AT3G18080|AT3G19620|AT3G50760|AT3G52340|AT3G53150|AT3G53160|AT3G56000|AT3G57240|AT3G57260|AT3G57520|AT3G59480|AT3G60140|AT4G02280|AT4G08160|AT4G15290|AT4G15480|AT4G15490|AT4G18340|AT4G19810|AT4G20460|AT4G23990|AT4G25900|AT4G26260|AT4G26270|AT4G26530|AT4G37990|AT5G05900|AT5G14470|AT5G20250|AT5G24410|AT5G24420|AT5G28510|AT5G38420|AT5G39320|AT5G49360|AT5G53990|AT5G54060|AT5G56630|AT5G56870|AT5G59520	853	7.18E-06
01.05.02.01	nucleotide-sugar metabolism	16	1.25	AT1G05680|AT1G63180|AT2G18560|AT2G36750|AT2G36780|AT2G36790|AT2G36800|AT2G36970|AT3G53150|AT3G53160|AT4G02280|AT4G15480|AT4G15490|AT4G20460|AT5G05900|AT5G39320	185	9.47E-03
01.05.02.04	sugar, glucoside, polyol and carboxylate anabolism	9	0.7	AT1G23870|AT1G35910|AT1G51420|AT1G60140|AT2G18700|AT2G22190|AT3G52340|AT3G59480|AT4G02280	60	1.35E-03

01.05.03	polysaccharide metabolism	17	1.33	AT1G05650|AT2G32290|AT2G43570|AT2G43580|AT2G43590|AT2G43880|AT3G12500|AT3G26610|AT3G47540|AT3G56000|AT3G57240|AT3G57260|AT4G09020|AT4G15210|AT4G15290|AT4G23990|AT5G14650	238	4.08E-02
01.05.03.03	chitin metabolism	5	0.39	AT2G43570|AT2G43580|AT2G43590|AT3G12500|AT3G47540	14	2.58E-04
01.05.03.03.07	chitin catabolism	5	0.39	AT2G43570|AT2G43580|AT2G43590|AT3G12500|AT3G47540	14	2.58E-04
01.05.03.04.07	starch catabolism	3	0.23	AT2G32290|AT4G09020|AT4G15210	17	3.84E-02
01.06	lipid, fatty acid and isoprenoid metabolism	36	2.82	AT1G02660|AT1G06120|AT1G06350|AT1G17420|AT1G29660|AT1G54000|AT1G54020|AT1G74010|AT1G80380|AT2G16530|AT2G19060|AT2G26560|AT3G03530|AT3G14440|AT3G18000|AT3G23510|AT3G23530|AT3G25820|AT3G25830|AT3G45140|AT3G57020|AT4G15340|AT4G15440|AT4G25700|AT4G29800|AT4G30140|AT4G37760|AT5G09290|AT5G22020|AT5G41080|AT5G47980|AT5G48010|AT5G48880|AT5G55480|AT5G57240|AT5G58770	827	1.00E+00
01.06.06	isoprenoid metabolism	10	0.78	AT1G74010|AT3G14440|AT3G25820|AT3G25830|AT3G57020|AT4G15340|AT4G25700|AT5G22020|AT5G48010|AT5G58770	177	2.74E-01
01.06.06.03	monoterpenes metabolism	2	0.15	AT3G25820|AT3G25830	4	1.14E-02
01.20	secondary metabolism	67	5.25	AT1G02920|AT1G02930|AT1G09500|AT1G12010|AT1G16400|AT1G16410|AT1G17020|AT1G17180|AT1G17190|AT1G17420|AT1G33030|AT1G35190|AT1G49860|AT1G53680|AT1G56650|AT1G59700|AT1G64160|AT1G65060|AT1G69920|AT1G69930|AT1G74010|AT1G74590|AT1G77530|AT1G78380|AT2G02930|AT2G02990|AT2G06050|AT2G22330|AT2G23910|AT2G29440|AT2G29460|AT2G29470|AT2G36800|AT3G03190|AT3G13610|AT3G13650|AT3G14210|AT3G19710|AT3G25760|AT3G25770|AT3G26830|AT3G44300|AT3G44310|AT3G45140|AT3G51240|AT3G55120|AT3G57020|AT3G62760|AT4G02520|AT4G08870|AT4G10500|AT4G11210|AT4G22880|AT4G29740|AT4G34710|AT4G36220|AT4G37770|AT5G05270|AT5G07990|AT5G08640|AT5G13930|AT5G17220|AT5G22020|AT5G42800|AT5G43450|AT5G63600|AT5G66120	426	3.01E-19
01.20.05	metabolism of acetic acid derivatives	5	0.39	AT1G17420|AT2G06050|AT3G25760|AT3G25770|AT3G45140	23	3.10E-03
01.20.17	metabolism of secondary products derived from primary amino acids	7	0.54	AT1G16400|AT1G16410|AT1G35190|AT2G22330|AT3G19710|AT3G57020|AT5G22020	46	4.18E-03
01.20.17.07	metabolism of glycosinolates and derivatives	4	0.31	AT1G16400|AT1G16410|AT2G22330|AT3G19710	17	6.03E-03
01.20.17.09	metabolism of alkaloids	3	0.23	AT1G35190|AT3G57020|AT5G22020	17	3.84E-02
01.20.31	metabolism of secondary products derived from L-lysine, L-arginine and L-histidine	2	0.15	AT4G08870|AT4G34710	6	2.68E-02

01.20.33	metabolism of secondary products derived from L-tryptophan	7	0.54	AT1G74010|AT2G22330|AT3G26830|AT3G44300|AT3G44310|AT3G57020|AT5G22020	38	1.35E-03
01.20.35	metabolism of secondary products derived from L-phenylalanine and L-tyrosine	21	1.64	AT1G09500|AT1G17020|AT1G33030|AT1G56650|AT1G64160|AT1G65060|AT1G77530|AT2G02990|AT2G23910|AT3G13650|AT3G51240|AT3G55120|AT4G11210|AT4G22880|AT4G36220|AT5G05270|AT5G07990|AT5G08640|AT5G13930|AT5G42800|AT5G63600	129	3.04E-07
01.20.35.01	metabolism of phenylpropanoids	21	1.64	AT1G09500|AT1G17020|AT1G33030|AT1G56650|AT1G64160|AT1G65060|AT1G77530|AT2G02990|AT2G23910|AT3G13650|AT3G51240|AT3G55120|AT4G11210|AT4G22880|AT4G36220|AT5G05270|AT5G07990|AT5G08640|AT5G13930|AT5G42800|AT5G63600	117	5.41E-08
01.20.35.01.03	metabolism of lignins	8	0.62	AT1G09500|AT1G33030|AT1G64160|AT1G77530|AT2G23910|AT3G13650|AT4G11210|AT4G36220	46	9.22E-04
01.20.35.01.05	metabolism of stilbenes, flavonoids	12	0.94	AT1G17020|AT1G56650|AT2G02990|AT3G51240|AT3G55120|AT4G22880|AT5G05270|AT5G07990|AT5G08640|AT5G13930|AT5G42800|AT5G63600	46	6.02E-07
01.20.38	metabolism of toxins/drugs	19	1.48	AT1G02920|AT1G02930|AT1G17180|AT1G17190|AT1G49860|AT1G53680|AT1G59700|AT1G69920|AT1G69930|AT1G74590|AT1G78380|AT2G02930|AT2G29440|AT2G29460|AT2G29470|AT3G03190|AT3G62760|AT4G02520|AT5G17220	48	7.25E-14
**02**	**ENERGY**	26	2.03	AT1G14150|AT1G19150|AT1G23870|AT1G29920|AT1G35910|AT1G51420|AT1G60140|AT1G77120|AT1G80380|AT2G22190|AT3G21720|AT3G22370|AT3G52340|AT3G59480|AT3G60180|AT4G09020|AT4G15210|AT4G26270|AT4G26530|AT4G28660|AT5G09470|AT5G24410|AT5G24420|AT5G48880|AT5G56630|AT5G65690	453	1.21E-01
02.01	glycolysis and gluconeogenesis	7	0.54	AT1G51420|AT1G80380|AT3G52340|AT4G26270|AT4G26530|AT5G56630|AT5G65690	73	4.53E-02
02.19	metabolism of energy reserves (e.g. glycogen, trehalose)	6	0.47	AT1G23870|AT1G35910|AT1G60140|AT2G22190|AT4G09020|AT4G15210	53	3.09E-02
**04**	**STORAGE PROTEIN**	16	1.25	AT1G72610|AT2G26560|AT3G05950|AT4G14630|AT5G20630|AT5G25610|AT5G38910|AT5G38930|AT5G38940|AT5G39110|AT5G39120|AT5G39130|AT5G39150|AT5G39160|AT5G39180|AT5G39190	65	2.02E-08
04.01	storage facilitating proteins	16	1.25	AT1G72610|AT2G26560|AT3G05950|AT4G14630|AT5G20630|AT5G25610|AT5G38910|AT5G38930|AT5G38940|AT5G39110|AT5G39120|AT5G39130|AT5G39150|AT5G39160|AT5G39180|AT5G39190	65	2.02E-08

**11**	**TRANSCRIPTION**	103	8.07	AT1G04240|AT1G13300|AT1G18330|AT1G19050|AT1G19210|AT1G19510|AT1G21910|AT1G26945|AT1G31050|AT1G43160|AT1G48000|AT1G52890|AT1G53170|AT1G56650|AT1G59940|AT1G60920|AT1G62975|AT1G66230|AT1G66380|AT1G66600|AT1G67710|AT1G72360|AT1G72830|AT1G74080|AT1G80840|AT2G15400|AT2G15430|AT2G17150|AT2G22630|AT2G28700|AT2G36270|AT2G37430|AT2G40340|AT2G40670|AT2G40750|AT2G43220|AT2G43500|AT2G44840|AT2G46400|AT2G46510|AT2G46680|AT2G47190|AT2G47520|AT3G01970|AT3G04070|AT3G04420|AT3G05690|AT3G11580|AT3G13760|AT3G15210|AT3G23240|AT3G23250|AT3G24310|AT3G24500|AT3G25790|AT3G28730|AT3G46080|AT3G48100|AT3G48360|AT3G48920|AT3G49760|AT3G51910|AT3G56980|AT3G59060|AT4G13130|AT4G13620|AT4G17460|AT4G23810|AT4G25470|AT4G25480|AT4G25490|AT4G27310|AT4G27410|AT4G29190|AT4G30180|AT4G34410|AT4G39070|AT5G02350|AT5G04150|AT5G06510|AT5G08790|AT5G10140|AT5G14750|AT5G15160|AT5G15830|AT5G17300|AT5G21960|AT5G22250|AT5G25810|AT5G28300|AT5G28770|AT5G37260|AT5G43650|AT5G47220|AT5G47230|AT5G50915|AT5G51790|AT5G51990|AT5G58010|AT5G62920|AT5G63160|AT5G64810|AT5G66700	2664	1.00E+00
11.02	RNA synthesis	102	7.99	AT1G04240|AT1G13300|AT1G18330|AT1G19050|AT1G19210|AT1G19510|AT1G21910|AT1G26945|AT1G31050|AT1G43160|AT1G48000|AT1G52890|AT1G53170|AT1G56650|AT1G59940|AT1G60920|AT1G62975|AT1G66230|AT1G66380|AT1G66600|AT1G67710|AT1G72360|AT1G72830|AT1G74080|AT1G80840|AT2G15400|AT2G15430|AT2G17150|AT2G22630|AT2G28700|AT2G36270|AT2G37430|AT2G40340|AT2G40670|AT2G40750|AT2G43220|AT2G43500|AT2G44840|AT2G46400|AT2G46510|AT2G46680|AT2G47190|AT2G47520|AT3G01970|AT3G04070|AT3G04420|AT3G05690|AT3G11580|AT3G13760|AT3G15210|AT3G23240|AT3G23250|AT3G24310|AT3G24500|AT3G25790|AT3G28730|AT3G46080|AT3G48100|AT3G48360|AT3G48920|AT3G49760|AT3G51910|AT3G56980|AT3G59060|AT4G13130|AT4G13620|AT4G17460|AT4G23810|AT4G25470|AT4G25480|AT4G25490|AT4G27310|AT4G27410|AT4G29190|AT4G30180|AT4G34410|AT4G39070|AT5G02350|AT5G04150|AT5G06510|AT5G08790|AT5G10140|AT5G14750|AT5G15160|AT5G15830|AT5G17300|AT5G21960|AT5G25810|AT5G28300|AT5G28770|AT5G37260|AT5G43650|AT5G47220|AT5G47230|AT5G50915|AT5G51790|AT5G51990|AT5G58010|AT5G62920|AT5G63160|AT5G64810|AT5G66700	2298	1.00E+00

11.02.03	mRNA synthesis	100	7.83	AT1G04240|AT1G13300|AT1G18330|AT1G19050|AT1G19210|AT1G19510|AT1G21910|AT1G26945|AT1G31050|AT1G43160|AT1G48000|AT1G52890|AT1G53170|AT1G56650|AT1G59940|AT1G60920|AT1G62975|AT1G66230|AT1G66380|AT1G66600|AT1G67710|AT1G72360|AT1G72830|AT1G74080|AT1G80840|AT2G17150|AT2G22630|AT2G28700|AT2G36270|AT2G37430|AT2G40340|AT2G40670|AT2G40750|AT2G43220|AT2G43500|AT2G44840|AT2G46400|AT2G46510|AT2G46680|AT2G47190|AT2G47520|AT3G01970|AT3G04070|AT3G04420|AT3G05690|AT3G11580|AT3G13760|AT3G15210|AT3G23240|AT3G23250|AT3G24310|AT3G24500|AT3G25790|AT3G28730|AT3G46080|AT3G48100|AT3G48360|AT3G48920|AT3G49760|AT3G51910|AT3G56980|AT3G59060|AT4G13130|AT4G13620|AT4G17460|AT4G23810|AT4G25470|AT4G25480|AT4G25490|AT4G27310|AT4G27410|AT4G29190|AT4G30180|AT4G34410|AT4G39070|AT5G02350|AT5G04150|AT5G06510|AT5G08790|AT5G10140|AT5G14750|AT5G15160|AT5G15830|AT5G17300|AT5G21960|AT5G25810|AT5G28300|AT5G28770|AT5G37260|AT5G43650|AT5G47220|AT5G47230|AT5G50915|AT5G51790|AT5G51990|AT5G58010|AT5G62920|AT5G63160|AT5G64810|AT5G66700	2149	3.68E-01
11.02.03.04	transcriptional control	100	7.83	AT1G04240|AT1G13300|AT1G18330|AT1G19050|AT1G19210|AT1G19510|AT1G21910|AT1G26945|AT1G31050|AT1G43160|AT1G48000|AT1G52890|AT1G53170|AT1G56650|AT1G59940|AT1G60920|AT1G62975|AT1G66230|AT1G66380|AT1G66600|AT1G67710|AT1G72360|AT1G72830|AT1G74080|AT1G80840|AT2G17150|AT2G22630|AT2G28700|AT2G36270|AT2G37430|AT2G40340|AT2G40670|AT2G40750|AT2G43220|AT2G43500|AT2G44840|AT2G46400|AT2G46510|AT2G46680|AT2G47190|AT2G47520|AT3G01970|AT3G04070|AT3G04420|AT3G05690|AT3G11580|AT3G13760|AT3G15210|AT3G23240|AT3G23250|AT3G24310|AT3G24500|AT3G25790|AT3G28730|AT3G46080|AT3G48100|AT3G48360|AT3G48920|AT3G49760|AT3G51910|AT3G56980|AT3G59060|AT4G13130|AT4G13620|AT4G17460|AT4G23810|AT4G25470|AT4G25480|AT4G25490|AT4G27310|AT4G27410|AT4G29190|AT4G30180|AT4G34410|AT4G39070|AT5G02350|AT5G04150|AT5G06510|AT5G08790|AT5G10140|AT5G14750|AT5G15160|AT5G15830|AT5G17300|AT5G21960|AT5G25810|AT5G28300|AT5G28770|AT5G37260|AT5G43650|AT5G47220|AT5G47230|AT5G50915|AT5G51790|AT5G51990|AT5G58010|AT5G62920|AT5G63160|AT5G64810|AT5G66700	2117	3.11E-01
11.02.03.04.01	transcription activation	16	1.25	AT1G72830|AT2G36270|AT2G46680|AT2G47190|AT3G05690|AT3G23240|AT3G24500|AT4G25470|AT4G25480|AT4G25490|AT4G27410|AT5G06510|AT5G08790|AT5G47220|AT5G47230|AT5G51990	114	5.08E-05
**12**	**PROTEIN SYNTHESIS**	2	0.15	AT2G27535|AT4G34620	1332	1.00E+00
12.04	translation	2	0.15	AT2G27535|AT4G34620	1218	1.00E+00

<table>
<tr><td>16</td><td>PROTEIN WITH BINDING FUNCTION OR COFACTOR REQUIREMENT (structural or catalytic)</td><td>302</td><td>23.6</td><td>AT1G02520|AT1G03440|AT1G07610|AT1G11740|AT1G12740|AT1G13195|
AT1G13300|AT1G14150|AT1G14200|AT1G16400|AT1G17420|AT1G17745|
AT1G18330|AT1G19210|AT1G19510|AT1G21312|AT1G21910|AT1G22740|
AT1G23730|AT1G24530|AT1G31710|AT1G33340|AT1G43160|AT1G43910|
AT1G47840|AT1G48000|AT1G48605|AT1G50520|AT1G50560|AT1G51090|
AT1G52040|AT1G52250|AT1G53170|AT1G55780|AT1G56650|AT1G60190|
AT1G60920|AT1G60970|AT1G61380|AT1G61550|AT1G62975|AT1G63180|
AT1G64950|AT1G66230|AT1G66380|AT1G66400|AT1G68010|AT1G68110|
AT1G68290|AT1G72200|AT1G72260|AT1G72360|AT1G72610|AT1G72830|
AT1G73165|AT1G73800|AT1G74080|AT1G74690|AT1G74760|AT1G75290|
AT1G75580|AT1G75780|AT1G76410|AT1G76640|AT1G78450|AT1G78460|
AT1G78490|AT2G02010|AT2G02850|AT2G14160|AT2G15050|AT2G15400|
AT2G15430|AT2G15530|AT2G16060|AT2G16440|AT2G17270|AT2G17310|
AT2G17480|AT2G17510|AT2G18600|AT2G21880|AT2G22330|AT2G22500|
AT2G24600|AT2G25160|AT2G25430|AT2G28190|AT2G28700|AT2G28970|
AT2G29130|AT2G30210|AT2G30520|AT2G30750|AT2G30770|AT2G31081|
AT2G31082|AT2G31085|AT2G31200|AT2G31880|AT2G32300|AT2G33380|
AT2G34000|AT2G36270|AT2G37430|AT2G37870|AT2G38080|AT2G38230|
AT2G38530|AT2G38540|AT2G39350|AT2G39570|AT2G40340|AT2G41090|
AT2G43220|AT2G44580|AT2G44840|AT2G45040|AT2G46510|AT2G47190|
AT2G47520|AT3G01440|AT3G01500|AT3G02830|AT3G02850|AT3G04720|
AT3G05690|AT3G05950|AT3G09390|AT3G11110|AT3G11840|AT3G12145|
AT3G12610|AT3G13760|AT3G14620|AT3G15210|AT3G15354|AT3G20140|
AT3G20940|AT3G21180|AT3G22570|AT3G23240|AT3G23250|AT3G24310|
AT3G24500|AT3G25790|AT3G26180|AT3G26200|AT3G26830|AT3G27070|
[illegible]</td><td>7027</td><td>1.00E+00</td></tr>
<tr><td>16.01</td><td>protein binding</td><td>87</td><td>6.81</td><td>AT1G03440|AT1G11740|AT1G13195|AT1G14200|AT1G24530|AT1G52040|
AT1G52250|AT1G60190|AT1G60970|AT1G63180|AT1G72200|AT1G72260|
AT1G72830|AT1G73165|AT1G73800|AT1G74690|AT1G74760|AT1G75580|
AT1G75780|AT1G76410|AT2G02010|AT2G15530|AT2G17310|AT2G17480|
AT2G18600|AT2G24600|AT2G30520|AT2G31081|AT2G31082|AT2G31085|
AT2G31200|AT2G34000|AT2G38230|AT2G38540|AT2G43220|AT2G44580|
AT2G47190|AT3G05690|AT3G11110|AT3G11840|AT3G12145|AT3G12610|
AT3G13760|AT3G15210|AT3G15354|AT3G21180|AT3G24500|AT3G27070|
AT3G48360|AT3G55150|AT3G56710|AT3G60020|AT3G62570|AT3G63380|
AT4G00680|AT4G04620|AT4G10720|AT4G13130|AT4G14365|AT4G14400|
AT4G18510|AT4G23060|AT4G24015|AT4G26270|AT4G34210|AT4G34470|
AT5G02350|AT5G03240|AT5G06510|AT5G06860|AT5G06870|AT5G09800|
AT5G10140|AT5G12940|AT5G14750|AT5G21430|AT5G26920|AT5G27420|
AT5G36910|AT5G37640|AT5G42040|AT5G52640|AT5G53500|AT5G54490|
AT5G54610|AT5G56630|AT5G63160</td><td>2619</td><td>1.00E+00</td></tr>
<tr><td>16.01.01</td><td>receptor binding</td><td>7</td><td>0.54</td><td>AT1G72260|AT1G73165|AT2G31081|AT2G31082|AT2G31085|AT4G18510|
AT5G36910</td><td>31</td><td>3.69E-04</td></tr>
<tr><td>16.09</td><td>lipid binding</td><td>19</td><td>1.48</td><td>AT2G15050|AT2G37870|AT2G38530|AT3G22570|AT4G12490|AT4G12500|
AT4G12510|AT4G12520|AT4G12550|AT4G20370|AT4G22460|AT4G22470|
AT4G33550|AT5G01300|AT5G01870|AT5G05960|AT5G46890|AT5G59310|
AT5G59320</td><td>141</td><td>1.88E-05</td></tr>
</table>

16.17	metal binding	74	5.79	AT1G07610|AT1G13195|AT1G14150|AT1G14200|AT1G17420|AT1G23730|AT1G31710|AT1G51090|AT1G55780|AT1G66400|AT1G72200|AT1G72610|AT1G74760|AT1G76410|AT1G76640|AT2G02850|AT2G15530|AT2G28190|AT2G29130|AT2G30210|AT2G32300|AT2G33380|AT2G34000|AT2G37430|AT2G38080|AT2G41090|AT2G43220|AT2G44580|AT2G45040|AT3G01440|AT3G01500|AT3G05950|AT3G09390|AT3G11110|AT3G13760|AT3G28210|AT3G46080|AT3G47480|AT3G50770|AT4G01380|AT4G12290|AT4G13130|AT4G14365|AT4G14630|AT4G15440|AT4G24015|AT4G25380|AT4G27310|AT4G37970|AT4G39070|AT5G02350|AT5G14740|AT5G16980|AT5G17000|AT5G20630|AT5G22920|AT5G26690|AT5G27420|AT5G38910|AT5G38930|AT5G38940|AT5G39110|AT5G39120|AT5G39130|AT5G39150|AT5G39160|AT5G39180|AT5G39190|AT5G39670|AT5G42380|AT5G52720|AT5G52750|AT5G52760|AT5G54490	1296	2.05E-02
16.25	oxygen binding	29	2.27	AT1G12740|AT1G16400|AT1G50520|AT1G50560|AT1G64950|AT1G78490|AT2G16060|AT2G22330|AT2G25160|AT2G30750|AT2G30770|AT3G14620|AT3G20140|AT3G20940|AT3G26180|AT3G26200|AT3G26830|AT3G28740|AT4G15300|AT4G22710|AT4G37370|AT4G37410|AT5G07990|AT5G08250|AT5G25130|AT5G36220|AT5G38450|AT5G42580|AT5G57220	235	8.81E-07
**20**	**CELLULAR TRANSPORT, TRANSPORT FACILITIES AND TRANSPORT ROUTES**	161	12.6	AT1G01580|AT1G01620|AT1G02520|AT1G06830|AT1G08430|AT1G12200|AT1G12740|AT1G14185|AT1G16370|AT1G16390|AT1G22530|AT1G22740|AT1G26380|AT1G26390|AT1G26400|AT1G26410|AT1G26420|AT1G28480|AT1G30840|AT1G32350|AT1G42550|AT1G48605|AT1G50520|AT1G50560|AT1G51090|AT1G53030|AT1G55780|AT1G59730|AT1G60970|AT1G61800|AT1G62560|AT1G64780|AT1G64950|AT1G65860|AT1G66130|AT1G67640|AT1G69880|AT1G71140|AT1G73220|AT1G78000|AT1G78490|AT2G02850|AT2G04050|AT2G05620|AT2G15050|AT2G17270|AT2G18480|AT2G21880|AT2G22500|AT2G24710|AT2G25160|AT2G29120|AT2G30540|AT2G30750|AT2G30770|AT2G32300|AT2G34810|AT2G36830|AT2G37170|AT2G37180|AT2G37870|AT2G38530|AT2G38540|AT2G46650|AT3G01760|AT3G02850|AT3G05400|AT3G09340|AT3G09580|AT3G09940|AT3G14620|AT3G16240|AT3G20140|AT3G20940|AT3G21180|AT3G22370|AT3G22570|AT3G24300|AT3G26180|AT3G26200|AT3G26520|AT3G27070|AT3G28740|AT3G45690|AT3G45700|AT3G45710|AT3G45720|AT3G48850|AT3G51895|AT3G55130|AT3G55150|AT3G59020|AT3G61430|AT3G62950|AT3G63380|AT4G01380|AT4G04850|AT4G08620|AT4G12490|AT4G12500|AT4G12510|AT4G12520|AT4G12550|AT4G13420|AT4G14450|AT4G15300|AT4G15660|AT4G15670|AT4G15680|AT4G15690|AT4G15700|AT4G16310|AT4G17340|AT4G19030|AT4G19690|AT4G22460|AT4G22470|AT4G22710|AT4G23700|AT4G24120|AT4G33040|AT4G33550|AT4G34580|AT4G35100|AT4G35180|AT4G37370|AT4G37410|AT5G01180|AT5G01870|AT5G02270|AT5G03555|AT5G05960|AT5G08250|AT5G09470|AT5G14070|AT5G16530|AT5G17860|AT5G18600|AT5G24030|AT5G25130|AT5G26690|AT5G36220|AT5G38450|AT5G39320|AT5G40370|AT5G42580|AT5G43350|AT5G43370|AT5G44110|AT5G46890|[illegible]	2419	2.46E-07

20.01	transported compounds (substrates)	134	10.5	AT1G01580|AT1G06830|AT1G08430|AT1G12200|AT1G12740|AT1G14185|AT1G16370|AT1G16390|AT1G22740|AT1G26380|AT1G26390|AT1G26400|AT1G26410|AT1G26420|AT1G28480|AT1G30840|AT1G32350|AT1G48605|AT1G50520|AT1G50560|AT1G51090|AT1G53030|AT1G55780|AT1G59730|AT1G60970|AT1G61800|AT1G62560|AT1G64780|AT1G64950|AT1G65860|AT1G66130|AT1G67640|AT1G69880|AT1G71140|AT1G73220|AT1G78000|AT1G78490|AT2G02850|AT2G04050|AT2G05620|AT2G15050|AT2G18480|AT2G21880|AT2G25160|AT2G30540|AT2G30750|AT2G30770|AT2G32300|AT2G34810|AT2G37870|AT2G38530|AT2G38540|AT2G46650|AT3G01760|AT3G02850|AT3G05400|AT3G09340|AT3G09580|AT3G09940|AT3G14620|AT3G16240|AT3G20140|AT3G20940|AT3G21180|AT3G22370|AT3G22570|AT3G24300|AT3G26180|AT3G26200|AT3G27070|AT3G28740|AT3G45690|AT3G45700|AT3G45710|AT3G45720|AT3G51895|AT3G59020|AT3G62950|AT3G63380|AT4G01380|AT4G04850|AT4G08620|AT4G12490|AT4G12500|AT4G12510|AT4G12520|AT4G12550|AT4G13420|AT4G15300|AT4G15660|AT4G15670|AT4G15680|AT4G15690|AT4G15700|AT4G16310|AT4G19690|AT4G22460|AT4G22470|AT4G22710|AT4G23700|AT4G24120|AT4G33040|AT4G33550|AT4G35180|AT4G37370|AT4G37410|AT5G01180|AT5G01870|AT5G03555|AT5G05960|AT5G08250|AT5G14070|AT5G16530|AT5G17860|AT5G18600|AT5G25130|AT5G26690|AT5G36220|AT5G38450|AT5G39320|AT5G40370|AT5G42580|AT5G43350|AT5G43370|AT5G46890|AT5G47450|AT5G49630|AT5G52720|AT5G52750|AT5G52760|AT5G57220|AT5G59310|AT5G59520|AT5G61440	1892	9.52E-08
20.01.01	ion transport	26	2.03	AT1G01580|AT1G51090|AT1G53030|AT1G55780|AT1G64780|AT1G78000|AT3G02850|AT3G16240|AT3G21180|AT3G24300|AT3G51895|AT3G63380|AT4G04850|AT4G08620|AT4G13420|AT4G19690|AT4G23700|AT5G17860|AT5G26690|AT5G43350|AT5G43370|AT5G47450|AT5G52720|AT5G52750|AT5G52760|AT5G59520	458	1.32E-01
20.01.01.07	anion transport	5	0.39	AT1G78000|AT3G51895|AT4G08620|AT5G43350|AT5G43370	102	4.86E-01
20.01.01.07.05	sulfate/sulfite transport	3	0.23	AT1G78000|AT3G51895|AT4G08620	14	2.27E-02
20.01.13	lipid/fatty acid transport	17	1.33	AT2G15050|AT2G37870|AT2G38530|AT2G38540|AT3G22570|AT4G12490|AT4G12500|AT4G12510|AT4G12520|AT4G12550|AT4G22460|AT4G22470|AT4G33550|AT5G01870|AT5G05960|AT5G46890|AT5G59310	123	3.66E-05
20.01.15	electron transport	65	5.09	AT1G01580|AT1G06830|AT1G12200|AT1G12740|AT1G14185|AT1G26380|AT1G26390|AT1G26400|AT1G26410|AT1G26420|AT1G28480|AT1G32350|AT1G48605|AT1G50520|AT1G50560|AT1G59730|AT1G62560|AT1G64950|AT1G65860|AT1G66130|AT1G69880|AT1G78490|AT2G02850|AT2G05620|AT2G25160|AT2G30540|AT2G30750|AT2G30770|AT2G32300|AT2G34810|AT2G46650|AT3G09580|AT3G09940|AT3G14620|AT3G20140|AT3G20940|AT3G22370|AT3G26180|AT3G26200|AT3G28740|AT3G62950|AT3G63380|AT4G01380|AT4G15300|AT4G15660|AT4G15670|AT4G15680|AT4G15690|AT4G15700|AT4G16310|AT4G22710|AT4G33040|AT4G37370|AT4G37410|AT5G08250|AT5G14070|AT5G18600|AT5G25130|AT5G36220|AT5G38450|AT5G39320|AT5G40370|AT5G42580|AT5G57220|AT5G61440	717	7.13E-08

20.03	transport facilities	38	2.97	AT1G01620|AT1G22530|AT1G61800|AT1G71140|AT1G73220|AT2G04050|AT2G24710|AT2G29120|AT2G36830|AT2G37170|AT2G37180|AT3G02850|AT3G16240|AT3G21180|AT3G26520|AT3G27070|AT3G45690|AT3G45700|AT3G45710|AT3G45720|AT3G61430|AT3G63380|AT4G04850|AT4G13420|AT4G17340|AT4G19030|AT4G23700|AT4G34580|AT4G35100|AT5G01180|AT5G02270|AT5G16530|AT5G17860|AT5G24030|AT5G44110|AT5G47450|AT5G48410|AT5G60660	600	2.17E-02
20.03.01	channel / pore class transport	16	1.25	AT1G01620|AT2G24710|AT2G29120|AT2G36830|AT2G37170|AT2G37180|AT3G02850|AT3G16240|AT3G26520|AT3G61430|AT4G17340|AT4G19030|AT4G35100|AT5G47450|AT5G48410|AT5G60660	123	1.28E-04
**30**	**CELLULAR COMMUNICATION/SIGNAL TRANSDUCTION MECHANISM**	59	4.62	AT1G03440|AT1G19050|AT1G22740|AT1G24530|AT1G53170|AT1G56650|AT1G57630|AT1G59940|AT1G67710|AT1G72260|AT1G72360|AT1G72900|AT1G72910|AT1G72920|AT1G72940|AT1G73165|AT2G06050|AT2G19660|AT2G20145|AT2G21880|AT2G28970|AT2G31081|AT2G31082|AT2G31085|AT2G31880|AT2G34180|AT2G36270|AT2G36830|AT2G40670|AT2G44840|AT2G46680|AT3G03530|AT3G12610|AT3G15354|AT3G16360|AT3G19850|AT3G23240|AT3G24500|AT3G48100|AT3G56070|AT4G14980|AT4G18510|AT4G19515|AT4G28490|AT4G31250|AT5G06860|AT5G06870|AT5G09290|AT5G12940|AT5G15230|AT5G17970|AT5G24100|AT5G45090|AT5G45350|AT5G47220|AT5G47230|AT5G49770|AT5G53500|AT5G62920	1283	4.44E-01
30.01	cellular signalling	35	2.74	AT1G19050|AT1G22740|AT1G24530|AT1G53170|AT1G59940|AT1G67710|AT1G72260|AT1G72360|AT2G06050|AT2G19660|AT2G21880|AT2G28970|AT2G31880|AT2G36270|AT2G36830|AT2G40670|AT2G44840|AT2G46680|AT3G03530|AT3G15354|AT3G16360|AT3G23240|AT3G24500|AT3G48100|AT4G14980|AT4G28490|AT4G31250|AT5G09290|AT5G15230|AT5G24100|AT5G47220|AT5G47230|AT5G49770|AT5G53500|AT5G62920	871	1.00E+00
30.01.05	enzyme mediated signal transduction	15	1.17	AT1G19050|AT1G22740|AT1G24530|AT1G59940|AT2G21880|AT2G28970|AT2G31880|AT3G15354|AT3G16360|AT4G28490|AT4G31250|AT5G24100|AT5G49770|AT5G53500|AT5G62920	446	1.00E+00
30.01.05.07	two-component signal transduction system (e.g. phosphorelay)	4	0.31	AT1G19050|AT1G59940|AT3G16360|AT5G62920	21	1.31E-02
30.01.09.08	hormone mediated signal transduction	18	1.41	AT1G19050|AT1G53170|AT1G59940|AT1G67710|AT1G72260|AT1G72360|AT2G36830|AT2G40670|AT2G44840|AT2G46680|AT3G16360|AT3G23240|AT3G24500|AT3G48100|AT5G15230|AT5G47220|AT5G47230|AT5G62920	205	5.29E-03

<table>
<tr><td>32</td><td>CELL RESCUE, DEFENSE AND VIRULENCE</td><td>189</td><td>14.8</td><td>AT1G01620|AT1G02920|AT1G02930|AT1G05240|AT1G05250|AT1G06830|
AT1G07400|AT1G17180|AT1G17190|AT1G17420|AT1G18330|AT1G19610|
AT1G19670|AT1G20620|AT1G28480|AT1G29395|AT1G30870|AT1G31580|
AT1G34510|AT1G48000|AT1G48605|AT1G49860|AT1G52040|AT1G52890|
AT1G53680|AT1G56650|AT1G57630|AT1G59700|AT1G59730|AT1G60740|
AT1G64160|AT1G65060|AT1G69880|AT1G69920|AT1G69930|AT1G72260|
AT1G72900|AT1G72910|AT1G72920|AT1G72940|AT1G74590|AT1G75280|
AT1G75830|AT1G77120|AT1G78290|AT1G78380|AT1G80840|AT2G02120|
AT2G02930|AT2G02990|AT2G03760|AT2G06050|AT2G14610|AT2G15220|
AT2G16060|AT2G17480|AT2G18140|AT2G18150|AT2G19190|AT2G20145|
AT2G21640|AT2G23680|AT2G24850|AT2G26010|AT2G26020|AT2G28190|
AT2G29440|AT2G29460|AT2G29470|AT2G30540|AT2G33380|AT2G34810|
AT2G36270|AT2G36830|AT2G37130|AT2G37170|AT2G37180|AT2G39040|
AT2G39800|AT2G42530|AT2G42540|AT2G43510|AT2G43530|AT2G46680|
AT2G47180|AT2G47190|AT2G48150|AT3G01190|AT3G03190|AT3G05890|
AT3G12500|AT3G12610|AT3G13650|AT3G14210|AT3G14440|AT3G17790|
AT3G22370|AT3G23240|AT3G23250|AT3G24500|AT3G25760|AT3G26520|
AT3G30775|AT3G45140|AT3G49110|AT3G49960|AT3G51910|AT3G55120|
AT3G57260|AT3G61430|AT3G62760|AT3G62950|AT4G02520|AT4G08770|
AT4G08780|AT4G10270|AT4G11210|AT4G11290|AT4G11600|AT4G12490|
AT4G15660|AT4G15670|AT4G15680|AT4G15690|AT4G15700|AT4G17340|
AT4G21830|AT4G22880|AT4G23190|AT4G23600|AT4G25470|AT4G25480|
AT4G25490|AT4G26010|AT4G27410|AT4G30170|AT4G30650|AT4G31870|
AT4G33040|AT4G34710|AT4G35100|AT4G37220|AT4G37760|AT4G38410|
AT5G05340|AT5G06720|AT5G06730|AT5G06860|AT5G06870|AT5G07990|
[illegible]</td><td>1425</td><td>2.88E-42</td></tr>
<tr><td>32.01</td><td>stress response</td><td>115</td><td>9.01</td><td>AT1G01620|AT1G02930|AT1G05240|AT1G05250|AT1G07400|AT1G17420|
AT1G18330|AT1G19670|AT1G29395|AT1G30870|AT1G34510|AT1G48000|
AT1G48605|AT1G52890|AT1G56650|AT1G65060|AT1G75280|AT1G77120|
AT1G78290|AT1G78380|AT2G02990|AT2G06050|AT2G16060|AT2G18140|
AT2G21640|AT2G23680|AT2G24850|AT2G28190|AT2G33380|AT2G34810|
AT2G36270|AT2G36830|AT2G37170|AT2G37180|AT2G39040|AT2G39800|
AT2G42530|AT2G42540|AT2G46680|AT2G47180|AT2G47190|AT2G48150|
AT3G01190|AT3G03190|AT3G05890|AT3G12610|AT3G14210|AT3G14440|
AT3G17790|AT3G22370|AT3G23250|AT3G24500|AT3G25760|AT3G26520|
AT3G30775|AT3G45140|AT3G49110|AT3G49960|AT3G51910|AT3G55120|
AT3G57260|AT3G61430|AT4G02520|AT4G08770|AT4G08780|AT4G10270|
AT4G11290|AT4G17340|AT4G21830|AT4G22880|AT4G23190|AT4G23600|
AT4G25470|AT4G25480|AT4G25490|AT4G26010|AT4G27410|AT4G30170|
AT4G30650|AT4G31870|AT4G34710|AT4G35100|AT4G37220|AT4G37760|
AT4G38410|AT5G05340|AT5G06720|AT5G06730|AT5G07990|AT5G08790|
AT5G13930|AT5G14130|AT5G14780|AT5G15180|AT5G15970|AT5G16970|
AT5G16980|AT5G17000|AT5G17820|AT5G20630|AT5G22410|AT5G24770|
AT5G25610|AT5G27760|AT5G37260|AT5G42180|AT5G43570|AT5G43580|
AT5G47230|AT5G47450|AT5G49630|AT5G52640|AT5G58390|AT5G64100|
AT5G67400</td><td>832</td><td>3.13E-27</td></tr>
</table>

32.01.01	oxidative stress response	45	3.52	AT1G02930|AT1G05240|AT1G05250|AT1G07400|AT1G30870|AT1G34510|AT1G75280|AT1G78380|AT2G18140|AT2G21640|AT2G28190|AT2G39040|AT2G47180|AT2G48150|AT3G01190|AT3G03190|AT3G17790|AT3G30775|AT3G49110|AT3G49960|AT4G08770|AT4G08780|AT4G11290|AT4G21830|AT4G23190|AT4G26010|AT4G30170|AT4G31870|AT4G34710|AT5G05340|AT5G06720|AT5G06730|AT5G13930|AT5G14130|AT5G15180|AT5G16970|AT5G16980|AT5G17000|AT5G17820|AT5G22410|AT5G24770|AT5G42180|AT5G58390|AT5G64100|AT5G67400	200	1.76E-19
32.01.03	osmotic and salt stress response	30	2.35	AT1G01620|AT1G18330|AT1G48000|AT1G48605|AT1G56650|AT1G77120|AT1G78290|AT2G33380|AT2G36270|AT2G36830|AT2G37170|AT2G37180|AT2G39800|AT2G42540|AT2G47190|AT3G14440|AT3G23250|AT3G26520|AT3G61430|AT4G17340|AT4G23600|AT4G30650|AT4G34710|AT4G35100|AT5G15970|AT5G24770|AT5G25610|AT5G37260|AT5G47450|AT5G49630	205	1.24E-08
32.01.06	cold shock response	15	1.17	AT1G29395|AT2G42530|AT2G42540|AT3G05890|AT3G14210|AT3G22370|AT3G57260|AT4G02520|AT4G25470|AT4G25480|AT4G25490|AT4G30650|AT5G15970|AT5G20630|AT5G47230	165	7.61E-03
32.01.13	electromagnetic waves stress response (e.g. UV, X-ray)	4	0.31	AT1G65060|AT3G12610|AT3G55120|AT5G07990	29	3.93E-02
32.05	disease, virulence and defense	42	3.29	AT1G17420|AT1G19610|AT1G31580|AT1G52040|AT1G57630|AT1G64160|AT1G72260|AT1G72900|AT1G72910|AT1G72920|AT1G72940|AT1G75830|AT1G80840|AT2G02120|AT2G03760|AT2G14610|AT2G15220|AT2G17480|AT2G19190|AT2G20145|AT2G26010|AT2G26020|AT2G37130|AT2G43510|AT2G43530|AT3G12500|AT3G12610|AT3G13650|AT3G23240|AT3G49110|AT4G11210|AT4G12490|AT5G06860|AT5G06870|AT5G13320|AT5G17970|AT5G36910|AT5G42510|AT5G44420|AT5G44430|AT5G45090|AT5G52640	460	1.21E-05
32.05.01	resistance proteins	1	0.07	AT3G12610	4	1.68E-01
32.07	detoxification	70	5.48	AT1G02920|AT1G02930|AT1G05240|AT1G05250|AT1G06830|AT1G17180|AT1G17190|AT1G20620|AT1G28480|AT1G30870|AT1G34510|AT1G49860|AT1G53680|AT1G56650|AT1G59700|AT1G59730|AT1G60740|AT1G69880|AT1G69920|AT1G69930|AT1G74590|AT1G78380|AT2G02930|AT2G18140|AT2G18150|AT2G28190|AT2G29440|AT2G29460|AT2G29470|AT2G30540|AT2G37130|AT2G39040|AT2G48150|AT3G01190|AT3G03190|AT3G49110|AT3G49960|AT3G62760|AT3G62950|AT4G02520|AT4G08770|AT4G08780|AT4G11290|AT4G11600|AT4G15660|AT4G15670|AT4G15680|AT4G15690|AT4G15700|AT4G26010|AT4G30170|AT4G31870|AT4G33040|AT5G05340|AT5G06720|AT5G06730|AT5G14070|AT5G14130|AT5G15180|AT5G17220|AT5G17820|AT5G18600|AT5G22410|AT5G36270|AT5G40370|AT5G42180|AT5G58390|AT5G61440|AT5G64100|AT5G67400	266	3.38E-34

32.07.03	detoxification by modification	7	0.54	AT4G15660|AT4G15670|AT4G15680|AT4G15690|AT4G15700|AT5G18600|AT5G40370	17	4.74E-06
32.07.07	oxygen and radical detoxification	70	5.48	AT1G02920|AT1G02930|AT1G05240|AT1G05250|AT1G06830|AT1G17180|AT1G17190|AT1G20620|AT1G28480|AT1G30870|AT1G34510|AT1G49860|AT1G53680|AT1G56650|AT1G59700|AT1G59730|AT1G60740|AT1G69880|AT1G69920|AT1G69930|AT1G74590|AT1G78380|AT2G02930|AT2G18140|AT2G18150|AT2G28190|AT2G29440|AT2G29460|AT2G29470|AT2G30540|AT2G37130|AT2G39040|AT2G48150|AT3G01190|AT3G03190|AT3G49110|AT3G49960|AT3G62760|AT3G62950|AT4G02520|AT4G08770|AT4G08780|AT4G11290|AT4G11600|AT4G15660|AT4G15670|AT4G15680|AT4G15690|AT4G15700|AT4G26010|AT4G30170|AT4G31870|AT4G33040|AT5G05340|AT5G06720|AT5G06730|AT5G14070|AT5G14130|AT5G15180|AT5G17220|AT5G17820|AT5G18600|AT5G22410|AT5G36270|AT5G40370|AT5G42180|AT5G58390|AT5G61440|AT5G64100|AT5G67400	261	8.99E-35
32.07.07.03	glutathione conjugation reaction	20	1.56	AT1G02920|AT1G02930|AT1G17180|AT1G17190|AT1G49860|AT1G53680|AT1G59700|AT1G69920|AT1G69930|AT1G74590|AT1G78380|AT2G02930|AT2G29440|AT2G29460|AT2G29470|AT3G03190|AT3G62760|AT4G02520|AT5G17220|AT5G36270	57	2.38E-13
32.07.07.05	peroxidase reaction	31	2.42	AT1G05240|AT1G05250|AT1G20620|AT1G30870|AT1G34510|AT2G18140|AT2G18150|AT2G37130|AT2G39040|AT2G48150|AT3G01190|AT3G49110|AT3G49960|AT4G08770|AT4G08780|AT4G11290|AT4G11600|AT4G26010|AT4G30170|AT4G31870|AT5G05340|AT5G06720|AT5G06730|AT5G14130|AT5G15180|AT5G17820|AT5G22410|AT5G42180|AT5G58390|AT5G64100|AT5G67400	94	5.09E-19
**34**	**INTERACTION WITH THE ENVIRONMENT**	165	12.9	AT1G01580|AT1G01620|AT1G02930|AT1G03870|AT1G04240|AT1G07400|AT1G07610|AT1G08430|AT1G17345|AT1G18330|AT1G19050|AT1G29395|AT1G29490|AT1G48000|AT1G48605|AT1G52250|AT1G52890|AT1G56650|AT1G59940|AT1G64780|AT1G65060|AT1G66380|AT1G67710|AT1G72430|AT1G75040|AT1G75580|AT1G75750|AT1G75780|AT1G77120|AT1G78290|AT1G78380|AT1G80840|AT2G06050|AT2G14580|AT2G16060|AT2G16580|AT2G17310|AT2G18010|AT2G18150|AT2G19190|AT2G23170|AT2G24710|AT2G24850|AT2G28190|AT2G28670|AT2G29120|AT2G30520|AT2G33380|AT2G34810|AT2G36270|AT2G36830|AT2G37130|AT2G37170|AT2G37180|AT2G38230|AT2G39350|AT2G39800|AT2G40080|AT2G40340|AT2G40670|AT2G42530|AT2G42540|AT2G43510|AT2G46680|AT2G47180|AT2G47190|AT3G02850|AT3G02885|AT3G04720|AT3G05890|AT3G09390|AT3G09940|AT3G12500|AT3G12610|AT3G12955|AT3G14210|AT3G14440|AT3G15210|AT3G17790|AT3G18780|AT3G19850|AT3G22231|AT3G22370|AT3G23250|AT3G24500|AT3G25760|AT3G26520|AT3G26830|AT3G28210|AT3G28580|AT3G28930|AT3G44300|AT3G45140|AT3G48100|AT3G48920|AT3G49110|AT3G51240|AT3G51910|AT3G55120|AT3G57240|AT3G57260|AT3G60140|AT3G61430|AT4G02520|AT4G04850|AT4G11650|AT4G12490|AT4G12550|AT4G12730|AT4G14400|AT4G15480|AT4G17340|AT4G19690|AT4G22880|AT4G23170|AT4G23190|AT4G23600|AT4G24120|AT4G25470|AT4G25480|AT4G25490|AT4G27410|AT4G30650|AT4G34710|AT4G34790|AT4G34800|AT4G35100|AT4G36110|AT4G36220|AT4G37390|AT4G37760|AT4G37990|AT4G38410|AT4G38860|AT5G03240|AT5G03310|AT5G07990|AT5G08790|AT5G10140|AT5G13320|AT5G13370|AT5G13930|AT5G15230|AT5G15970|AT5G20250|AT5G20630|AT5G24770|AT5G24860|AT5G25610|AT5G27420|[illegible]	1651	1.36E-22

34.01	homeostasis	8	0.62	AT1G01580|AT2G24710|AT2G29120|AT3G09390|AT3G17790|AT4G04850|AT4G19690|AT5G48410	77	2.21E-02
34.01.01	homeostasis of cations	6	0.47	AT1G01580|AT2G24710|AT2G29120|AT3G09390|AT4G19690|AT5G48410	60	5.19E-02
34.01.01.01	homeostasis of metal ions (Na, K, Ca etc.)	6	0.47	AT1G01580|AT2G24710|AT2G29120|AT3G09390|AT4G19690|AT5G48410	58	4.52E-02
34.11	cellular sensing and response to external stimulus	156	12.2	AT1G01620|AT1G02930|AT1G04240|AT1G07400|AT1G07610|AT1G08430|AT1G17345|AT1G18330|AT1G19050|AT1G29395|AT1G29490|AT1G48000|AT1G48605|AT1G52890|AT1G56650|AT1G59940|AT1G64780|AT1G65060|AT1G66380|AT1G67710|AT1G72430|AT1G75040|AT1G75580|AT1G75750|AT1G75780|AT1G77120|AT1G78290|AT1G78380|AT1G80840|AT2G06050|AT2G14580|AT2G16060|AT2G16580|AT2G17310|AT2G18010|AT2G18150|AT2G19190|AT2G23170|AT2G24710|AT2G24850|AT2G28190|AT2G28670|AT2G29120|AT2G30520|AT2G33380|AT2G34810|AT2G36270|AT2G36830|AT2G37130|AT2G37170|AT2G37180|AT2G38230|AT2G39350|AT2G39800|AT2G40080|AT2G40340|AT2G40670|AT2G42530|AT2G42540|AT2G43510|AT2G46680|AT2G47180|AT2G47190|AT3G02850|AT3G02885|AT3G04720|AT3G05890|AT3G09940|AT3G12500|AT3G12610|AT3G12955|AT3G14210|AT3G14440|AT3G15210|AT3G18780|AT3G19850|AT3G22231|AT3G22370|AT3G23250|AT3G24500|AT3G25760|AT3G26520|AT3G26830|AT3G28210|AT3G28580|AT3G28930|AT3G44300|AT3G45140|AT3G48100|AT3G48920|AT3G49110|AT3G51240|AT3G51910|AT3G55120|AT3G57240|AT3G57260|AT3G60140|AT3G61430|AT4G02520|AT4G11650|AT4G12490|AT4G12550|AT4G14400|AT4G15480|AT4G17340|AT4G22880|AT4G23170|AT4G23190|AT4G23600|AT4G24120|AT4G25470|AT4G25480|AT4G25490|AT4G27410|AT4G30650|AT4G34710|AT4G34790|AT4G34800|AT4G35100|AT4G36110|AT4G36220|AT4G37390|AT4G37760|AT4G37990|AT4G38410|AT4G38860|AT5G03240|AT5G03310|AT5G07990|AT5G08790|AT5G10140|AT5G13320|AT5G13370|AT5G13930|AT5G15230|AT5G15970|AT5G20250|AT5G20630|AT5G24770|AT5G24860|AT5G25610|AT5G27420|AT5G37260|AT5G44420|AT5G47230|AT5G47450|AT5G48410|AT5G49630|AT5G52640|AT5G54490|[illegible]	1489	2.00E-23
34.11.01	photoperception and response	26	2.03	AT1G65060|AT1G75040|AT1G75780|AT2G24710|AT2G28190|AT2G29120|AT2G30520|AT2G38230|AT2G40080|AT2G47180|AT3G12610|AT3G18780|AT3G19850|AT3G49110|AT3G51240|AT3G51910|AT3G55120|AT3G60140|AT4G15480|AT4G36220|AT5G03240|AT5G07990|AT5G08790|AT5G13930|AT5G20250|AT5G48410	265	1.70E-04

34.11.03	chemoperception and response	106	8.3	AT1G01620|AT1G02930|AT1G04240|AT1G07610|AT1G08430|AT1G17345|AT1G18330|AT1G19050|AT1G29395|AT1G29490|AT1G48000|AT1G48605|AT1G52890|AT1G56650|AT1G59940|AT1G66380|AT1G67710|AT1G72430|AT1G75580|AT1G75750|AT1G77120|AT1G78290|AT1G78380|AT1G80840|AT2G14580|AT2G16580|AT2G18010|AT2G23170|AT2G24850|AT2G28190|AT2G33380|AT2G34810|AT2G36270|AT2G36830|AT2G37170|AT2G37180|AT2G39800|AT2G40670|AT2G42540|AT2G46680|AT2G47190|AT3G02850|AT3G02885|AT3G04720|AT3G09940|AT3G12610|AT3G12955|AT3G14440|AT3G15210|AT3G18780|AT3G23250|AT3G24500|AT3G25760|AT3G26520|AT3G26830|AT3G28210|AT3G28580|AT3G45140|AT3G48100|AT3G48920|AT3G60140|AT3G61430|AT4G12550|AT4G14400|AT4G17340|AT4G22880|AT4G23170|AT4G23600|AT4G24120|AT4G25480|AT4G25490|AT4G27410|AT4G30650|AT4G34710|AT4G34790|AT4G34800|AT4G35100|AT4G36110|AT4G37390|AT4G37760|AT4G38410|AT4G38860|AT5G03310|AT5G08790|AT5G13320|AT5G13370|AT5G13930|AT5G15230|AT5G15970|AT5G20250|AT5G24770|AT5G24860|AT5G25610|AT5G27420|AT5G37260|AT5G44420|AT5G47450|AT5G49630|AT5G52640|AT5G54490|AT5G54610|AT5G59220|AT5G59310|AT5G59320|AT5G62920|AT5G66700	841	5.65E-22
34.11.03.12	water response	25	1.95	AT1G01620|AT1G02930|AT1G29395|AT1G52890|AT1G78290|AT1G78380|AT2G33380|AT2G36270|AT2G37170|AT2G37180|AT2G39800|AT2G46680|AT2G47190|AT3G14440|AT3G24500|AT3G25760|AT3G45140|AT3G61430|AT4G25480|AT4G25490|AT4G27410|AT4G38410|AT5G15970|AT5G25610|AT5G49630	119	9.23E-11
34.11.03.13	osmosensing and response	30	2.35	AT1G01620|AT1G18330|AT1G48000|AT1G48605|AT1G56650|AT1G77120|AT1G78290|AT2G33380|AT2G36270|AT2G36830|AT2G37170|AT2G37180|AT2G39800|AT2G42540|AT2G47190|AT3G14440|AT3G23250|AT3G26520|AT3G61430|AT4G17340|AT4G23600|AT4G30650|AT4G34710|AT4G35100|AT5G15970|AT5G24770|AT5G25610|AT5G37260|AT5G47450|AT5G49630	208	1.74E-08
34.11.10	response to biotic stimulus	27	2.11	AT1G64780|AT1G80840|AT2G17310|AT2G18150|AT2G19190|AT2G28670|AT2G37130|AT2G39350|AT2G43510|AT3G04720|AT3G12500|AT3G14210|AT3G22231|AT3G28930|AT3G44300|AT3G49110|AT3G57240|AT4G11650|AT4G12490|AT4G14400|AT4G23190|AT4G37990|AT5G08790|AT5G13320|AT5G24770|AT5G44420|AT5G52640	296	4.17E-04

**36**	**SYSTEMIC INTERACTION WITH THE ENVIRONMENT**	92	7.21	AT1G03620|AT1G04240|AT1G17345|AT1G17420|AT1G18330|AT1G19050|AT1G29395|AT1G29490|AT1G48000|AT1G56650|AT1G59940|AT1G66380|AT1G67710|AT1G72430|AT1G75040|AT1G75580|AT1G75750|AT1G80840|AT2G02990|AT2G06050|AT2G14580|AT2G14610|AT2G16580|AT2G18010|AT2G22330|AT2G23170|AT2G24850|AT2G33380|AT2G34810|AT2G36270|AT2G39800|AT2G40670|AT2G46680|AT2G47190|AT3G02850|AT3G02885|AT3G04720|AT3G09940|AT3G12500|AT3G12955|AT3G14210|AT3G15210|AT3G18780|AT3G23250|AT3G24500|AT3G26830|AT3G28210|AT3G28580|AT3G45140|AT3G48100|AT3G48920|AT3G57260|AT4G10270|AT4G11650|AT4G12550|AT4G14400|AT4G22880|AT4G23170|AT4G23190|AT4G23600|AT4G27410|AT4G34710|AT4G34790|AT4G34800|AT4G36110|AT4G37390|AT4G37760|AT4G38860|AT5G03310|AT5G08790|AT5G13320|AT5G13370|AT5G13930|AT5G14780|AT5G15230|AT5G15970|AT5G24770|AT5G24860|AT5G25610|AT5G27420|AT5G37260|AT5G43570|AT5G43580|AT5G44420|AT5G47220|AT5G54490|AT5G54610|AT5G59220|AT5G59310|AT5G59320|AT5G62920|AT5G66700	757	3.79E-18
36.20	plant / fungal specific systemic sensing and response	84	6.58	AT1G04240|AT1G17345|AT1G18330|AT1G19050|AT1G29395|AT1G29490|AT1G48000|AT1G56650|AT1G59940|AT1G66380|AT1G67710|AT1G72430|AT1G75040|AT1G75580|AT1G75750|AT1G80840|AT2G14580|AT2G14610|AT2G16580|AT2G18010|AT2G22330|AT2G23170|AT2G24850|AT2G33380|AT2G34810|AT2G36270|AT2G39800|AT2G40670|AT2G46680|AT2G47190|AT3G02850|AT3G02885|AT3G04720|AT3G09940|AT3G12500|AT3G12955|AT3G14210|AT3G15210|AT3G18780|AT3G23250|AT3G24500|AT3G26830|AT3G28210|AT3G28580|AT3G45140|AT3G48100|AT3G48920|AT3G57260|AT4G11650|AT4G12550|AT4G14400|AT4G22880|AT4G23170|AT4G23190|AT4G23600|AT4G27410|AT4G34710|AT4G34790|AT4G34800|AT4G36110|AT4G37390|AT4G37760|AT4G38860|AT5G03310|AT5G08790|AT5G13320|AT5G13370|AT5G13930|AT5G15230|AT5G15970|AT5G24770|AT5G24860|AT5G25610|AT5G27420|AT5G37260|AT5G44420|AT5G47220|AT5G54490|AT5G54610|AT5G59220|AT5G59310|AT5G59320|AT5G62920|AT5G66700	692	1.24E-16
36.20.16	plant defense response	15	1.17	AT1G75040|AT2G14610|AT2G22330|AT3G12500|AT3G14210|AT3G15210|AT3G26830|AT3G57260|AT4G11650|AT4G14400|AT4G23190|AT4G23600|AT5G24770|AT5G44420|AT5G47220	124	4.56E-04
36.20.16.03	jasmonic acid/ethylene dependent systemic resistance	4	0.31	AT3G12500|AT3G15210|AT5G44420|AT5G47220	25	2.41E-02

36.20.18	plant hormonal regulation	75	5.87	AT1G04240|AT1G17345|AT1G18330|AT1G19050|AT1G29395|AT1G29490|AT1G48000|AT1G56650|AT1G59940|AT1G66380|AT1G67710|AT1G72430|AT1G75580|AT1G75750|AT1G80840|AT2G14580|AT2G16580|AT2G18010|AT2G23170|AT2G24850|AT2G33380|AT2G34810|AT2G36270|AT2G39800|AT2G40670|AT2G46680|AT2G47190|AT3G02850|AT3G02885|AT3G04720|AT3G09940|AT3G12955|AT3G15210|AT3G18780|AT3G23250|AT3G24500|AT3G26830|AT3G28210|AT3G28580|AT3G45140|AT3G48100|AT3G48920|AT4G12550|AT4G14400|AT4G22880|AT4G23170|AT4G23600|AT4G27410|AT4G34710|AT4G34790|AT4G34800|AT4G36110|AT4G37390|AT4G37760|AT4G38860|AT5G03310|AT5G08790|AT5G13320|AT5G13370|AT5G13930|AT5G15230|AT5G15970|AT5G24770|AT5G24860|AT5G25610|AT5G27420|AT5G37260|AT5G44420|AT5G54490|AT5G54610|AT5G59220|AT5G59310|AT5G59320|AT5G62920|AT5G66700	589	4.26E-16
36.20.18.01	auxin response	22	1.72	AT1G04240|AT1G17345|AT1G29490|AT1G72430|AT1G75580|AT2G16580|AT2G18010|AT2G23170|AT3G12955|AT3G23250|AT4G12550|AT4G34790|AT4G34800|AT4G36110|AT4G37390|AT4G38860|AT5G03310|AT5G13320|AT5G13370|AT5G37260|AT5G54490|AT5G66700	244	1.58E-03
36.20.18.02	ethylen response	9	0.7	AT1G18330|AT2G14580|AT2G47190|AT3G04720|AT3G15210|AT3G23250|AT3G24500|AT5G37260|AT5G44420	100	3.59E-02
36.20.18.04	cytokinin response	7	0.54	AT1G19050|AT1G59940|AT1G67710|AT2G40670|AT3G18780|AT3G48100|AT5G62920	49	5.98E-03
36.20.18.05	abscisic acid response	24	1.88	AT1G29395|AT1G48000|AT1G75750|AT2G33380|AT2G36270|AT2G39800|AT2G46680|AT2G47190|AT3G02850|AT3G15210|AT3G24500|AT3G26830|AT3G28210|AT3G28580|AT4G23600|AT4G27410|AT4G34710|AT5G15970|AT5G25610|AT5G27420|AT5G37260|AT5G59220|AT5G59310|AT5G59320	161	2.44E-07
36.25	animal specific systemic sensing and response	18	1.41	AT1G03620|AT1G17420|AT2G02990|AT2G06050|AT2G24850|AT2G34810|AT3G45140|AT4G10270|AT4G22880|AT4G23600|AT4G34710|AT4G37760|AT5G08790|AT5G13930|AT5G14780|AT5G24770|AT5G43570|AT5G43580	107	1.28E-06
36.25.16	immune response	18	1.41	AT1G03620|AT1G17420|AT2G02990|AT2G06050|AT2G24850|AT2G34810|AT3G45140|AT4G10270|AT4G22880|AT4G23600|AT4G34710|AT4G37760|AT5G08790|AT5G13930|AT5G14780|AT5G24770|AT5G43570|AT5G43580	100	4.55E-07
36.25.16.08	response to wounding	17	1.33	AT1G17420|AT2G02990|AT2G06050|AT2G24850|AT2G34810|AT3G45140|AT4G10270|AT4G22880|AT4G23600|AT4G34710|AT4G37760|AT5G08790|AT5G13930|AT5G14780|AT5G24770|AT5G43570|AT5G43580	80	7.68E-08
**40**	**CELL FATE**	31	2.42	AT1G17420|AT1G19050|AT1G20190|AT1G26770|AT1G59940|AT1G62980|AT1G67710|AT1G75750|AT1G75780|AT2G02990|AT2G17480|AT2G26560|AT2G29350|AT2G40670|AT3G18000|AT3G18780|AT3G45960|AT3G48100|AT3G49110|AT3G60140|AT4G14400|AT4G17030|AT4G23410|AT4G37490|AT4G37990|AT5G08790|AT5G19530|AT5G20250|AT5G25810|AT5G35630|AT5G62920	450	1.27E-02
40.02.03	activity of intercellular mediators	7	0.54	AT1G19050|AT1G59940|AT1G67710|AT2G40670|AT3G18780|AT3G48100|AT5G62920	46	4.18E-03

40.02.03.01	cytokines (interleukines, colony stimulating factors, etc.)	7	0.54	AT1G19050|AT1G59940|AT1G67710|AT2G40670|AT3G18780|AT3G48100|AT5G62920	46	4.18E-03
40.20	cell aging	6	0.47	AT2G02990|AT2G29350|AT3G60140|AT4G23410|AT5G20250|AT5G35630	49	2.18E-02
**42**	**BIOGENESIS OF CELLULAR COMPONENTS**	90	7.05	AT1G02810|AT1G19210|AT1G19510|AT1G20190|AT1G21910|AT1G26250|AT1G26770|AT1G43160|AT1G48000|AT1G52250|AT1G53170|AT1G54970|AT1G56650|AT1G60920|AT1G62980|AT1G66230|AT1G66380|AT1G67750|AT1G72360|AT1G72830|AT1G80840|AT2G15390|AT2G22630|AT2G24980|AT2G26440|AT2G28700|AT2G36270|AT2G38080|AT2G38530|AT2G40340|AT2G40750|AT2G42170|AT2G43220|AT2G43570|AT2G43580|AT2G43590|AT2G44840|AT2G46400|AT2G46680|AT2G47520|AT3G01970|AT3G05690|AT3G10710|AT3G11580|AT3G13760|AT3G15210|AT3G18780|AT3G19430|AT3G23240|AT3G23250|AT3G24310|AT3G28730|AT3G45960|AT3G47430|AT3G47540|AT3G48360|AT3G48920|AT3G49760|AT3G54580|AT3G54590|AT4G02330|AT4G08410|AT4G13130|AT4G13620|AT4G17030|AT4G17460|AT4G22880|AT4G23810|AT4G25470|AT4G25480|AT4G25910|AT4G33220|AT4G34410|AT5G02350|AT5G04310|AT5G04960|AT5G06510|AT5G06640|AT5G14750|AT5G15830|AT5G20860|AT5G21960|AT5G28770|AT5G47220|AT5G47230|AT5G51990|AT5G62150|AT5G63160|AT5G64810|AT5G66700	1554	8.08E-03
42.01	cell wall	29	2.27	AT1G02810|AT1G20190|AT1G26250|AT1G26770|AT1G54970|AT1G62980|AT1G67750|AT2G15390|AT2G24980|AT2G26440|AT2G38080|AT2G43570|AT2G43580|AT2G43590|AT3G10710|AT3G19430|AT3G45960|AT3G47540|AT3G54580|AT3G54590|AT4G02330|AT4G08410|AT4G17030|AT4G33220|AT5G04310|AT5G04960|AT5G06640|AT5G20860|AT5G62150	263	8.43E-06
42.30	prokaryotic cytoplasmic membrane	1	0.07	AT2G38530	1	4.49E-02
42.34	prokaryotic cell envelope structures	5	0.39	AT2G43570|AT2G43580|AT2G43590|AT3G47540|AT5G62150	27	6.41E-03
42.34.07	peptidoglycan layer or other prokaryotic cell wall	5	0.39	AT2G43570|AT2G43580|AT2G43590|AT3G47540|AT5G62150	21	2.02E-03

**70**	**SUBCELLULAR LOCALIZATION**	449	35.1	AT1G01620|AT1G02470|AT1G02810|AT1G02920|AT1G02930|AT1G03620|AT1G03870|AT1G04240|AT1G05240|AT1G06120|AT1G06350|AT1G07610|AT1G09932|AT1G12740|AT1G13300|AT1G16400|AT1G16410|AT1G17180|AT1G17190|AT1G17345|AT1G17420|AT1G17745|AT1G18330|AT1G19210|AT1G19510|AT1G20190|AT1G20620|AT1G20990|AT1G21100|AT1G21910|AT1G22230|AT1G22460|AT1G22740|AT1G24530|AT1G24575|AT1G26250|AT1G26770|AT1G26945|AT1G29490|AT1G31320|AT1G31580|AT1G32350|AT1G33030|AT1G35140|AT1G35910|AT1G43160|AT1G47510|AT1G47840|AT1G47960|AT1G48000|AT1G48325|AT1G49860|AT1G50730|AT1G52250|AT1G52700|AT1G52750|AT1G53030|AT1G53170|AT1G53680|AT1G54740|AT1G54970|AT1G55380|AT1G56650|AT1G58370|AT1G58420|AT1G59700|AT1G60190|AT1G60920|AT1G60970|AT1G61340|AT1G61800|AT1G62560|AT1G62975|AT1G62980|AT1G65310|AT1G65690|AT1G66130|AT1G66230|AT1G66380|AT1G66600|AT1G67710|AT1G67750|AT1G68010|AT1G68110|AT1G68450|AT1G69920|AT1G69930|AT1G70810|AT1G72360|AT1G72430|AT1G72610|AT1G72830|AT1G73120|AT1G74010|AT1G74080|AT1G74590|AT1G75750|AT1G75780|AT1G75830|AT1G77530|AT1G78120|AT1G78380|AT1G80130|AT1G80380|AT1G80840|AT2G01900|AT2G02850|AT2G02930|AT2G02990|AT2G05500|AT2G06050|AT2G14580|AT2G14610|AT2G15327|AT2G15400|AT2G15420|AT2G15430|AT2G16440|AT2G16450|AT2G16580|AT2G17070|AT2G17210|AT2G17270|AT2G17480|AT2G17510|AT2G17525|AT2G18010|AT2G18200|AT2G18210|AT2G18660|AT2G18700|AT2G19280|AT2G19970|AT2G19990|AT2G21640|AT2G22330|AT2G22500|AT2G22630|AT2G22880|AT2G23130|AT2G24980|AT2G25625|AT2G26010|AT2G26020|AT2G26440|AT2G26530|AT2G26560|AT2G27535|AT2G28190|AT2G28400|[illegible]	10531	1.00E+00
70.01	cell wall	56	4.38	AT1G02810|AT1G05240|AT1G20190|AT1G24530|AT1G26250|AT1G26770|AT1G31580|AT1G35140|AT1G47960|AT1G54970|AT1G58370|AT1G62980|AT1G65310|AT1G67750|AT1G74010|AT1G75750|AT1G75830|AT2G24980|AT2G26010|AT2G26020|AT2G26440|AT2G38540|AT3G10710|AT3G15354|AT3G16240|AT3G18080|AT3G19430|AT3G45960|AT3G49110|AT3G49330|AT3G54580|AT3G54590|AT3G61820|AT4G02330|AT4G08410|AT4G17030|AT4G25250|AT4G33220|AT5G04310|AT5G04960|AT5G06640|AT5G06860|AT5G06870|AT5G17820|AT5G18470|AT5G20860|AT5G22460|AT5G25460|AT5G42180|AT5G44130|AT5G44420|AT5G44430|AT5G51520|AT5G53500|AT5G59320|AT5G62340	431	1.05E-12
70.27	extracellular / secretion proteins	21	1.64	AT1G20190|AT1G62980|AT1G72610|AT2G02850|AT2G02990|AT2G14580|AT2G14610|AT2G18660|AT2G19970|AT2G19990|AT2G41280|AT4G07820|AT4G14630|AT4G17030|AT4G25780|AT4G25790|AT4G30320|AT4G33720|AT5G20630|AT5G39130|AT5G57625	99	2.41E-09
