# Supplementary material for: Alkamides Activate Jasmonic Acid Biosynthesis and Signaling Pathways and Confer Resistance to Botrytis cinerea in Arabidopsis thaliana
Source: PLoS One. 2011 Nov 4;6(11):e27251. doi: 10.1371/journal.pone.0027251 (PMC3208606; doi:10.1371/journal.pone.0027251)
Supplement: Table S3 — Sequences of oligonucleotides used as PCR primers for quantitative expression analysis. (PDF) [file pone.0027251.s007.pdf]

Table S3. *N*-isobutyl decanamide regulated genes used for qRT-PCR.

Locus	Predicted function	Forward	Reverse
At3g18780	actin 2/7	CGTACAACCGGTATTGTGCTGAT	GCTTGGTGCAAGTGCTGTGATTTC
At1g20990	DC1 Zinc finger	GCTGCGCCATGTTATCTCCTTCC	ACCCTCCCCGTTCGCTTACCTC
At1g66600	WRKY63	TCGTCAACCACACCATGAGTCT	CTCCAGGTGAAGCCGTCATC
At4g30290	xyloglucosyl transferase	GGGGTGATGGTCGTGGAAAGATT	CCAGCAGAGTTACCAGGGACAAGTT
At2g38600	acid phosphatase	TCTCCAGCAATACAACCTGTTCTT	CCTCATAATCAGCCTTTCATAACCA
At3g47480	calcium-binding EF	CTTTTCCCTTTCGTTTCCATCTC	TCGATTATCGCCTTGACTTCTTC
At2g29350	senescence-associated SAG13	CAATATTCAAGCCGACCACAGA	ACGACTCCAGCAGCAGAGGATA
At4g15690	glutaredoxin	AGACTTTGGCGTGAACCCGACTATC	AAGGGAGCGGTTGAGATGGAGACT
At5g19470	MutT/nudix kinase	TGTCCCACTCCCACCTCCTT	CAAAAACATCGTTCCAGGTGAA
At1g64780	ammonium transporter 1	TCCGCCTACCTTGTCTTTGC	ACCAAAGGCGAATGCGAATC
At5g59320	lipid transfer LTP3	ATGGCTTTCGCTTTGAGGTTCTTC	GTTGGCGGTCTGGTGTGGTTTTA
At4g02330	pectinesterase	CGTTCGAGACGGTCAATATCACT	TGTCGTTGATTAGAGGCAATGC
At1g49860	glutathione S-transferase	CCATATCAAGGTTTAGCCACAGACGA	GCAAGATGATGCAGATCCGCTAA
At4g19690	iron-responsive transporter	CGTGCGTCAACAAAGCTAAAGC	CGGAGGCGAAAGACTTAATGATA
At1g52890	no apical meristem NAM	CCACGGTCTTGCGGATACTTCTAACT	ACCCGTGACTGCTCTCGACTTCCTC
At5g53820	ABA-inducible/LEA domain	CCAAGCTAAAGGCCAAACTCA	ACTCGCACCTTGTGCCTTCT
At5g44420	plant defensin protein PDF1.2a	TCATCACCCTTATCTTCGCTGCTCTT	ATGATCCATGTTTGGCTCCTTCAAG
At5g38910	germin protein LEA7	TTTACGACAGAACCCACCTCACA	GGAAACACAAAGACATCACCCTCGTA
At4g26280	sulfotransferase	ACAATCCTCACAACCTCCTCTCA	CAAACGATATGCCAACGTGACA
At5g06510	CCAAT containing HAP2	TGCATCACTCACGCCATCTC	GACTCATTTGCTTCCCTCGATGA
At3g45140	lipoxigenase LOX2	AGTCTTGTCAACGGAGGTGGAAT	CCATGTTCTGCGGTCTTATCTTC
At1g17420	lipoxigenase LOX3	GGCAAAGAGGTTCCTTACCC	CTTTGAGCGACGGAATTAGG
At1g30135	jasmonate-ZIM-domain JAZ8	CAGCTTCCAAATCCAAAAGC	TATCGTCGTGAATGGTACGG
At3g25770	allene oxide cyclase AOC2	GCCAAGAAGAACCTCACTGC	TCCGAGACCGAACATTAAGC
At5g42650	allene oxide synthase AOS	GACCAATCAAAGACCGTTGG	ATCGAATAAAACCGGGAAGC
At5g24770	vegetative storage VSP2	GAAAACCATCTTTGGGAACG	CGGTTTTGGAGTCGTATTGG
At2g06050	OPR3	GGAAGCAGTTCACGCTAAGG	GAGGTTTCGGGTACTTCACG
At5g47220	ERF2	TAATTCCGGTGAACCTGACC	CACGTGTCTCATCACCAACC
At4g30140	GDSL-motif lipase	CATCGGAAGCAACGATTACC	ACCGAACAACGCTACATTCC
At3g26830	phytoalexin deficient PAD3	GCAAGAGAACGATGGAGATG	TCTTGTCCCCAAGTGTTGTC
	B. cinerea actinA	GGTAACATTGTTATGTCTGG	CTTGACCTTCATCGACG
